# The GPR139 agonist TAK-041 produces time-dependent alterations to cerebral blood flow and reward system function in patients with schizophrenia: a randomised placebo-controlled trial

**DOI:** 10.1007/s00213-025-06884-x

**Published:** 2025-08-16

**Authors:** Peter C. T. Hawkins, Adam J. Schwarz, James M. Stone, Fiona Pepper, James Gilleen, Sam Gijsen, Ndabezinhle Mazibuko, Dimitrios Arkilo, Wei Yin, Jingtao Wu, Polyna Khudyakov, Rhett Behrje, Laura Rosen, Joel Posener, Mitul A. Mehta, Antonio Laurenza

**Affiliations:** 1 Centre for Neuroimaging Sciences, Institute of Psychiatry, Psychology & Neurosciences, King’s College London, London, SE5 8AF UK; 2 Takeda Pharmaceuticals Ltd, Cambridge, MA 02139 USA; 3 Brighton and Sussex Medical School, University of Sussex, Brighton, UK

## Abstract

The negative and cognitive symptoms of schizophrenia are predictive of quality of life and functional recovery, but there are no approved drug options that target these symptoms directly. TAK-041 (also known as NBI-1065846) is a selective and potent small-molecule agonist of GPR139, an orphan G-protein-coupled receptor, which has been shown to reverse deficits related to negative and cognitive symptoms in animal models. In this proof-of-activity study to evaluate the effects of TAK-041 on motivational anhedonia, 23 adults with schizophrenia experiencing moderate to severe negative symptoms were administered a single 40 mg or 160 mg dose of TAK-041 in a randomised, double-blind, placebo-controlled, two-period crossover design. Functional magnetic resonance imaging (fMRI) assessment of reward function (monetary incentive delay [MID] task) and cerebral blood flow (CBF), as well as cognitive assessment with the Brief Assessment of Cognition in Schizophrenia (BACS) tool, were performed 3 h post dose and after 14 days. There was no significant effect of TAK-041 compared with placebo on BACS score at either timepoint. There was also no significant effect of TAK-041 on MID fMRI at day 1; however, at day 14, TAK-041 produced an increase in reward anticipatory activity in the ventral striatum compared with placebo. There was a significant decrease in CBF throughout the brain at day 1 that then reversed, resulting in a relative increase at day 14, compared with placebo. At day 14 there was a significant interaction between drug and CBF on reward anticipatory BOLD, indicating that drug-related changes to CBF may partially contribute to the observed BOLD effects (although the main effect of drug on the BOLD signal remained significant after including CBF as a covariate in the model). These data indicate that TAK-041 produces detectable changes in both CBF and reward task related fMRI signal in brain regions linked with symptoms and treatment response in schizophrenia. IND Number: 130074| EudraCT Number: 2017-001084-20| Clincaltrails.gov registry: NCT03319953.

**Supplementary Information:**

The online version contains supplementary material available at 10.1007/s00213-025-06884-x.

## Introduction

Despite decades of research into alternative drug treatments for schizophrenia, the dopamine (DA) D2 receptor remains the primary direct therapeutic target of current medications. Although effective for treating psychosis, these treatments appear to have little significant clinical impact on the negative symptoms of schizophrenia (such as anhedonia, loss of motivation and reduced interest in social interaction) and unclear efficacy for the cognitive deficits frequently present in the disorder (Fusar-Poli et al. 2015; Sakurai et al. 2013). Negative and cognitive symptoms are highly predictive of quality of life and functional recovery (Hunter and Barry 2012) and represent a significant unmet need in clinical practice and patient outcome. This has driven research into the development of novel pharmacological targets and interventions that seek to directly address these symptoms.

GPR139 is an orphan G-protein-coupled receptor that is expressed almost exclusively in the central nervous system. Understanding of the physiological function of GPR139 is incomplete, but human and animal genetic data have implicated it in schizophrenia. Copy number variants of GPR139 have been reported in the affected twin in monozygotic human twins discordant for schizophrenia (Castellani et al. 2014). In experimental animals, GPR139 −/− knockout mice were found to be significantly impaired on tasks related to social interaction and operant conditioning (Dao et al. 2022), while agonism of GPR139 was observed to reverse deficits in models of schizophrenia including cognition in a sub-chronic phencyclidine induced attentional set-shifting paradigm (Atienza et al. 2018).

TAK-041 (also known as NBI-1065846) is an investigational, highly selective and potent small-molecule agonist of GPR139 previously developed by Takeda Pharmaceuticals that has shown utility in reversing deficits related to negative and cognitive symptoms in animal models (Münster et al. 2022). In Balb/C and Poly [I: C] models, TAK-041 dosed either acutely or sub-chronically mitigated the social deficits occurring in both models as measured by a three-chambered sniffing index test (Reichard et al. 2021).

The only neuroimaging study examining the effect of TAK-041 in humans to date reported that 20 mg and 40 mg doses of the compound significantly attenuated amphetamine-induced DA release in a dose-response fashion in the putamen and ventral striatum (VS) (Rabiner et al. 2021). These data are particularly relevant given the consistent finding throughout the literature of schizophrenia patients exhibiting increased dopaminergic activity in the striatum (Howes et al. 2012).

The data summarised above provide a rationale to assess the agonism of GPR139 in patients with schizophrenia with negative and cognitive symptoms. In this proof-of-activity report, we describe an experimental medicine study (NCT03319953) assessing TAK-041 using (1) task-induced fMRI blood oxygen level-dependant (BOLD) assessment of reward function and (2) neuropsychological assessment of cognition in patients with schizophrenia with moderate to severe negative symptoms. The monetary incentive delay (MID) task was employed to assess reward function in the brain, as aberrant activation on this task has been consistently reported in patients with schizophrenia in brain regions such as the ventral tegmental area (VTA) (Nielsen et al. 2012)and nucleus accumbens (NAcc) (Radua et al. 2015). Importantly, differences in NAcc and ventral striatal brain activity during the MID task have also been directly linked with the severity of negative symptoms (Radua et al. 2015; Juckel et al. 2006). Non-imaging assessments of cognitive function were obtained using the Brief Assessment of Cognition in Schizophrenia (BACS) tool (Keefe et al. 2004; Kirkpatrick et al. 2010). We hypothesised that TAK-041 would demonstrate a greater increase compared with placebo in BOLD signal in the VS during the MID task, and an increase over placebo in the BACS composite score.

## Participants and methods

### Participants

Volunteers with a diagnosis of schizophrenia aged 18–60 years were recruited from mental health community services in the Greater London area. The study was approved by Yorkshire & The Humber– Leeds West NHS Human Research Ethics Committee (REC reference: 17/YH/0195). Screening procedures were conducted between 2 days and 35 days prior to the first dosing and scanning session. Inclusion criteria required a diagnosis of schizophrenia as defined in the DSM-5 by the Mini International Neuropsychiatric Interview, treated with a stable dose of an antipsychotic other than clozapine for at least 2 months prior to screening. Negative symptomology was confirmed using the brief negative symptom scale (BNSS). Other screening criteria such as assessment of general psychiatric and physical suitability for the study are detailed in supplementary information.

Sixty-six participants attended screening. Of these, 23 (17 males) met full eligibility criteria and were randomized into the study (age range of participants: 21–60 years; mean age of participants: 43.8 ± 12.1 years). All study visits were conducted between 21 st December 2017 and 11th September 2019.

Two participants discontinued after receiving TAK-041 during period 1 (one after inability to complete the MRI procedure and one who was unable to attend subsequent study visits) and one participant discontinued after receiving placebo during period 1 due to failure to meet inclusion criteria (positive urine drug screen).

### Cognitive and clinical assessments

The Brief Assessment of Cognition in Schizophrenia (BACS) is a reliable and sensitive battery of tests specifically designed to assess important cognitive deficits efficiently in patients with schizophrenia, and is suited to repeated testing in a clinical trial setting (Keefe et al. 2004). The primary outcome measure from the BACS is a composite score that averages the standardized scaled scores from each of the six tests (Keefe et al. 2008), and which was used as a single endpoint for the assessment (seesupplementary information). The same researcher administered each presentation of the task for a given participant to reduce inter-rater variation, and the difference between the BACS composite score in TAK-041-treated and placebo conditions on day 14 was pre-specified as a co-primary endpoint in the trial.

The Positive and Negative Syndrome Scale (PANSS; (Kay et al. 1987)) and the Brief Negative Symptom Scale (BNSS; (Kirkpatrick et al. 2010)) were employed to assess psychiatric symptoms.

Collection of all clinical and cognitive scales was paper based, before transfer and validation using an electronic data capture system.

### Study design

The study (Clincaltrails.gov registry: NCT03319953) was a randomised, double-blind, placebo-controlled, two-way crossover design. Each participant was randomised 1:1 to one of two sequence groups that defined the order of receiving a single dose of TAK-041 and placebo. The random allocation sequence was determined using a Williams square design (SAS v9.4 PROC PLAN). The block size was 4 and the sequences were generated by an independent third party and the blind maintained as described in the protocol (see supplementary information).

**Fig. 1:**
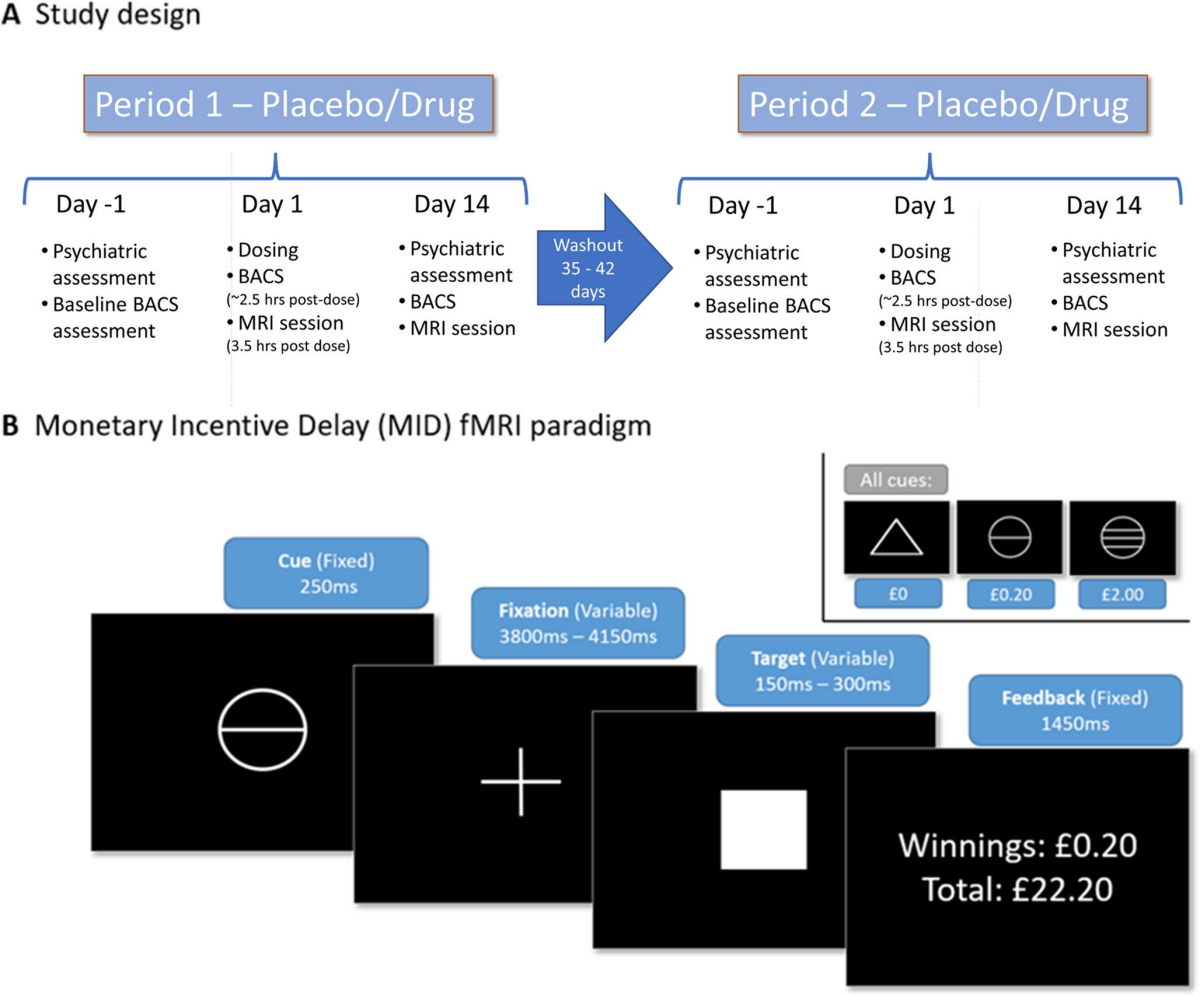
(**A**) Outline of main study design and assessments. (**B**) Schematic of monetary incentive delay task. BACS, Brief Assessment of Cognition in Schizophrenia; MRI, magnetic resonance imaging

Participants that passed screening procedures and were suitable to take part attended the National Institute for Health Research Clinical Research Facility at King’s College Hospital, London, UK. Visits were spread over two treatment periods each containing a “day − 1” baseline visit (for BACS, PANSS & BNSS baseline assessment), followed by the next day by a dosing visit (“day 1”), where the participant would receive either placebo or TAK-041 followed by completion of the BACS (2.5 h post dose) and MRI assessment (3.5 h post dose). This was followed two weeks later by a “day 14” visit (involving another BACS, BNSS, PANSS and MRI assessment). Due to the long half-life of TAK-041 observed in a previous Phase 1 study (Yin et al. 2022), a wash out period of 35–42 days separated the start of the second treatment period, where participants then repeated the baseline, day 1 and day 14 visits but received the opposite treatment that was received during period one (Fig. 1). A single follow up visit was conducted after the second treatment period for physical and psychiatric health assessments. Full details of assessments at each of these visits along with the clinical study protocol are provided in the supplementary information.

The initial dose level selected was 40 mg, which was adjusted to 160 mg after emerging pharmacokinetic (PK) and safety data from a first-in-human healthy volunteer study. This resulted in the first seven participants being randomised to receive a 40 mg dose, and the remaining 16 participants a 160 mg dose.

Blood samples for PK analysis were taken immediately prior to dosing and at 1, 2, 5 and 6 h post dose on day 1 in each treatment period, once at each day 14 visit in each treatment period and at 49 days post dose in the second treatment period only. Plasma concentrations of TAK-041 were measured by high performance liquid chromatography with tandem mass spectrometry over a concentration range of 1–1500 ng/mL. Details on the processing and analysis of these samples is available in supplementary information.

### Image acquisition and preprocessing

All MRI scans were conducted on a 3 Tesla Discovery™ MR750 scanner (GE Healthcare) using a 12-channel receive-only head coil. Structural scans acquired at screening consisted of a T2-weighted image (FOV = 240 mm, TR/TE = 4380/46.992 ms, 320 × 256 × 156 matrix, slice thickness = 2 mm) that was also used in the preprocessing of the arterial spin labelling (ASL) images, and a T1-weighted MPRAGE scan (TR/TE/TI = 7.312/3.016/400 ms, 256 × 256 × 156 matrix, voxel size 1.05 × 1.05 × 1.2 mm). Preprocessing was conducted in the Statistical Parametric Mapping (SPM) analysis suite, issue 12, on Matlab 8.2.0.701, and included resetting of image origin, slice time correction, two-pass realignment, co-registration, normalisation to MNI space using DARTEL (Diffeomorphic anatomical registration through exponentiated lie algebra (Nielsen et al. 2012)), and smoothing using an 8 mm FWHM kernel (full description in supplementary information).

#### MID task

The MID task (Knutson et al. 2000) has been used extensively to elicit and study reward-related activation within fMRI designs (Knutson and Greer 2008), and it has been shown in healthy volunteers to be reliable over time (Plichta et al. 2012). The version used in this study (see Fig. 1b) is most closely comparable to that used by Knutson et al. (Knutson et al. 2001) and has been described extensively elsewhere (Knutson et al. 2003). The MID was modelled based on previous investigations (Abler et al. 2007), and contrasts of interest were set to explore the main effect of anticipation of reward (win cue > neutral cue). Full details on the specifics of this task and its analysis can be found in the supplementary information.

The VS has been heavily implicated in reward anticipatory activity on the basis of results from previous fMRI studies as well as converging electrophysiological and anatomical evidence. Therefore, left and right ventral striatal regions of interest (ROIs) were defined as previously described (Montgomery et al. 2006; Mawlawi et al. 2001). The mean beta estimates for the reward-anticipation contrast created from first-level modelling were extracted from each ROI using the MarsBar plugin in SPM. The mean of left and right beta estimates was predefined as the primary outcome measure for this task, and the difference between TAK-041-treated and placebo conditions on day 1 was predefined as a co-primary endpoint for the trial.

Analysis of the MID task behavioural data was conducted to assess the effect of TAK-041 on task engagement and performance and are fully described in the supplementary information, along with exploratory correlations between extracted beta values with the PANSS negative subscales scores and BNSS domains, and with the composite score on the BACS.

#### Arterial Spin Labelling

Whole-brain maps of regional CBF were obtained using an ASL methodology previously reported (Hawkins et al. 2018; Dai et al. 2008) and are fully described in the supplementary information.

Mean CBF values across voxels within the following regions were predefined as exploratory endpoints for the study: VS, caudate, putamen, thalamus, anterior cingulate and middle frontal gyrus. Statistical analyses were performed on the bilateral averages of the left and right values for each region.

### Statistical methods and endpoints

The MID and BACS primary endpoints were analysed using a Bayesian normal linear model with effects for sequence, period, treatment, time (as a categorical variable), the treatment-by-time interaction, subject-within-treatment sequence, and baseline (for BACS only)(Carlin et al. 2009; Chen 2009). For BACS, the observed value and the change from baseline were modelled separately.

The study was powered according to the following Bayesian criteria. The criterion for a positive result for the BACS was at least 70% posterior probability of a difference between TAK-041 and placebo greater than two points in the BACS composite score at day 14. The criterion for a positive for the MID was at least 70% posterior probability of a difference between TAK-041 and placebo of greater than 0.09 in the average of left and right VS activation in the MID fMRI at 3.5 h post dose on day 1. The predefined positivity criterion for the MID task was informed by previously acquired data with the same task at the same imaging centre.

To assess any potential effect of drug induced changes in CBF on the BOLD signal, the MID ventral striatal endpoints were analysed at Day 1 and Day 14 separately using a linear mixed effects model with effects for treatment, ventral striatal CBF, the treatment-by-CBF interaction, and random effect of subject.

### Exploratory endpoint analysis

A linear mixed effects model for repeated measures was fitted to each of the exploratory pharmacodynamic measures including PANSS, BNSS and ASL endpoints.

### Exploratory voxelwise analysis of whole-brain images

Additional analyses were performed on the whole-brain imaging dataset to examine the effect of TAK-041. For the MID task, delta images of the reward-anticipation contrast maps were created from the placebo versus TAK-041 session for each participant at each time point (e.g. placebo Day 1 vs. TAK041 Day1 (herein referred to as (∆Day1) and placebo Day 14 vs. TAK041 Day 14 (herein referred to as (∆Day14)). Voxelwise one-sample *t*-tests were then conducted at each time point separately (∆Day1 and ∆Day14), with recorded plasma blood concentration values from samples taken proximal to the MRI session included as covariates. The threshold for group comparisons was set at a significance level of *p* < 0.001 uncorrected for whole-brain maps, and at *p* < 0.05 FWE-corrected (small volume correction [SVC]) within the ventral striatal ROI.

The whole-brain exploratory analysis was conducted with data from those participants who completed both a drug scan and a placebo scan at a given timepoint, which allowed for the calculation of the delta image required for the image analysis software employed. After data QC and task performance checks, twenty participants had MID ∆Day1 data and seventeen participants had MID ∆Day14 data (see supplementary information for full details on data exclusions).

∆Day1 and ∆Day14 images were also created for each placebo–drug ASL pair of images prior to smoothing, and the data analysed as above. Twenty participants had ∆Day1 CBF maps and seventeen participants had ∆Day14 CBF maps available for whole brain perfusion analysis– three day 14 scans were excluded due to incomplete acquisition, head movement and coregistration failure during preprocessing.

To assess the potential effect of drug induced changes in resting cerebral blood flow on the voxelwise MID elicited BOLD signal, the MID analysis of the ∆Day1 and ∆Day14 datasets was repeated with the ASL delta images included as voxelwise covariates in the model.

## Results

### Participant characteristics

Demographic and baseline clinical characteristics of the participants are summarised in Table 1. The PANSS and BNSS were evaluated at baseline (day − 1) and day 14 in both periods; no statistically significant differences between days − 1 and 14, or between TAK-041- and placebo-treated conditions were detected (the maximum difference was observed on the positive subscale of the PANSS at Day 14 between placebo (14.81 (4.83)) and TAK-041 160 mg (14.36 (4.57)) (Supplementary Table S1).

**Table 1:** Baseline characteristics

Characteristic	Dose group		
	40 mg	160 mg	All participants
Number of participants	7	16	23
Age (years)	40.4 (14.9)	45.3 (10.8)	43.8 (12.1)
Female sex, n (%)	2 (29)	4 (25)	6 (26)
Race (n)			
Asian	0	1	1
Black or African American^a^	6	10	16
Multiple^b^	0	1	1
White	1	4	5
Weight (kg)	85.1 (18.9)	93.9 (22.6)	91.6 (21.4)
Height (cm)	174.0 (10.0)	172.6 (8.5)	173.0 (8.8)
Body mass index	28.1 (4.0)	31.3 (6.1)	30.3 (5.6)
BACS composite score	27.4 (12.8)	27.9 (8.2)	27.72 (9.6)
PANSS negative symptoms, total	21.6 (4.2)	19.9 (3.3)	20.4 (3.6)
PANSS positive symptoms, total	18.7 (5.3)	18.9 (6.4)	18.9 (5.9)
PANSS overall, total	67.1 (11.0)	65.9 (12.7)	66.3 (11.9)

^a^‘Black or African American’, ‘Black British’ and ‘Black British Caribbean’ were combined into one category

^b^Multiple: ‘White’ + ‘Black or African American’

BACS, Brief Assessment of Cognition in Schizophrenia; PANSS, Positive and Negative Syndrome Scale

### TAK-041 Pharmacokinetics

**Fig. 2:**
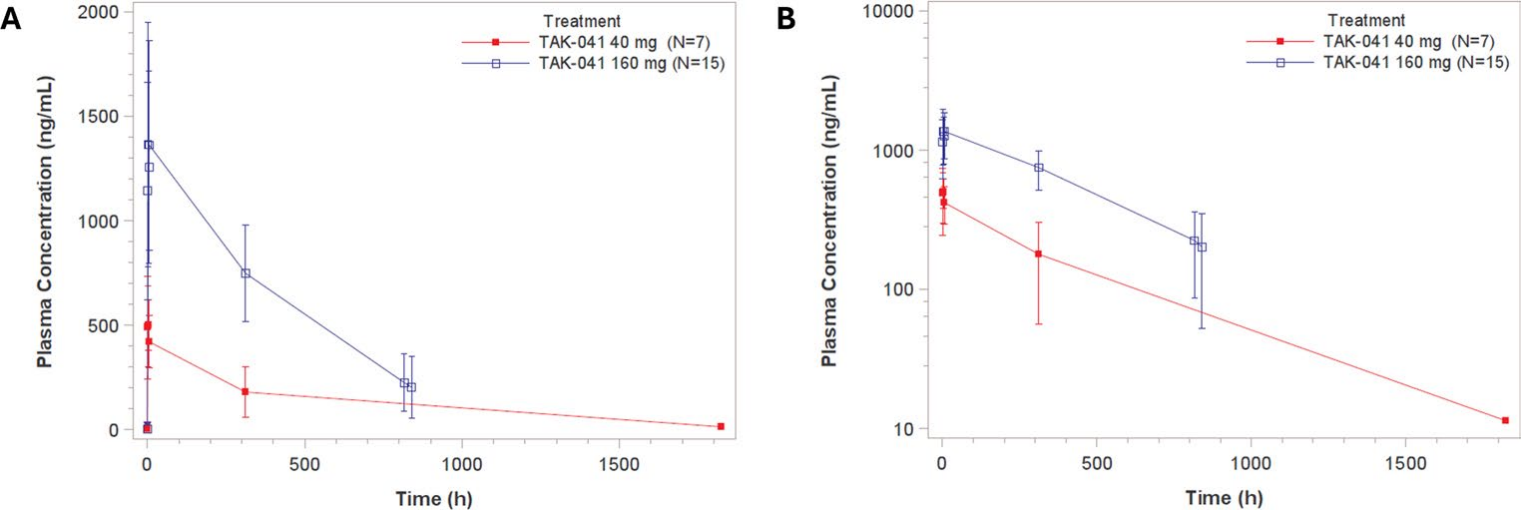
Plasma concentration (ng/mL) post dose for 40 mg and 160 mg single-dose TAK-041 on (**A**) linear and (**B**) log-scale y-axes

The PK data indicate that a single dose of TAK-041 was rapidly absorbed with a median t_max_ of 1.83 h and 1.92 h for the 40 mg and 160 mg dose levels, respectively. A dose-dependent increase in TAK-041 plasma exposure (C_max_ and area under the curve [AUC]) was observed (Fig. 2). Mean C_max_ values increased 2.8-fold (549 ng/mL and 1523ng/mL for 40 mg and 160 mg doses, respectively), and mean AUC_∞_ values increased 3.5-fold (217 h∙µg/mL and 763 h∙µg/mL for 40 mg and 160 mg doses, respectively) for a 4-fold increase in TAK-041 dose. Moderate inter-subject variability was observed for plasma exposure parameters C_max_ and AUC_∞_(range: 30.1–62.7%). The mean half-life for TAK-041 was 278 h and 349 h for TAK-041 at 40 mg and 160 mg, respectively, and at day 14 (or 336 h) blood plasma levels of TAK-041 were still present. These observations are consistent with those in previous studies with TAK-041 in humans (Yin et al. 2022).

### BACS

Twenty-two participants had at least one baseline (day − 1) and day 1 BACS assessment, while there were 20 participant data sets available for day 14 (Supplementary Table S2). The posterior mean (SD) values for the difference between the BACS composite score in placebo and TAK-041 dose levels 40 mg, 160 mg and all participants combined on day 14 were − 0.23 (2.95), −0.84 (1.87) and − 0.61 (1.50), respectively. The posterior probability that the population increase over placebo in the BACS composite score at day 14 was greater than 2 points was 0.2079, 0.0633 and 0.0413, respectively. These results did not meet the predefined positivity criterion for the BACS.

### MID fMRI

**Fig. 3:**
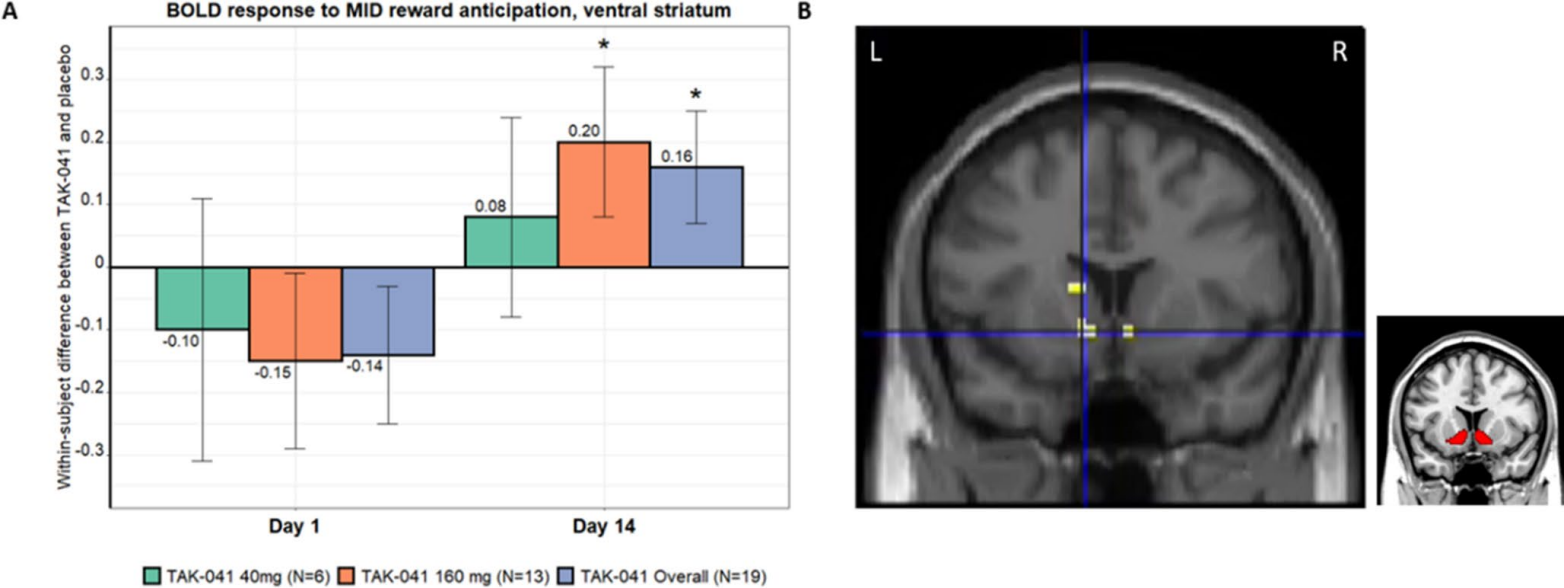
(**A**) Within-subject differences between TAK-041 and placebo in activation of the VS during reward anticipation in the MID task. The individual-level data used for this model were the average overall voxels within a predefined region of interest comprising both left and right VS. Results are shown as mean ± SD by visit, for each dose group independently and overall (both dose groups combined); *indicates that the statistical success criterion was met (the posterior probability that the increase over placebo is greater than 0.09 was at least 70%). (**B**) Voxelwise TAK-041 > placebo contrast in the VS at day 14 for the reward anticipation condition. Cross hairs indicate peak of small volume correction for left VS (*n* = 17, *p* = 0.038 FWE corrected; cross hair coordinates (− 10 13 − 7) mm in MNI space). Inset: Location of ventral striatal ROIs. BOLD, blood oxygen level dependent; FWE, familywise error; MID, monetary incentive delay; MNI, Montreal Neurological Institute; SD, standard deviation; VS, ventral striatum

After imaging and task quality control checks (see supplementary information) there were 20 participants with at least one day 1 scan and 19 participants with at least one day 14 scan from which VS parameter estimates for the reward-anticipation contrast were extracted and entered into the Bayesian analysis. Figure 3a presents modelled group differences between the ventral striatal activation under TAK-041 and placebo on days 1 and 14.

On day 1, there was a decrease in ventral striatal activation with TAK-041 relative to placebo. The posterior mean (SD) values for the difference in fMRI BOLD endpoint in placebo and in TAK-041 dose levels 40 mg, 160 mg and all participants combined on day 1 were − 0.10 (0.21), − 0.15 (0.14) and − 0.14 (0.11), respectively. The posterior probabilities that the increase over placebo in the fMRI BOLD endpoint at day 1 is greater than 0.09 were 0.1706, 0.0373 and 0.0209, respectively. These results did not meet the predefined positivity criterion for the MID fMRI task.

However, on day 14 there was a dose-related increase in activation with TAK-041 relative to placebo that met the predefined statistical criterion for the 160 mg dose and for all participants combined. The posterior mean (SD) values for the difference in placebo and in TAK-041 dose levels 40 mg, 160 mg and overall on day 14 were 0.08 (0.16), 0.20 (0.12) and 0.16 (0.09), respectively. The posterior probability that the treatment difference on BOLD MID fMRI for these dose levels is greater than 0.09 was 0.4657, 0.8314 and 0.7913, respectively.

Linear mixed effects models of the effect of TAK-041 on reward anticipation including CBF as a covariate at Day 1 revealed no main effect of treatment, CBF or treatment*CBF interaction, collapsed across dose levels. Including dose level in the model did not reveal any significant effects or interactions.

The same analysis at Day 14 revealed a main effect of treatment (F(1,46) = 7.08, *p* = 0.011), no main effect of CBF and a treatment*CBF interaction (F(1,46) = 5.84, *p* = 0.020, Supplementary Table S4). Pairwise comparisons indicated that TAK-041 significantly increased reward anticipation BOLD at Day 14 compared to placebo (an increase of 0.101, *p* = 0.020)) as seen in the Bayesian analysis, equivalent to an effect size of d = 0.567. Including dose level in the model did not reveal any significant effects or interactions.

### Exploratory imaging analysis

#### MID voxelwise analysis

A one-sample *t*-test of ∆Day1 images of placebo vs. TAK-041 scans analysis did not reveal any significant changes either at whole-brain or SVC level. However, the same analysis of the ∆Day14 scans revealed an SVC-corrected increase in the right ventral striatal ROI in TAK-041 compared with placebo (peak at [7 10 − 7] mm; t = 3.23, *p* = 0.031 FWE corrected, d = 0.677). The left ventral striatal ROI also contained a significant peak ([− 10 13 − 7] mm; t = 3.28, *p* = 0.038 FWE corrected, d = 0.811) (Fig. 3b).

Including the ∆Day14 CBF images as a voxelwise covariate in the same model resulted in the peaks in the right (t = 2.611, *p* = 0.387, d = 0.650) and left ventral striatum (t = 3.545, *p* = 0.064, d = 0.908) no longer meeting statistical significance. However, this additional analysis was conducted on a reduced sample of the *n* = 14 participants who had full imaging datasets for both the MID and ASL available on each treatment arm.

Additional analyses assessing effect of dose level and relationship of extracted beta values with clinical scales did not reveal significant results and are reported in the supplementary information.

#### ASL CBF

##### **ROI analysis**

**Fig. 4:**
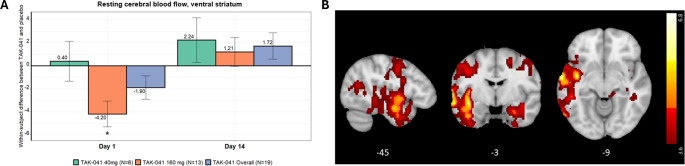
(**A**) Modelled within-subject differences between TAK-041 and placebo in ventral striatal CBF, d = 0.95 at Day 1, 160 mg. (**B**) CBF map of TAK-041 < placebo at day 1 (*n* = 20; significant clusters only at *p* = 0.05 FWE cluster-level correction [peak level *p* = 0.001 uncorrected], including covariates for blood plasma concentration)). Within the most prominent cluster (k = 9516) localised to the left temporal lobe, there were peaks centred around the temporooccipital and anterior parts of the left middle temporal gyrus([−60 − 62 3] mm, t = 6.74; [−64, −7, −9], t = 6.10), and left insula cortex ([−41, −7, −18], t = 5.97). There were additional significant clusters around the right parahippocampal gyrus and hippocampus, and right superior and middle frontal gyrus

The ROI analysis revealed similar trends across all ROIs, with predominant decreases in CBF on TAK-041 compared with placebo on day 1, and predominant increases in CBF on TAK-041 compared with placebo on day 14 (Fig. 4a and Supplemental Fig. S1). At day 1, the decrease at 160 mg was greater than at 40 mg and achieved nominal statistical significance (*p* < 0.05) in all ROIs. At day 14, the mean difference from placebo was numerically greater at 40 mg than 160 mg, but neither were nominally statistically significant.

##### **Voxelwise analysis**

A one-sample *t*-test of ∆Day1CBF images (*p* = 0.05 FWE cluster-level correction, peak level *p* = 0.001 uncorrected) revealed widespread and unidirectional grey matter reductions in CBF after a single dose of TAK-041 at day 1, comprising of two large clusters with peaks around the left middle temporal gyrus/lateral occipital cortex, left insula and right hippocampus (Fig. 4b). The same analysis of the ∆Day14 scans did not reveal any changes that survived whole brain correction.

## Discussion

Using a single dose of the novel compound TAK-041, we did not find evidence for improvement in BACS performance, and performance was not correlated with any of the changes seen in the extracted BOLD values. This may be because cognition is not affected by single-dose TAK-041 in patients despite promising results from preclinical models, or the study may not be adequately powered to detect this effect. However, there are no clear examples of drugs to improve cognition translating from experimental animals to patients (Goetghebeur and Swartz 2016). This does not preclude the potential for an improvement of specific domains of function within subgroups of this heterogenous illness with chronic dosing (Waltz 2017), but we were unable to test that hypothesis in this study.

The primary hypothesis of an increase in reward-anticipation signal after TAK-041 compared with placebo was not met at day 1 in the Bayesian analysis, although it was achieved at day 14. In the whole-brain analysis a similar trend was seen, with no observable changes at day 1 (3.5 h after dosing), while there was a significant increase in VS activation observed at day 14. Other studies in schizophrenia cohorts have indicated that when unmedicated patients are given a course of antipsychotic treatment, the ventral striatal response to reward anticipation that was previously absent (compared with healthy controls) is recovered (Nielsen et al. 2012). A similar trend has also been reported in patients treated with second-generation antipsychotics (SGAs) compared with first-generation antipsychotics (FGAs) (Juckel et al 2006; Kirsch et al. 2007), with the same trend seen in patients who were switched from an FGA to an SGA (Schlagenhauf et al. 2008). Here, we show that the effects of TAK-041 are detectable after 2 weeks when a single dose is added to existing medication. These changes in striatal response have been linked to changes in both positive (Nielsen et al. 2012) and negative (Radua et al. 2015; Juckel et al. 2006) symptoms. Explanations of this effect have centred around the potential correction of a dysfunctional DA basal state, meaning that the previously ‘drowned out’ phasic response to reward anticipation can then be detected (Heinz and Schlagenhauf 2010), resulting in preserved reward function and reduction in negative symptoms. Here, we show the potential for a similar effect using a drug with a distinct mechanism of action compared with current typical or atypical antipsychotic medications, when given as an add-on to existing treatment.

An exploratory analysis of the changes in symptomatology as measured on the PANSS and BNSS did not reveal any relationship between reward-anticipation response and severity of symptoms in the current study, apart from a negative association between anhedonia and ventral striatal response at day 14. Even though this was an exploratory analysis we note this did not survive multiple-comparisons correction. However, the studies that have reported associations between symptom change and ventral striatal responses typically assess patients over a longer time scale (e.g., 6 weeks). A longer dosing period may be required to understand the impact of these imaging changes on symptoms and behaviour.

The ASL results indicate that multiple brain regions may be involved in the drug effects. Current antipsychotic medication has consistently been shown to increase CBF, typically to areas with a higher density of D2 receptors, such as the striatum (Hawkins et al. 2018; Selvaggi et al. 2019; Handley et al. 2013). Here, TAK-041 produced a widespread decrease in CBF throughout the cortex and subcortex on day 1. This may be due to direct action of the compound on local GPR139 receptors in the grey matter, or it may be due to a downstream effect of agonism in areas with a high density of GPR139 receptors such as the habenula. Either way, the acute physiological response is markedly different to that of typical or atypical antipsychotics.

For both the MID task and the CBF, different effects of TAK-041 were seen 2 weeks after dosing compared with 3.5 h post-dosing. It is unlikely this is merely due to the different levels of exposure at the two time points because the PK data show that participants who received a 40 mg dose had mean blood peak plasma concentrations at day 1 (549 ng/mL) that were about 73% of the day 14 levels of participants who received the 160 mg dose (747 ng/mL). For both the MID task and the CBF, the brain changes between day 1 and day 14 were also in opposition (an exposure model would instead predict a clear change on day 1 that was attenuated at day 14). The long plasma half-life of this compound (278–349 h), compared with the half-lives of existing antipsychotics such as olanzapine (33 h), risperidone (20 h) and amisulpride (12 h), means the continued presence of the drug over 2 weeks may allow more sustained modulation of relevant brain systems. For the MID task, the increased activity during reward anticipation on day 14 could plausibly be the result of the 2 weeks of drug activity allowing the DA levels in the striatum to return to a level whereby the phasic signal produced by reward anticipation can be detected. In order to provide supporting evidence for this mechanism in vivo, PET scans with [^18^F]DOPA to index pre-synaptic DA or a DA-release paradigm would be required. In healthy volunteers, amphetamine-induced DA release is sensitive to TAK-041 and was attenuated at rest in the acute stage (Rabiner et al. 2021). How this changes after the continued exposure over 2 weeks in patients is not known.

There are limitations to this study. The PK data indicates that participants who received TAK-041 during their first period still had residual levels of the compound in their blood when commencing their placebo arm (albeit at vastly reduced levels to those present in the drug arm across the cohort as a whole), suggesting the 35–42 day washout was an insufficient amount of time to allow full clearance (see Fig. 2). Further investigations should take into account the PK data from more recent research with this compound during study design. There is a loss of analytical power from the split in dose sizes across the cohorts, and interpreting the difference between these dose levels is limited by the small number of patients who received the initial smaller dose. The decision to increase the dose was carefully made based on emerging PK data in ongoing human and animal studies to increase the potential to detect a drug effect. Individuals in this cohort inevitably have differing symptomatology and medication profiles and therefore it is not possible to determine from this study if TAK-041 has differential responses in those with differing clinical profiles– however, it should be noted it is also an ecologically valid cohort of community patients with negative symptoms and it is of critical importance to assess these compounds in ‘real-life’ patients. In terms of the imaging assessments, there is lack of clarity in how closely the ventral striatal response reflects positive or negative symptoms– an important issue given the apparent clear independence of these two domain dimensions (Lindenmayer et al. 1995; Wallwork et al. 2012). However, the extensive CBF changes observed after the single dose could indicate wider-ranging effects of this compound on additional brain systems beyond the subcortical reward system. This, along with the evidence in animal models of the effectiveness of the compound in recovering ‘negative symptoms’ suggests that additional studies with a wider range of fMRI paradigms assessing negative symptomology (Goghari et al. 2010) subserved by additional brain networks would give a fuller understanding of the potential effects of this compound.

It should also be considered that the CBF changes seen at both Day 1 and Day 14 may have influenced the BOLD response to reward anticipation. An exploratory analysis including the CBF as a voxelwise covariate of no interest reduced the significance of the drug effect on reward anticipation in the ventral striatum in the whole brain model, although this analysis did require reducing the sample size to *n*= 14 due to several participants not having both MID and ASL data available, resulting in an associated loss of power. Including CBF in the linear mixed model (which can deal with missing values) to assess the effect of TAK-041 on reward anticipation confirmed a main effect of treatment on reward anticipation but did not reveal a main effect of CBF. The interaction between CBF*Treatment does not have a straightforward interpretation, but the apparent more pronounced effect of the drug on reward anticipation at lower baseline levels of CBF may be related to the fact that the BOLD amplitude has been shown to be decreased when baseline CBF is increased (Lu et al. 2010; Simon and Buxton 2015). This may reflect the reduced difference between TAK-041 and placebo at higher levels of CBF (although there are several other plausible interpretations of this interaction). Regardless, the fact the main effect of drug persists after covarying for CBF does indicate that possible drug induced changes in CBF are not solely responsible for the change in reward anticipatory BOLD observed at Day 14, by which point the perfusion changes were reduced in magnitude compared to the changes observed acutely. Previous research has also indicated drug induced changes to reward anticipatory BOLD response are robust to concurrent drug induced changes on the vasculature (Hawkins et al. 2021). An exploratory analysis of the drug effect on the outcome phase (contrast win feedback > no win feedback) revealed no drug effect at ∆Day1 or ∆Day14 at whole brain or at SVC level in the ventral striatum ROI, which indicates the effect observed is specific to the reward anticipatory component of the task.

In conclusion, we were able to demonstrate that the GPR139 agonist TAK-041 produces distinct effects on brain activity at day 1 and day 14 after administration of a single dose to patients with schizophrenia. CBF decreased on day 1 in widespread cortical regions while reward-related ventral striatal activity increased two weeks post-dose, emphasizing the potential effects of TAK-041 after continued exposure due to the long half-life of the compound. These changes in reward-anticipation-related brain activity and CBF occurred in patients stably treated with antipsychotic medication and in brain regions consistently linked with both symptoms and treatment response in schizophrenia. Interactions observed between drug condition and CBF indicate drug induced changes on cerebral perfusion may partially contribute to the changes observed in BOLD, although these changes to reward anticipatory activity remained significant after the inclusion of CBF in the model. Additional research (such as utilisation of other imaging modalities and behavioural paradigms as outlined above) is required to further elucidate the mechanism of action of this compound.

## Supplementary Information

Below is the link to the electronic supplementary material.

Supplementary Material 1

Supplementary Material 2

Supplementary Material 3

## Data Availability

This was a commercial trial; data is not available.
