# Supplementary material for: The GPR139 agonist TAK-041 produces time-dependent alterations to cerebral blood flow and reward system function in patients with schizophrenia: a randomised placebo-controlled trial
Source: Psychopharmacology (Berl). 2025 Aug 16;243(4):819–29. doi: 10.1007/s00213-025-06884-x (PMC13035629; doi:10.1007/s00213-025-06884-x)
Supplement: Supplementary file 1 — Supplementary Material 1 [file 213_2025_6884_MOESM1_ESM.docx]

# Supplementary Information

## Participants and Methods

Inclusion criteria required a diagnosis of schizophrenia as defined in the Diagnostic and Statistical Manual of Mental Disorders, Fifth Edition (DSM-5) by the Mini International Neuropsychiatric Interview, without a current and active diagnosis of a significant psychiatric illness (e.g., bipolar disorder, depression or generalised anxiety disorder) other than schizophrenia, as per DSM-5 (including meeting criteria for substance-use disorder or history of alcohol abuse within the month prior to screening). Participants must have been treated with a stable dose of an antipsychotic other than clozapine for at least 2 months prior to screening, and score ≤ 90 on the Positive and Negative Syndrome Scale (PANSS). To ensure the presence of negative symptomology, a negative symptoms factor score (NSFS) of ≥ 15 was required on the PANSS at screening (N1 Blunted Affect, N2 Emotional Withdrawal, N3 Poor Rapport, N4 Passive Social Withdrawal, N6 Lack of Spontaneity and Flow of Conversation, G7 Motor Retardation, and G16 Active Social Avoidance). To ensure stability of symptoms, the PANSS was readministered on the day prior to randomization and participants with NSFS or total PANSS scores that changed more than 20% between screening and day −1 were excluded. Participants who were deemed at risk of suicide during the screening window according to the Columbia Suicide Severity Rating Scale were also excluded. General physical suitability for the study was assessed from medical history, concomitant medication and results of a full physical exam, a vital signs check, electrocardiography, blood and urine chemistry profiles, serology (HIV1, HIV2, hepatitis B and/or hepatitis C and pregnancy) and screening for alcohol and drugs of abuse. Only participants judged to be in good physical health by the study medic were included. Exclusion criteria included history of cancer, values greater than upper limit of normal on liver function tests or a positive drug of abuse or alcohol breath test on the screening or study days. Participants also had to agree to adhere to contraception guidelines while taking part to avoid pregnancy during the study.

An MRI scanning visit within the screening window was conducted to familiarise the participant with the scanning session. T1- and T2-weighted images were acquired and inspected by a neuroradiologist to rule out any structural brain abnormalities or other abnormalities that would interfere with interpretation of functional brain imaging results.

## Study design

The study was a randomised, double-blind, placebo-controlled, two-way crossover design. Each participant was randomised to two sequence groups that defined the order of receiving a single dose of TAK-041 and placebo. Prior first-in-human studies indicated that TAK-041 is rapidly absorbed (median t_max_ of 1–2 hours) with well-characterised plasma concentration–time profiles, but it exhibits a long plasma half-life of approximately 11 days and a duration of detectable plasma exposure of approximately 40 days [1]. A 35–42-day washout interval was thus incorporated between the two treatment periods in the current study (Fig. 1a). Visits were as follows:

- Baseline assessments visit (day −1 in each treatment period): final screening assessments (blood tests, urinalysis, and a drug screen) were completed as well as cognitive baseline practice and testing, baseline psychiatric scales and familiarization with scanner tasks.
- Dosing and first testing visit (day 1 in each treatment period): following a physical health check by a study physician (drug and alcohol screen, ECG, vital signs check and physical exam), a single dose of either TAK-041 or placebo was administered between 10.30 am and 12 pm. The BACS was administered approximately 2.5 hours post dose, and the fMRI session began 3.5 hours post dose.
- Second testing visit (day 14 in each treatment period): 2 weeks after dosing day, participants returned to complete the BACS and another fMRI session, identical to the initial session. These were conducted at the same time of day as the day 1 testing sessions.
- End of period 1 visit (days 35–42) and start of period 2: a single end of period 1/start of period 2 visit acted as the baseline assessment visit (day −1) for period 2, after which day 1 and day 14 visits were repeated as above with the participants receiving the opposite treatment to that received in period 1.
- Follow-up visit (day 49 [± 4 days] post period 2 dose): a safety follow-up involving physical and psychiatric health assessments.

Dosing involved participants drinking a single bottle of oral suspension, containing either TAK-041 (crystalline TAK-041 40 mg or 160 mg mixed with 70 mL Tween/Methyl cellulose (MC) vehicle) or placebo (70 mL of Tween/MC vehicle). Participants were instructed to drink the full contents of the bottle. The bottle was then rinsed twice with 35 mL of water; the participants also drank this water.

The sponsor statistician generated the allocation code which determined the order in which participants would receive TAK041 and placebo. When randomised into the study, participants were assigned a sequential randomisation number from this code. A study pharmacist who was provided with the randomisation code prepared the treatment order of the medication accordingly and dispensed to the study researcher in a sealed and wrapped bottle marked with the randomisation number of the participant at the start of day 1 of each treatment period. All participants and members of the site research team remained blinded throughout the duration of the study, apart from the study pharmacist.

## Image acquisition and preprocessing

All MRI scans were conducted on a 3 Tesla Discovery™ MR750 scanner (GE Healthcare) using a 12-channel receive-only head coil. Structural scans acquired at screening consisted of a T2-weighted image (FOV = 240 mm, TR/TE = 4380/46.992 ms, 320 × 256 × 156 matrix, slice thickness = 2 mm) that was also used in the preprocessing of the arterial spin labelling (ASL) images, and a T1‐weighted MPRAGE scan (TR/TE/TI = 7.312/3.016/400 ms, 256 × 256 × 156 matrix, voxel size 1.05 x 1.05 x 1.2 mm), also used in diffeomorphic image registration through exponential algebra (DARTEL) normalization. Functional scans acquired at day 1 and day 14 (MID) were carried out using a temporal series of gradient-recalled echo-planar imaging whole-brain scans, each comprising 39 near-axial slices, with an isotropic spatial resolution of 3.3 mm (TR = 2000 ms; TE = 28 ms; flip angle = 75 degrees; total number of volumes in the time series = 336; FOV = 214 mm). The initial four volumes of each time series were discarded in order to minimise non-steady-state effects on the signal amplitude.

All preprocessing was conducted using Statistical Parametric Mapping (SPM) analysis suite software, version 12 (Functional Imaging Laboratory, UCL, London, UK), running on Matlab 8.6 (MathWorks, Natick, MA, USA) unless stated otherwise. The origins of the functional and structural images were reset to the anterior commissure-posterior commissure line. Functional images were corrected for slice time (reference slice: 19). An initial between-session alignment was performed for each participant in which the first volume from sessions two, three and four were aligned to the first volume of the first session prior to two-pass realignment within each session. All volumes were then realigned to the mean image of all four sessions. The T1-weighted image for each subject was then co-registered to the resampled mean functional image from the realignment step, using the normalised mutual information objective function in SPM. DARTEL [2]) was used to create a cohort template from the T1-weighted images. The realigned functional volumes were resliced to original voxel sizes and the DARTEL flow fields were applied to warp the data into MNI space. Normalised images were smoothed using an 8-mm FWHM kernel. Scans were conducted 3.5 hours post dose on day 1 and at the same time of day at each subsequent visit.

## fMRI paradigm: MID task

The MID task involves the display of a cue which the participant has previously learnt to associate with a certain level of monetary reward. To win the reward, the participant must press a button as quickly as possible in response to the brief appearance of a white square, which appears a variable amount of time after the cue. An outcome display then reveals if the reward was won or not, depending on if the participant pressed the button within the response window. The reaction time window adapts to the performance of each participant with a pre-set target of a 66% win-rate to ensure an adequate number of win and lose outcome trials for subsequent analysis.

The version used in this study comprises of three randomised trial types, comprising of active reward-level cues (high (£2), low (£0.20) and control (£0)). If the participant pressed the button during the presentation of the fixation cross or within 100ms of the presentation of the target (an unrealistic reaction time), the trial would be set as a no win.

Each of the three active trial types were conducted within a fixed 9.25 second window. Each condition was presented 24 times for a total of 72 trials over a total duration of approximately 11 minutes.

### First-level model

The MID was modelled as originally outlined in [3], using boxcar regressors convolved with a haemodynamic response function. Three cue regressors (high win [HCue], low win [LCue] and neutral [NCue]) were defined for the anticipatory period depending on the cue presented (as a result of temporal jittering, this period had a variable total amount of time between 4050 ms and 4400 ms, depending on the trial). The target was defined by a single regressor of 500 ms. Five feedback regressors (high win [H+Win], low win [L+Win], high no win [H-Win], low no win [L-Win] and neutral feedback) were defined for the feedback period depending on the cue type and outcome (win or no win). These regressors were set for a fixed period (1450 ms). Motion parameters estimated during the realignment process were added as regressors in the first-level design matrix, in addition to framewise displacement [4]. Any volumes with displacement of 1 mm or more were flagged and suppressed using a 3-TR regressor (to include the volumes either side) in the first-level design matrix [5]. Any scan that required more than 10% of the volumes of the full run being regressed out in this manner resulted in that participant being removed from the analysis. The realignment parameters were visually inspected, and any time series for which the maximum detected translation from the first volume was greater than the dimensions of one voxel, or those that indicated stimulus-correlated movement, were flagged for exclusion.

Performance-related criteria were also set to ensure that only data from participants who were actively and appropriately engaged in the task were included in the final analysis. When participants failed to make a response to an active task (i.e., no button press was attempted), the entire trial was defined as a missed response regressor of no interest. Any trials with a reaction time (RT) of ±3 standard deviations (SDs) of that individual's mean were also classified as a missed response. This was to ensure that only trials that the individual was actively participating in were included in the analysis. If more than 10% of a single active trial type was regressed out after applying this threshold, that subject was removed from the analysis.

Contrasts of interest were set to explore the main effect of anticipation of reward (HCue and LCue > NCue). Levels of reward were combined here to ensure that a sufficient number of trials were obtained for a suitably powered analysis. An additional contrast to explore the feedback phase of the task was also set to assess the specificity of any drug effect on the anticipatory phase (All win trials > All no win trials).

### Behavioural analysis

All behavioural analyses were conducted in SPSS Statistics software, version 26 (IBM, Portsmouth, UK). Response rate (percentage of trials requiring a button press that a participant responded to) was used as a measure of task engagement and analysed with a repeated-measures analysis of variance (ANOVA), with drug (TAK-041 or placebo) and time (day 1 and day 14) as within-subject factors. A second analysis included dose (40 mg or 160 mg) as a between-subject factor.

Hit rate (percentage of trials that a participant successfully responded to within the RT window) was separately analysed at each time point with a repeated-measures ANOVA, with drug (TAK-041 or placebo) and reward level (low or high) as within-subject factors, and dose level (40 mg or 160 mg) as a between-subject level factor.

Average reaction time was also analysed at each time point with a repeated-measures ANOVA, with drug (TAK-041 or placebo) and reward (neutral, low or high) as within-subject factors and dose level (40 mg or 160 mg) as a between-subject level factor.

## Arterial Spin Labelling (ASL)

Images were acquired using a pseudo-continuous ASL sequence with a multi-shot, segmented 3D stack of axial spirals (8 arms) readout with a resultant spatial resolution of 2 x 2 x 3 mm. Three control-label pairs were used to derive a perfusion-weighted difference image. The labelling RF pulse had a duration of 1.5 s and a post-labelling delay of 1.5 s. The sequence included background suppression for optimum reduction of the static tissue signal. A proton density (PD) image was acquired in 48 s using the same acquisition parameters to compute the CBF map in standard physiological units (mL blood/100 mg tissue/min). Two runs were acquired per visit and averaged after preprocessing.

The T2-weighted image was co-registered to the T1-weighted image prior to the creation of the DARTEL template, and the PD image from each session was then co-registered to the T2-weighted image. The parameters for this transformation were then applied to the CBF maps, and the DARTEL flow field was applied to normalise the CBF maps to MNI space, before smoothing with a 6 mm FWHM kernel.

## BACS

The BACS tool is a reliable and sensitive battery of tests specifically designed to assess important cognitive deficits efficiently in patients with schizophrenia, and is suited for repeated testing in a clinical trial setting [6]. It has shown superior internal consistency and test–retest reliability to alternative tests of neuropsychological status [7]. The BACS tool assesses six domains of cognitive function found to be impaired in schizophrenia: verbal memory, working memory, motor function, attention and processing speed, verbal fluency and executive function. Further details of these tasks are provided in the Supplementary Information.

The following domains of cognitive function found to be consistently impaired in schizophrenia were assessed in this study:

Verbal Memory: Participants were presented with 15 words and asked to recall as many as possible. This procedure is repeated five times and gives a score of verbal recall in total number of words accurately recalled. Four different lists of words were counterbalanced between participants to protect against learning effects.

Working Memory: The Digit Sequencing task involved participants being auditorily presented with a sequence of numbers (e.g. 936) of increasing length per trial, which they were required to repeat back to the experimenter, in order from lowest to highest. Four sets of numbers were given at each sequence length, from two up to eight. The task was discontinued if all sets at sequence level were missed. This gave a score of total number of correct sets recalled.

Motor Function: 100 plastic tokens were placed on a flat surface in front of the seated participant and divided evenly either side of a container. Participants were asked to pick up one token with each hand from opposite sides of the container at the same time and place them into the container at the same time as quickly as possible for 60 seconds. This gave a score of total number of tokens correctly placed into the container.

Attention and Processing Speed: Participants were shown a key linking a list of nine unique symbols to the individual numerals 1-9, and were asked to fill match in the corresponding number beneath to a series of symbols as quickly as possible for 90 seconds, giving a score of number of correct items.

Verbal Fluency:

1) Semantic fluency: Participants were given 60 seconds to name as many words as possible within a given category (animal names), giving a score of number of correct words.

2) Letter fluency: Participants were given 60 seconds to generate as many words as possible beginning with a given letter. Two separate trials were conducted with different letters (L & S), giving a score of number of valid words generated.

Executive Function: The Tower of London task involved participants looking at a pair of pictures of three coloured balls arranged on three pegs, with the balls arranged differently in each picture. The participant was given 20 secs per pair to calculate the minimum number of times the balls in the first picture would be moved in order to make the arrangement of balls identical to the second picture. The task was discontinued if five incorrect answers in a row were given. A score of number of correct responses (out of a total possible 22) was recorded.

The BACS composite score will be calculated as follows. The BACS consists of items across six subtests: Verbal Memory, Digit Sequencing, Token Motor, Verbal Fluency, Symbol Coding, and Tower of London. The so-called subtest scaled score for each subtest will be computed as follows: Let X_jkl_ be the raw score for subject *l* on subtest *j* (1 to 6) at time point *k* (0 = Day -1; 1 = 2 hours post-dose on Day 1; 2 = Day 14). Assume this subject is of sex m (1=male; 2=female) and in age category n (1=20-39; 2=40-49; 3=50-59; 4=60-69; 5=70-79 years). The scaled score for subtest j will be computed as:

C_jkl_ = ( X_jkl_ – M_jmn_ ) / SD_jmn_,

where M_jmn_ and SD_jmn_ are the mean and standard deviation for subtest *j*, respectively, of the index population for sex *m* and age category *n*, the specific values of which can be found in Appendix A. The subtest T-score will then be derived as T_jkl_ = 10 × C_jkl_ + 50.

Finally, the BACS composite score for subject l at time point k is computed as T_kl_ = 10 × AC_kl_ /SD_mn_ + 50, where AC_kl_ is the sum of the 6 scaled subtest scores for subject *l* at time *k* and SD*mn* is the standard deviation of the sum of the 6 scaled subtest scores for sex m and age category *n* (Appendix A).

When up to 2 subtest scores are missing, the sum of the scaled scores will be calculated as (sum of non-missing subtest scaled scores) × (total number of items) / (number of non-missing subtests). If more than 2 subtests in the BACS assessments are missing, the BACS composite score will be set to missing.

## Statistical analysis of numeric endpoints

Each of the primary outcome measures was analysed using a Bayesian normal linear model with effects for sequence, period, treatment, time (as a categorical variable), the treatment-by-time interaction, subject within treatment sequence and baseline (for BACS only). For the BOLD fMRI, the observed value was the response variable in the model. For BACS, the observed and the change from baseline were modelled separately. Missing values were not imputed and were not included in the model under a missing-at-random assumption. A diffuse normal distribution with mean zero and variance 10^6^ was used as a prior for the regression coefficients and a diffuse inverse gamma with shape and scale parameters of 0.01 for the residual variance. The posterior mean, SD and 90% credible interval (highest posterior density interval) were extracted for each treatment and time, along with the posterior mean treatment.

The criteria for a positive result were at least 70% posterior probability of a difference between TAK-041 and placebo of greater than 0.09 in the average of left and right VS activation in the MID fMRI at 3.5 hours post dose on day 1 or at least 70% posterior probability of a difference between TAK-041 and placebo greater than two points in the BACS composite score at day 14. The predefined positivity criterion for the MID task was informed by previously acquired data with the same task at the same imaging centre. The full statistical plan is provided in the Supplementary Information.

A linear mixed-effects model for repeated measures was implemented for each of the exploratory pharmacodynamic measures including PANSS, BNSS and imaging outcomes. For the imaging endpoints, the observed value was the response variable in the model. For the other endpoints, the change from baseline was the response variable, and baseline was used as a covariate in the model.

PK sampling and analysis

Venous blood samples for PK analysis of TAK-041 were collected into chilled blood collection tubes (Vacutainer, BD, Oakville, ON, Canada) containing the anticoagulant ethylenediaminetetraacetic acid (K_2_EDTA). Plasma concentrations of TAK-041 were measured by high-performance liquid chromatography with tandem mass spectrometry over a concentration range of 1–1500 ng/mL. PK parameters were determined from the concentration–time profiles for all evaluable participants and calculated using non-compartmental analysis using Phoenix WinNonlin software, version 8.0 (Certara, Princeton, NJ, USA). Actual sampling times were used in all computations.

Plasma concentration measures (in ng/mL) from the sample taken proximal to the MRI scan on day 1 (5 hours post dose) and day 14 (approximately 312 hours post dose) were included as covariates in relevant imaging analyses, as specified in the Results.

Results

Three participants subjects deviated from the planned timings of the scan during the day 14 session of their TAK-041 treatment period. Two completed the BACS as planned but had to return the following day (day 15) to complete the MRI assessment. One participant was unable to return for the day 14 visit and this was rescheduled to day 25. All other visits were completed as scheduled.

## MID

After imaging and task quality control checks, one participant exhibited excessive head movement on both day 1 scans and was excluded. Two participants did not complete the task to a sufficient standard to allow satisfactory modelling of the fMRI data and were also excluded: one responded to less than 50% of active trials on the MID on both their day 14 scans, and one did not respond to any neutral trials during their placebo day 14 scan, meaning that their task data could not be accurately modelled. One participant was withdrawn from the study after their placebo session and did not complete period 2; one participant withdrew from the study after their initial day 1 visit; and one was unable to complete their initial day 1 scan.

### Behavioural

All participants in the final analysis performed the task to a high standard, responding to at least 88% of trials that required a response, and there was no significant difference between attention to the task between treatment sessions, time or dose (Supplementary tables 3 and 4).

Although participants in the placebo sessions on average won slightly more on the task, a repeated- measures ANOVA with dose level as a between-subject level factor revealed there was no significant main effect of drug (*F*(1,15) = 2.257, *p* = 0.154), time (*F*(1,15) = 0.245, *p* = 0.628) or drug*time interaction (*F*(1,15) = 0.832, *p* = 0.376) in the total amount won between the groups (Supplementary Table 4).

Hit-rate analysis (the percentage of monetised trials within each reward level that the participant successfully responds to within the RT window) allows examination of performance across reward levels. Placebo sessions had a higher hit rate over both time points: a repeated-measures ANOVA with drug (TAK-041 or placebo) and reward (low or high) as within-subject factors and dose level (40 mg or 160 mg) as a between-subject level factor revealed a significant main effect of reward (*p*= 0.036), but no significant effect of drug (*p* = 0.150), or drug*reward-level*dose interaction for day 1. Day 14 analysis revealed no significant effect of drug, reward level or interaction.

To allow a sufficient number of win and lose trials, the paradigm alters the RT window for each trial depending on previous task performance to move each participant towards a 66% win rate and, therefore, absorbs performance differences to an extent. Examining RT allows analysis of overall performance without the effect of the moving RT window. Repeated-measures ANOVAs with drug (TAK-041 or placebo) and reward (neutral, low or high) as within-subject factors and dose level (40 mg or 160 mg) as a between-subject level factor revealed a significant main effect of reward at day 1 (*p* < 0.001) and day 14 (*p* < 0.001), but no effect of drug at either time point.

### Whole brain analysis

To explore the effect of time, the delta images were analysed in factorial model in SPM, with day 1/day 14 as a within-subjects factor and dose as a between-subjects factor, which revealed no main effect of time or dose, or interaction. Owing to the small number of participants of the 40 mg group, days 1 and 14 delta images for each participant were also entered into a paired *t*-test with TAK-041 blood plasma concentration (ng/mL) as a covariate, but did not reveal any significant results either at whole-brain or SVC level.

To examine any relationship between BOLD response and clinical or behavioural assessments, exploratory correlations were conducted between extracted beta scores with the PANSS negative subscales scores and BNSS domains, and with the composite score on the BACS. No significant correlations were found after correction for multiple comparisons. However, it is of note that prior to correction day 14, reward-anticipation beta values were found to be negatively correlated with the day 14 avolition subscale on the BNSS.

Delta images of the reward feedback contrast did not reveal any effect of drug at Day 1 or Day 14 on feedback processing, either at whole brain or at SVC within the ventral striatum ROI.

### ASL

Complete two-run ASL acquisitions were not completed for three participants during Day-14 scans due to the scanning session overrunning, extensive head movement producing unresolvable image artifact and coregistration failure during preprocessing on one or more of the runs.

A one-sample *t*-test of day 1 placebo versus TAK-041 delta images revealed widespread and unidirectional grey matter reductions in CBF after a single dose of TAK-041 at day 1, comprising of two large clusters with peaks around the left middle temporal gyrus/lateral occipital cortex and left insula, and right parahippocampal gyrus and hippocampus. Within the most prominent cluster (k = 9516, p < 0.001 FWE) localised to the left temporal lobe, there were peaks centred around the temporooccipital and anterior parts of the left middle temporal gyrus ([-60 −62 3] mm, t = 6.74; [-64, -7, -9] mm, t = 6.10) and left insula cortex ([-41, -7, -18], t = 5.97). The other large cluster (k = 2228, p < 0.001 FWE) was centred around the right parahippocampal gyrus and hippocampus (peaks at [24,2,-42] mm, t = 6.19; [36, 9, -36] mm, t = 5.23; [26, -6, -27], t = 4.95), with a third smaller cluster (k = 1417, p = 0.002 FWE) localised to the right superior and middle frontal gyrus (peaks at [6, 13, 69] mm, t = 4.52; [39, 4, 63] mm, t = 4.48; [19, -6, 54], t = 4.43.

The same analysis of the day 14 scans failed to reveal any significant changes in CBF due to TAK-041.

**Table S1:** Clinical scales

|  | PANSS | Negative Subscale Score | | Positive Subscale Score | | BNSS | Total score | |
| --- | --- | --- | --- | --- | --- | --- | --- | --- |
| Visit | N | Mean | (SD) | Mean | (SD) | N | Mean | (SD) |
| Placebo |  |  |  |  |  |  |  |  |
| Day -1 | 21 | 19.86 | 3.65 | 14.71 | 4.83 | 20.00 | 26.65 | 8.57 |
| Day 14 | 21 | 18.95 | 3.37 | 14.81 | 4.98 | 20.00 | 24.55 | 8.93 |
| TAK-041 40mg |  |  |  |  |  |  |  |  |
| Day -1 | 7 | 21.57 | 3.69 | 15.14 | 5.05 | 6.00 | 24.17 | 8.57 |
| Day 14 | 6 | 20.50 | 4.51 | 14.50 | 4.72 | 6.00 | 24.17 | 13.29 |
| TAK-041 160mg |  |  |  |  |  |  |  |  |
| Day -1 | 15 | 19.53 | 2.92 | 15.07 | 4.65 | 14.00 | 24.29 | 7.66 |
| Day 14 | 14 | 18.71 | 2.73 | 14.36 | 4.57 | 14.00 | 25.71 | 9.11 |
| TAK-041 Total |  |  |  |  |  |  |  |  |
| Day -1 | 22 | 20.18 | 3.25 | 15.09 | 4.66 | 20.00 | 24.25 | 7.71 |
| Day 14 | 20 | 19.25 | 3.34 | 14.40 | 4.49 | 20.00 | 25.25 | 10.19 |

**Table S2**: BACS composite score at each testing session.

|  | Visit | N | Observed value at visit | Change from baseline |
| --- | --- | --- | --- | --- |
| **Placebo** | Baseline | 21 | 29.72 (12.87) | n/a |
|  | Day 1 | 21 | 31.08 (10.42) | 1.36 (5.831) |
|  | Day 14 | 21 | 32 (11.09) | 2.28 (6.963) |
| **TAK-041 40mg** | Baseline | 7 | 27.35 (12.75) | n/a |
|  | Day 1 | 7 | 28.71 (11.68) | 1.35 (6.56) |
|  | Day 14 | 6 | 34.34 (13.34) | 5.35 (6.94) |
| **TAK-041 160mg** | Baseline | 15 | 27.89 (8.21) | n/a |
|  | Day 1 | 15 | 30.28 (9.232) | 2.39 (6.42) |
|  | Day 14 | 14 | 29.76 (5.934) | 1.31 (5.62) |
| **TAK-041 overall** | Baseline | 22 | 27.72 (9.57) | n/a |
|  | Day 1 | 22 | 29.78 (9.82) | 2.06 (6.33) |
|  | Day 14 | 20 | 31.14 (8.7) | 2.52 (6.16) |

*Baseline is defined as the last observation prior to the dose of study drug in the corresponding period. BACS, Brief Assessment of Cognition in Schizophrenia; SD, standard deviation.*

**Table S3:** Percentage response rate across sessions on MID task

| Day 1 Response Rate Placebo (n=20) | Day 1 Response Rate TAK041 (n=19) | Day 14 Response Rate Placebo (n=19) | Day 14 Response Rate TAK041 (n=19) |
| --- | --- | --- | --- |
| 88.68 (15.47) | 91.6 (11.91) | 93.41 (6.82) | 95.32 (5.61) |

**Table S4:** MID task performance (SD) for hit rate, reaction time and amount won, collasped across dose level

|  |  | Hit Rate (%) | | | Reaction Time (sec) | | | |  |
| --- | --- | --- | --- | --- | --- | --- | --- | --- | --- |
|  |  | Low Reward | High Reward | Overall | Neutral Reward | Low Reward | High Reward | Overall | Total Won (£) |
| Day 1 | Placebo (n=20) | 53.45 (21.26) | 58.91 (25.21) | 56.18 (21) | .342 (.05) | .326 (.039) | .314 (.044) | .328 (.043) | 30 (11.98) |
|  | TAK041 (n=19) | 43.84 (18.72) | 53.53 (26.38) | 48.68 (20.92) | .337 (.045) | .336 (.050) | .324 (.049 | .332 (.046) | 28.94 (11.50) |
| Day 14 | Placebo (n=19) | 52.94 (16.61) | 62.8 (20.73) | 57.87 (16.56) | .33 (.037) | .324 (.032) | .313 (.038) | .324 (.034) | 32.93 (9.72) |
|  | TAK041 (n=19) | 50.42 (19.06) | 53.55 (22.04) | 51.99 (17.87) | .330 (.029) | .319 (.034) | .311 (.030) | .320 (.029) | 30.21 (9.48) |


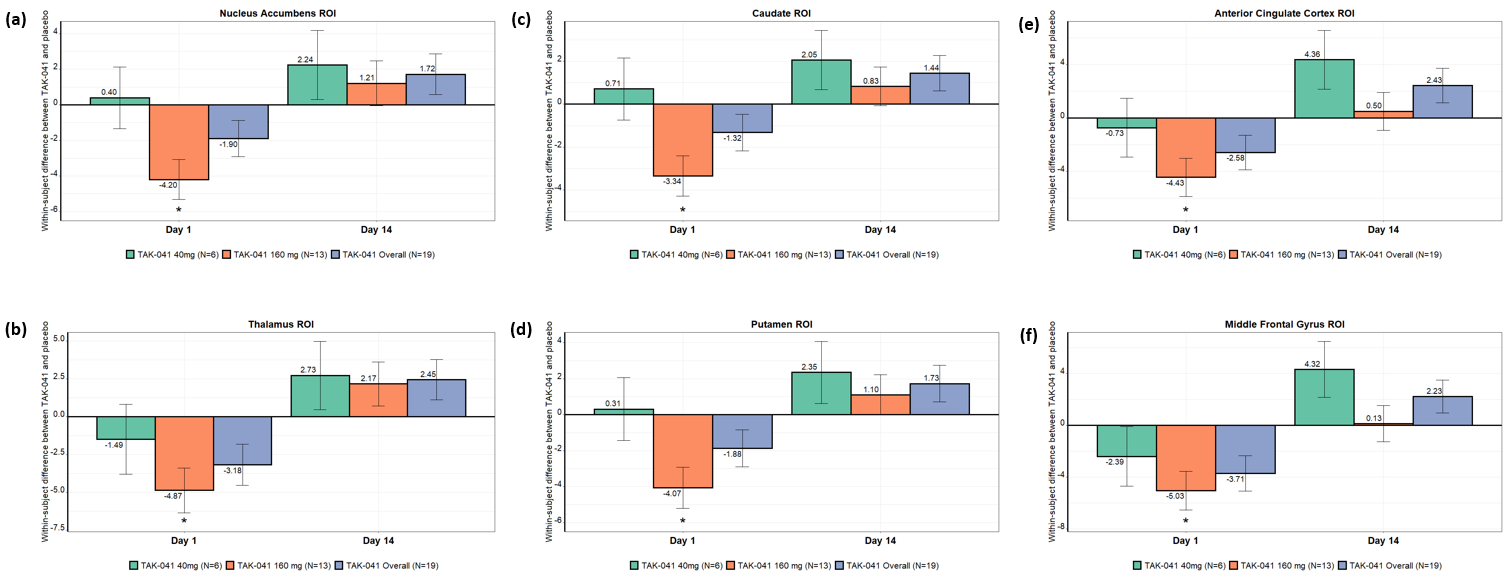


**Figure S1:** Modelled within-subject differences (mean±SE) in CBF between TAK-041 and placebo for predefined regions of interest. *p<0.05


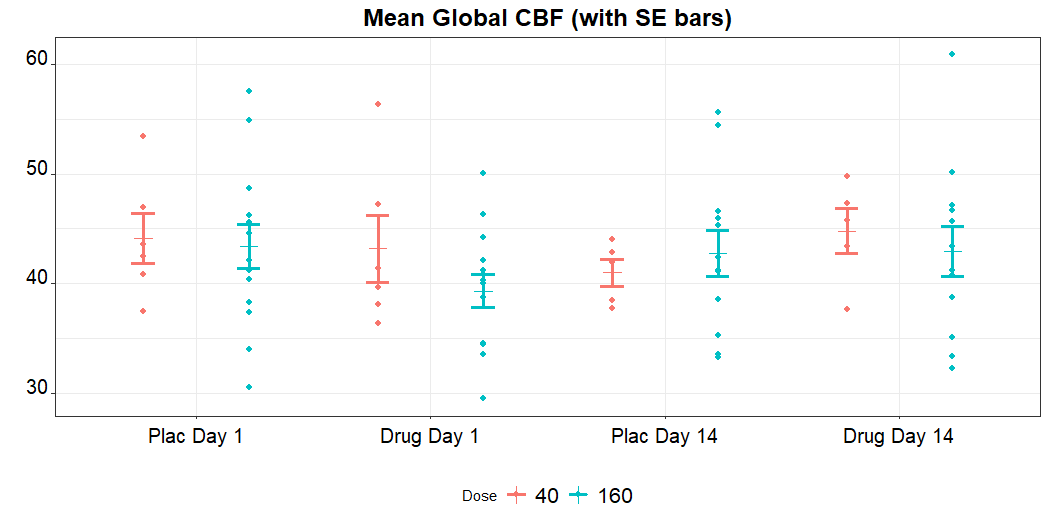


**Figure S2:** Mean global CBF values by condition and measurement day. Dots represent individual subjects, lines represent mean ±SE.


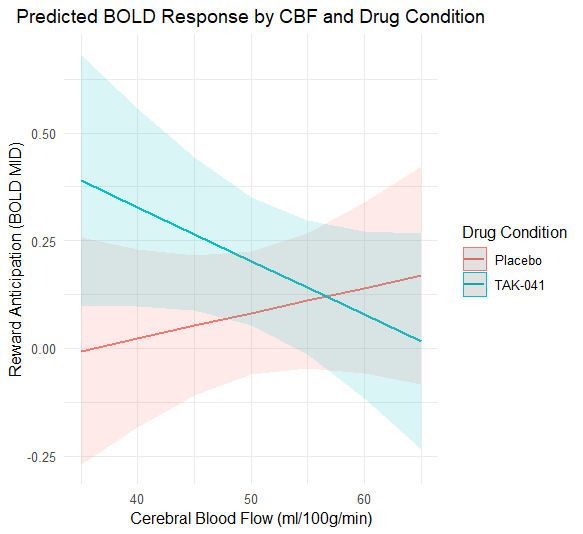


**Figure S3**: Interaction plot illustrating predicted BOLD response by CBF and Drug condition (treatment*CBF interaction (F(1,46) = 5.84, p = 0.020).

# Adverse events

**Table S5:** Most Frequent TEAEs (≥2% of Subjects in Either TAK-041 Treatment Arm)


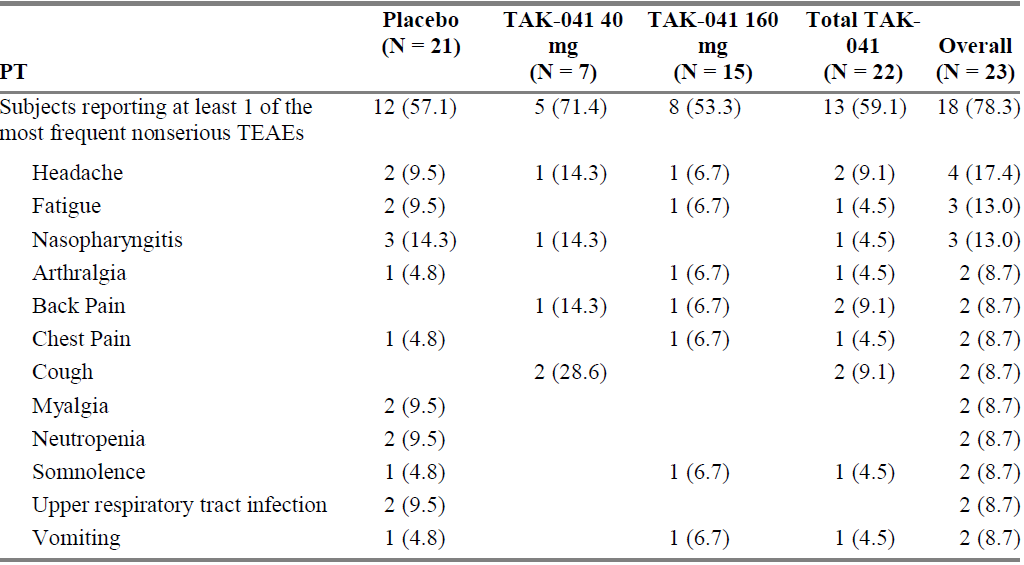


Overall, the most frequent TEAEs (experienced by at least 2 subjects per PT) were reported in 12 of 21 placebo-treated subjects (57.1%) and 13 of 22 TAK-041-treated subjects (59.1%) during the study (Table s5). The trend of AEs was similar in placebo and TAK-041 subjects. No dose dependency of TAK-041 was evident in this small study. Three placebo-treated subjects (14.3%) and 3 TAK-041–treated subjects (13.6%) experienced TEAEs considered related to study drug by the investigator. The majority (56.5%) of the 18 TEAEs reported were mild, with 5 (21.7%) moderate and 0% severe reported. Headache was the most commonly reported TEAE overall (17.4%), followed by fatigue and nasopharyngitis, both at 13.0%.

One subject in Sequence 3 experienced an AE of syncope with moderate intensity after administration of TAK-041-160 mg in Period 1, which was considered vasovagal syncope. The event was short-lived and resolved without treatment.

Appendix A Population Norms of the Brief Assessment of Cognition in Schizophrenia (BACS)

| **Test/Sex/Age Group** |  | **20-39** | **40-49** | **50-59** | **60-69** | **70-79** |
| --- | --- | --- | --- | --- | --- | --- |
| **Male** |  |  |  |  |  |  |
| **Verbal Memory** | Mean | 47.40 | 45.27 | 40.71 | 41.96 | 35.43 |
|  | SD | 8.94 | 9.20 | 8.76 | 10.28 | 8.15 |
| **Digit Sequencing** | Mean | 22.46 | 22.21 | 21.69 | 21.61 | 19.22 |
|  | SD | 3.36 | 3.99 | 3.86 | 4.41 | 3.77 |
| **Token Motor Task** | Mean | 75.36 | 65.87 | 60.78 | 60.78 | 52.10 |
|  | SD | 10.96 | 14.38 | 14.59 | 13.88 | 15.17 |
| **Verbal Fluency** | Mean | 53.60 | 53.09 | 50.04 | 51.91 | 42.09 |
|  | SD | 10.87 | 11.65 | 12.15 | 14.63 | 12.83 |
| **Symbol Coding** | Mean | 64.38 | 54.10 | 50.32 | 51.30 | 42.52 |
|  | SD | 11.37 | 11.30 | 11.28 | 8.69 | 11.09 |
| **Tower of London** | Mean | 17.79 | 17.33 | 16.92 | 15.82 | 15.14 |
|  | SD | 2.72 | 3.52 | 3.17 | 4.01 | 5.07 |
| **Composite Score** | SD | 3.45 | 4.14 | 3.81 | 3.94 | 4.14 |
| **Female** |  |  |  |  |  |  |
| **Verbal Memory** | Mean | 51.96 | 50.71 | 46.70 | 45.61 | 41.11 |
|  | SD | 7.05 | 8.27 | 8.16 | 10.79 | 7.23 |
| **Digit Sequencing** | Mean | 21.41 | 21.92 | 20.91 | 18.29 | 19.33 |
|  | SD | 4.12 | 3.57 | 3.82 | 3.56 | 3.50 |
| **Token Motor Task** | Mean | 76.67 | 75.98 | 68.29 | 67.79 | 57.04 |
|  | SD | 11.03 | 13.99 | 14.07 | 11.76 | 12.81 |
| **Verbal Fluency** | Mean | 52.76 | 55.13 | 51.83 | 47.29 | 46.56 |
|  | SD | 10.56 | 13.14 | 11.05 | 12.78 | 9.55 |
| **Symbol Coding** | Mean | 66.61 | 58.62 | 57.00 | 49.04 | 45.08 |
|  | SD | 10.40 | 11.31 | 9.69 | 9.69 | 6.44 |
| **Tower of London** | Mean | 17.78 | 16.38 | 16.40 | 15.68 | 16.00 |
|  | SD | 2.36 | 4.81 | 3.47 | 4.32 | 2.17 |
| **Composite Score** | SD | 3.88 | 3.80 | 3.57 | 3.66 | 3.51 |

# Appendix B: TAKEDA PHARMACEUTICALS PROTOCOL

**A Randomized, Double-Blind, Placebo Controlled, Two-Period Cross-Over, Proof of Activity Study to Evaluate the Effects of TAK-041 on Motivational Anhedonia as**

**Add-On to Antipsychotics in Subjects With Stable Schizophrenia**

| **Sponsor:** Takeda Development Centre Europe Ltd.  61 Aldwych |  |  |
| --- | --- | --- |
| London, WC2B 4AE  United Kingdom |  |  |
| **Study Number:** TAK-041-2001 |  |  |
| **Compound:** TAK-041 |  |  |
| **Date:** 10 August 2018 **Version/Amendment Number:** | 04 |  |
| **Amendment History:** |  |  |
| **Amendment Type (for regional**  **Date Amendment Number European purposes only)** |  | **Region** |
| 25 April 2017 Initial version Not applicable |  | Global |
| 24 July 2017 01 Non-Substantial |  | Global |
| 07 September 2017 02 Substantial |  | Global |
| 02 April 2018 03 Substantial |  | Global |
| 10 August 2018 04 Substantial |  | Global |

**CONFIDENTIAL PROPERTY OF TAKEDA**

This document is a confidential communication of Takeda. Acceptance of this document constitutes the agreement by the recipient that no information contained herein will be published or disclosed without written authorization from Takeda except to the extent necessary to obtain informed consent from those persons to whom the drug may be administered. Furthermore, the information is only meant for review and compliance by the recipient, his or her staff, and applicable institutional review committee and regulatory agencies to enable conduct of the study.

## TABLE OF CONTENTS

- 1. [STUDY SUMMARY 7](#_bookmark0)
  2. [Protocol Amendment 04 13](#_bookmark1)

[2.0 STUDY SCHEMATIC 14](#_bookmark13)

[3.0 SCHEDULE OF STUDY PROCEDURES 15](#_bookmark15)

- 1. [INTRODUCTION 24](#_bookmark17)
  2. [Background 24](#_bookmark18)
  3. [Rationale for the Proposed Study 24](#_bookmark19)
  4. [Benefit/Risk Profile 25](#_bookmark20)
  5. [STUDY OBJECTIVES AND ENDPOINTS 26](#_bookmark21)
  6. [Objectives 26](#_bookmark22)
     1. [Primary Objective 26](#_bookmark23)
     2. [Secondary Objectives 26](#_bookmark24)
     3. [Exploratory Objectives 26](#_bookmark25)
  7. [Endpoints 26](#_bookmark26)
     1. [Primary Endpoints 26](#_bookmark27)
     2. [Secondary Endpoints 27](#_bookmark28)
  8. [Exploratory Endpoints 27](#_bookmark29)
  9. [STUDY DESIGN AND DESCRIPTION 29](#_bookmark30)
  10. [Study Design 29](#_bookmark31)
  11. [Rationale for Study Design, Dose, and Endpoints 31](#_bookmark33)
      1. [Rationale of Study Design and Regimen 31](#_bookmark34)
      2. [Rationale for Dose 31](#_bookmark35)
      3. [Rationale for Endpoints 32](#_bookmark36)
      4. [Critical Procedures Based on Study Objectives: Timing of Procedures 35](#_bookmark37)
  12. [Study Design/Dosing/Procedures Modifications Permitted Within Protocol Parameters 36](#_bookmark38)
  13. [Study Beginning and End/Completion 37](#_bookmark39)
      1. [Definition of Beginning of the Study 37](#_bookmark40)
      2. [Definition of End of the Study 37](#_bookmark41)
      3. [Definition of Study Completion 37](#_bookmark42)
      4. [Definition of Study Discontinuation 37](#_bookmark43)
      5. [Criteria for Premature Termination or Suspension of the Study 37](#_bookmark44)
  14. [SELECTION AND DISCONTINUATION/WITHDRAWAL OF SUBJECTS 39](#_bookmark45)
  15. [Inclusion Criteria 39](#_bookmark46)
  16. [Exclusion Criteria 39](#_bookmark47)
  17. [Excluded Medications, Supplements, and Dietary Products 41](#_bookmark48)
  18. [Diet, Fluid, Activity 44](#_bookmark50)
      1. [Diet and Fluid 44](#_bookmark51)
      2. [Activity 44](#_bookmark52)
  19. [Criteria for Discontinuation or Withdrawal of a Subject 44](#_bookmark53)
  20. [Procedures for Discontinuation or Withdrawal of a Subject 46](#_bookmark54)
  21. [Subject Replacement 46](#_bookmark55)
  22. [CLINICAL STUDY MATERIAL MANAGEMENT 47](#_bookmark56)
  23. [Clinical Study Drug 47](#_bookmark57)
      1. [Investigational Drug 47](#_bookmark58)
      2. [Storage 47](#_bookmark61)
      3. [Dose and Regimen 48](#_bookmark62)
      4. [Overdose 48](#_bookmark63)
      5. [Clinical Study Drug Labeling 48](#_bookmark64)
      6. [Clinical Study Drug Inventory 48](#_bookmark65)
      7. [Clinical Study Drug Blinding 48](#_bookmark66)
      8. [Randomization Code Creation and Storage 48](#_bookmark67)
      9. [Clinical Study Blind Maintenance/Unblinding Procedure 49](#_bookmark68)
      10. [Accountability and Destruction of Sponsor-Supplied Drugs 49](#_bookmark69)
  24. [STUDY PROCEDURES 50](#_bookmark70)
  25. [Administrative Procedures 50](#_bookmark71)
      1. [Informed Consent Procedure 50](#_bookmark72)
      2. [Inclusion and Exclusion Criteria 50](#_bookmark73)
      3. [Medical History/Demographics 51](#_bookmark74)
      4. [Prior and Concomitant Medication Review 51](#_bookmark75)
  26. [Clinical Procedures and Assessments 51](#_bookmark76)
      1. [Full Physical Exam 51](#_bookmark77)
      2. [Height and Weight 51](#_bookmark78)
      3. [Body Mass Index 51](#_bookmark79)
      4. [Vitals (Body Temperature, Heart Rate, Blood Pressure) 51](#_bookmark80)
      5. [12-Lead ECG 51](#_bookmark81)
      6. [Study Drug Administration 52](#_bookmark82)
      7. [Cognitive Assessments 52](#_bookmark83)
      8. [AE Monitoring 53](#_bookmark84)
      9. [Actigraphy Monitoring 53](#_bookmark85)
      10. [Laboratory Procedures and Assessments 53](#_bookmark86)
      11. [C-SSRS 55](#_bookmark88)
  27. [Biomarker, PK, PD, and PGx Samples 55](#_bookmark89)
      1. [PK Measurements 55](#_bookmark91)
      2. [Biomarker Measurements 56](#_bookmark92)
      3. [PGx Measurements 56](#_bookmark93)
      4. [Confinement 57](#_bookmark94)
  28. [ADVERSE EVENTS 58](#_bookmark95)
  29. [Definitions and Elements of AEs 58](#_bookmark96)
      1. [SAEs 60](#_bookmark97)
  30. [AE Procedures 61](#_bookmark99)
      1. [Assigning Severity/Intensity of AEs 61](#_bookmark100)
      2. [Assigning Causality of AEs 61](#_bookmark101)
      3. [Start Date 61](#_bookmark102)
      4. [Stop Date 61](#_bookmark103)
      5. [Frequency 61](#_bookmark104)
      6. [Action Concerning Study Drug 61](#_bookmark105)
      7. [Outcome 62](#_bookmark106)
      8. [Collection and Reporting of AEs, SAEs, Special Interest AEs, and Abnormal LFTs 62](#_bookmark107)
      9. [Safety Reporting to Investigators, IRBs or IECs, and Regulatory Authorities 64](#_bookmark110)
  31. [STATISTICAL METHODS 65](#_bookmark111)
  32. [Statistical and Analytical Plans 65](#_bookmark112)
      1. [Analysis Sets 65](#_bookmark113)
      2. [Analysis of Demographics and Other Baseline Characteristics 65](#_bookmark114)
      3. [PK Analysis 65](#_bookmark115)
      4. [PD Analysis 66](#_bookmark116)
      5. [Safety Analysis 66](#_bookmark117)
  33. [Interim Analysis and Criteria for Early Termination 67](#_bookmark118)
  34. [Determination of Sample Size 67](#_bookmark119)
  35. [QUALITY CONTROL AND QUALITY ASSURANCE 68](#_bookmark120)
  36. [Study-Site Monitoring Visits 68](#_bookmark121)
  37. [Protocol Deviations 68](#_bookmark122)
  38. [Quality Assurance Audits and Regulatory Agency Inspections 68](#_bookmark123)
  39. [ETHICAL ASPECTS OF THE STUDY 69](#_bookmark124)
  40. [IRB and/or IEC Approval 69](#_bookmark125)
  41. [Subject Information, Informed Consent, and Subject Authorization 70](#_bookmark126)
  42. [Subject Confidentiality 71](#_bookmark127)
  43. [Publication, Disclosure, and Clinical Trial Registration Policy 71](#_bookmark128)
      1. [Publication and Disclosure 71](#_bookmark129)
      2. [Clinical Trial Registration 72](#_bookmark130)
      3. [Clinical Trial Results Disclosure 72](#_bookmark131)
  44. [Insurance and Compensation for Injury 72](#_bookmark132)
  45. [ADMINISTRATIVE AND REFERENCE INFORMATION 73](#_bookmark133)
  46. [Administrative Information 73](#_bookmark134)
      1. [Study Contact Information 73](#_bookmark135)
      2. [Investigator Agreement 74](#_bookmark136)
      3. [Study-Related Responsibilities 75](#_bookmark137)
      4. [List of Abbreviations 76](#_bookmark138)
  47. [DATA HANDLING AND RECORDKEEPING 78](#_bookmark139)
  48. [CRFs (Electronic and Paper) 78](#_bookmark140)
  49. [Record Retention 78](#_bookmark141)

[16.0 REFERENCES 80](#_bookmark142)

[17.0 APPENDICES 82](#_bookmark160)

**LIST OF IN-TEXT TABLES**

[Table 3.a Schedule of Study Procedures 15](#_bookmark16)

[Table 6.a Sequence Groups 30](#_bookmark32)

[Table 7.a Excluded Medications, Supplements, and Dietary Products 41](#_bookmark49)

[Table 8.a Composition of Tween/MC Vehicle 47](#_bookmark59)

[Table 8.b Composition of Crystalline TAK-041 Oral Suspension 47](#_bookmark60)

[Table 9.a Primary Specimen Collections 55](#_bookmark90)

[Table 10.a Takeda Medically Significant AE List 60](#_bookmark98)

## LIST OF IN-TEXT FIGURES

[Figure 2.a TAK-041-2001 Study Schematic 14](#_bookmark14)

## LIST OF APPENDICES

[Appendix A Responsibilities of the Investigator 82](#_bookmark161)

[Appendix B Elements of the Subject Informed Consent 84](#_bookmark162)

[Appendix C Investigator Consent to Use of Personal Information 87](#_bookmark163)

[Appendix D Pregnancy and Contraception 88](#_bookmark164)

[Appendix E Detailed Description of Amendments to Text 90](#_bookmark165)

## STUDY SUMMARY

| **Name of Sponsor:**  Takeda Development Centre Europe Ltd. 61 Aldwych  London, WC2B 4AE United Kingdom | **Compound:**  TAK-041 |
| --- | --- |
| **Study Number:**  TAK-041-2001 | **Phase:**1 |
| **Protocol Title:** A Randomized, Double-Blind, Placebo Controlled, Two-Period Cross-Over, Proof of Activity Study to Evaluate the Effects of TAK-041 on Motivational Anhedonia as Add-On to Antipsychotics in Subjects With Stable Schizophrenia | |
| **Study Design:**  This study is a randomized, double-blind, placebo-controlled, 2-period, crossover phase 2 study to evaluate the pharmacodynamic (PD) effects, safety, tolerability, and pharmacokinetics (PK) of single doses of oral TAK-041 in adult subjects with schizophrenia, particularly negative symptoms characterized by reduced motivation. The study is designed to evaluate the effect of TAK-041, a G protein-coupled receptor 139 orphan receptor agonist, as an add-on to antipsychotics in attenuating the impairment in motivational anhedonia as well as cognitive function using motivation/reward battery tests as well as brain imaging (ie, task-induced functional Magnetic Resonance Imaging [fMRI] Blood-Oxygen-Level-Dependent [BOLD] and arterial spin labeling [ASL]) in subjects with stable schizophrenia. Subjects should be stable on their existing medicine (ie, second generation antipsychotics) for at least 2 months prior to screening. A planned unblinded interim analysis (IA) for efficacy may be conducted when approximately12 subjects have completed the Day 14 procedures in both periods at sponsors discretion. The IA objective is to determine whether to continue the TAK-041 dose after the IA, or revise to a different dose, based on the results of the IA. Any dose change to explore dose/exposure relationships on the PD endpoints at other dose levels will be supported by emerging safety, tolerability and PK data.  The study will consist of 2 treatment periods, with a single dose of study drug administered in each period. There will be a 35-day (+7 days) washout interval between the 2 doses to reduce the potential for residual TAK-041 to impact the PD endpoints. Treatment Period 2 begins at the end of the Treatment Period 1 washout. Because subjects are not confined in this study, they will come to the clinic for the following visits:   - Screening Visit (between Days -35 to -2) covering full medical and psychiatric examinations. - Baseline Assessments Visit (Day -1 in each Treatment Period), covering motivation/reward and cognitive testing baseline, except fMRI. - Dosing and First Testing Visit (Day 1 in each Treatment Period). - Second Testing Visit (Day 14 in each Treatment Period). - Follow-up Visit (Day 49 [ ±4 days] for Treatment Period 2 only). - End of Period 1 Visit (Day 35 [+7 days] postdose in Period 1). This visit will serve to conduct end of Period 1 procedures and the Day -1 Baseline assessments for Period 2. - Study Exit occurs at the Final Visit (77 ±7 days postdose in Period 2).   The trial population will include subjects with stable schizophrenia aged 18 to 60 years, inclusive, considered eligible on the basis of the trial inclusion and exclusion criteria. The trial will enroll up to 32 subjects dosed to ensure  24 subjects complete.  On Day 1 of Period 1, eligible subjects will be randomized in a ratio of 1:1 to 1 of the 2 treatment sequences (shown below) and will receive each trial treatment according to the randomized sequence group. The initial dose of TAK-041 will be 40 mg. Higher doses will be considered depending on emerging safety, tolerability and PK data. After the IA, the dose level may also be changed based on the IA results. The decision criteria will be predefined in the SAP before unblinding. | |

| **Sequence** | **Period 1** | **Period 2** |
| --- | --- | --- |
| 1 | TAK-041 + antipsychotic | Placebo + antipsychotic |
| 2 | Placebo + antipsychotic | TAK-041 + antipsychotic |
| During the Screening Visit (Days -35 to -2), subjects meeting Diagnostic and Statistical Manual of Mental Disorders fifth edition (DSM-5) criteria for schizophrenia and on stable antipsychotic therapy for at least 2 months will complete medical and psychiatric examinations, electrocardiogram (ECG), and laboratory safety tests. At the Baseline Assessments Visit (Day -1), subjects will complete practice sessions of the motivation/reward and cognitive battery in order to minimize potential practice effects at subsequent treatment visits, will be familiarized with the magnetic resonance imaging (MRI) environment, and further will be tested for baseline assessments (except fMRI). Practice sessions will be conducted in Period 1 only. Subjects meeting all inclusion criteria and no exclusion criteria will be administered study medication or placebo in the clinic based on the randomization schedule on Day 1 of Treatment Periods 1 and 2.  On Day 1 of each Treatment Period, following study drug administration, subjects will take PD tests for a total of approximately 4 hours and blood samples will be collected at various times after dosing to assess the PK of TAK-041. After the last PK collection at 6 hours postdose, subjects will leave the clinic. On Day 14 of each Treatment Period, subjects will take the second set of PD tests along with a single time point PK collection. Samples for PK will also be collected on Day 49 and Final Visit of the second Treatment Period. There will be a washout period of 35 days (+ 7 days) between doses in each Treatment Period to reduce the potential for residual TAK-041 to impact the PD endpoints.  PD testing will be performed in 3 blocks, separated by two breaks. The first block will comprise the Grip Effort Task, the Progressive Ratio Test, and the Brief Assessment of Cognition in Schizophrenia (BACS). After a break, subjects will take the second block that comprises a battery of imaging tests; namely, brain perfusion using noncontrast ASL MRI and fMRI scans which monitor changes in BOLD signal. The fMRI will be run in resting state without a task (rs-fMRI) and with 2 tasks (t-fMRI) using the Monetary Incentive Delay (MID) Reward Task and a Set-Shifting Task. After a break, subjects will take the third block which comprises the Effort Expenditure for Rewards Task (EEfRT) and the Empathic Accuracy Task. Subjects will also receive the Positive and Negative Syndrome Scale (PANSS), the Brief Negative Syndrome Scale (BNSS), and the Clinical Global Impression (CGI) Scales (CGI-Severity and  CGI-Improvement) as a part of the PD assessment. Subject’s activity and sleep rhythms will also be captured with wearable digital devices after TAK-041 or placebo administration. Note that the EEfRT and Empathic Accuracy tasks, as well as the wearable devices, are designated as optional assessments due to the need to demonstrate the operational feasibility of conducting these tasks and devices at the research sites. These tasks will only be included if the feasibility and validity of data generated by the tasks are confirmed. If included in the study procedures, these tasks will be conducted only in participants randomized to study treatment after the feasibility of the task (or tasks) and devices have been demonstrated.  Blood will be drawn for exploratory PK analysis on Day 1 of each Treatment Period at the following times relative to the TAK-041 or placebo dose: 0 (within 30 min prior to TAK-041/placebo dosing), 1, 2, 5, and 6 hours postdose. In addition, single time point PK will be collected in both Periods at the Second Testing Visit (Day 14), end of period 1 washout visit (Day 35 + 7 days), Follow-up Visit (Day 49 ±4 days for Period 2), Final Visit (Day 77 ±7 days), and at early termination if it occurs before Day 49 (for Period 2).  Pharmacogenomics assessments will be performed from the blood samples collected on Day 1 (prior to TAK-041 or placebo administration) for DNA in Period 1 only, and in both Treatment Periods for RNA on Day 1 (prior to  TAK-041 or placebo administration) and on Day 14.  Typical treatment, testing, and washout periods are illustrated below. Testing day scheduled times are estimates of time in minutes required to complete the procedures. | | |


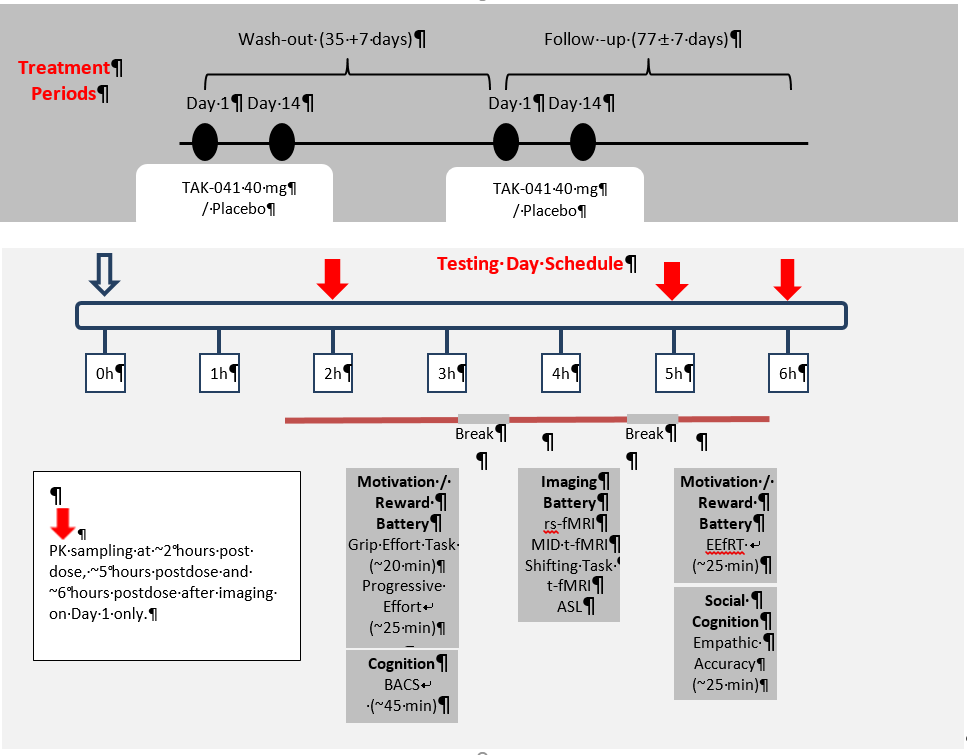


**Primary Objectives:**

- To determine whether motivation/reward deficits observed in schizophrenia are attenuated by add-on TAK-041 administration to antipsychotics in subjects with stable schizophrenia.
- To determine whether cognitive impairment associated with schizophrenia is improved by add-on TAK-041 administration to antipsychotics in subjects with stable schizophrenia.

**Primary Hypotheses:**

- TAK-041 demonstrates greater increase compared with placebo in BOLD signal in the average of left and right ventral striatum (VS) activation in the MID Reward Task (high posterior probability of [a difference between TAK-041 and placebo >0.09] is expected).
- TAK-041 demonstrates greater increase than placebo in the BACS composite score (high posterior probability of [a difference between TAK-041 and placebo > 2.0] is expected).

**Secondary Objectives:**

To determine safety and tolerability of TAK-041 as add-on therapy to antipsychotics in subjects with stable schizophrenia.

| **Primary Objectives:**   - To determine whether motivation/reward deficits observed in schizophrenia are attenuated by add-on TAK-041 administration to antipsychotics in subjects with stable schizophrenia. - To determine whether cognitive impairment associated with schizophrenia is improved by add-on TAK-041 administration to antipsychotics in subjects with stable schizophrenia.   **Primary Hypotheses:**   - TAK-041 demonstrates greater increase compared with placebo in BOLD signal in the average of left and right ventral striatum (VS) activation in the MID Reward Task (high posterior probability of [a difference between TAK-041 and placebo >0.09] is expected). - TAK-041 demonstrates greater increase than placebo in the BACS composite score (high posterior probability of [a difference between TAK-041 and placebo >2.0] is expected).   **Secondary Objectives:**   - To determine safety and tolerability of TAK-041 as add-on therapy to antipsychotics in subjects with stable schizophrenia. | |
| --- | --- |
| **Subject Population:** Male and female subjects with schizophrenia who are on a stable dose of an antipsychotic for at least 2 months prior to screening. | |
| **Number of Subjects:**  Up to 32 subjects enrolled to ensure 24 subjects complete postdose PD assessments on Day 1 in both Treatment Periods | **Number of Sites:**  Approximately 4 sites |
| **Dose Levels:**  TAK-041)/matching placebo: initially 40 mg followed by a potentially higher dose if supported by emerging safety, tolerability and PK data | **Route of Administration:**  Oral |
| **Duration of Treatment:**  Single oral dose on Day 1 of each Treatment Period | **Period of Evaluation:**  126 to 154 days |
| **Main Criteria for Inclusion:**  In order to be eligible for participation, subjects must:   1. Be on a stable dose of an antipsychotic for at least 2 months as documented by medical history and assessed by site staff (other than those on the excluded medication list). 2. Meet schizophrenia criteria as defined by the DSM-5 by the Mini International Neuropsychiatric Interview. 3. Have PANSS total score ≤90 and PANSS negative symptom factor score (NSFS; Sum of PANSS N1, N2, N3, N4, N6, G7, and G16) ≥15 at screening and baseline (Day -1). 4. Have stable screening and baseline PANSS and NSFS total scores (<20% change). 5. Have had a structural brain MRI within the preceding year or during screening indicating no concerning structural brain abnormalities or other abnormalities that would interfere with interpretation of functional brain imaging results. | |
| **Main Criteria for Exclusion:**  The subject must be excluded from participating in the trial if the subject:   1. Has a history of claustrophobia or inability to tolerate mock scanner environment if used during habituation/screening session. 2. Fulfills any of the MRI contraindications on the standard radiography screening questionnaire at a participating site’s imaging facility (eg, history of surgery involving metal implants, metal body piercing, dentures, dental plates or bridges, any implanted device that is electrically, magnetically, and mechanically activated). | |

| 1. Has a history in the last year or is currently receiving treatment with clozapine. 2. Has a current diagnosis of a significant psychiatric illness other than schizophrenia, per DSM-5, and is in an acute phase/episode. |
| --- |
| **Main Criteria for Evaluation and Analyses:**  The primary endpoints of the trial are:   - Change from placebo in BOLD signal in the average of left and right VS activation in the MID Reward Task at first testing after TAK-041 administration (Day 1). - Change from placebo in the BACS composite score at second testing after TAK-041 administration (Day 14). The secondary endpoints will be assessed through evaluation of the following parameters: - Percentage of subjects who experience at least 1 treatment-emergent adverse event. - Percentage of subjects who meet the markedly abnormal criteria for safety laboratory tests at least once postdose. - Percentage of subjects who meet the markedly abnormal criteria for vital sign measurements at least once postdose. - Percentage of subjects who meet the markedly abnormal criteria for safety ECG parameters at least once postdose. - Columbia-Suicide Severity Rating Scale at all time points assessed. |
| **Statistical Considerations:**  **Measures and Bayesian GO/NO-GO Decision-Making Approach at the End of the Study**  Both primary endpoints will be used for the evaluation of the effect of TAK-041 for the treatment of schizophrenia.  A Bayesian GO/NO-GO decision-making approach will be implemented for the evaluation of the effect of TAK-041 on schizophrenia. Using the observed mean (SD) difference in the 2 primary endpoints between the 2 groups  (TAK-041 and placebo), the posterior probability that TAK-041 increases the BACS composite score by 2 points over placebo, and the posterior probability that TAK-041 increases the VS activity by at least 0.09 compared to placebo, will be calculated. Noninformative prior distribution will be used. The criteria for declaring GO and NO-GO is defined in the SAP.  In addition to estimating the probability of TAK-041 meeting the effect thresholds, summary statistics on the primary, secondary, and exploratory endpoints on the original scale and/or natural log scale will be provided for TAK-041 and placebo. Analysis of covariance models for crossover design will be implemented where appropriate.  **Safety**  Safety analyses including adverse events, changes in clinical laboratory values, vital signs, ECGs, and other safety observations will be summarized by placebo and TAK-041.  **Pharmacokinetic Measures**  Descriptive statistics will be used to summarize plasma concentrations over collection time points and PK parameters of TAK-041. Individual plasma concentration and PK parameter data will be presented in data listings.  **Interim Analysis:**  A small group from the sponsor will remain unblinded to treatment assignment during the study for the purpose of conducting interim analyses of PD effects. In order to minimize any potential unblinding of investigators, these staff will not regularly interact with the investigators or their staff.  A planned, unblinded IA of various PD endpoints will be conducted when approximately 12 subjects have completed the Day 14 procedures in both periods including the primary endpoints. The purpose of the interim PD analysis will be to determine whether to change the dose of TAK-041. Any alternative dose will be supported by emerging safety, tolerability and PK data. The Bayesian decision rule will be specified in the SAP before the data are unblinded for the IA. In addition, a decision to switch to a lower dose may be made based on safety, tolerability and PK data of a higher dose. |

| **Sample Size Justification:**  Up to 32 subjects are planned to be randomized equally to 2 treatment sequences to ensure 24 subjects complete the trial.  Both primary endpoints will be used at the IA and the end of the study for decision making. A Bayesian method will be used to calculate the posterior probability of the endpoints meeting the predefined criteria. The probability of making a “GO” decision based on the predefined “GO” criteria with 24 subjects is at least 70% when the true TAK-041 effect on either endpoint is clinically meaningful. This sample size will also keep the type 1 error rate no more than 30% when the true drug effect is minimal or the same as placebo. The SD for the BACS composite score is assumed to be  9.30 points. The SD for the VS activation is assumed to be 0.360. Independence between these 2 endpoints was assumed in the sample-size calculation.  Noninformative prior distribution was used in the above probability calculation. Allowing for 8 dropouts (due to long washout interval), up to 32 subjects are planned to be enrolled in order to have 24 completed this trial. |
| --- |

- 1. **Protocol Amendment 04 Rationale for Amendment 04**

This document describes the changes in reference to the protocol incorporating Amendment No. 04.

Minor grammatical, editorial, and formatting changes are included for clarification purposes only. For specific descriptions of text changes and where the changes are located, see [Appendix E.](#_bookmark165)

## Changes in Amendment 04:

1. Modified rationale and potential range for study drug dose level.
2. Detailed potential study drug dose level change following the interim analysis.
3. Clarified Period 2 baseline serum pregnancy test collection day.
4. Revised exclusion criteria for abnormal laboratory values to clarify collection times for screening and baseline laboratory tests.
5. Revised exclusion criterion for magnetic resonance imaging contraindication before imaging assessments.
6. Detailed follow-up laboratory tests required for drug-induced liver injury (DILI).
7. Updated and further clarified excluded medications, supplements, and dietary products
8. Modified potential number of sites to approximately 4.
9. Introduced randomization stratification by site.
10. Extended screening period to 35 days prior to dosing.
11. Updated schedule of study procedures.

## 2.0 STUDY SCHEMATIC

Typical treatment, testing, and washout periods are illustrated in [Figure 2.a.](#_bookmark14) Testing day scheduled times are estimates of time required in minutes to complete the procedures.

##
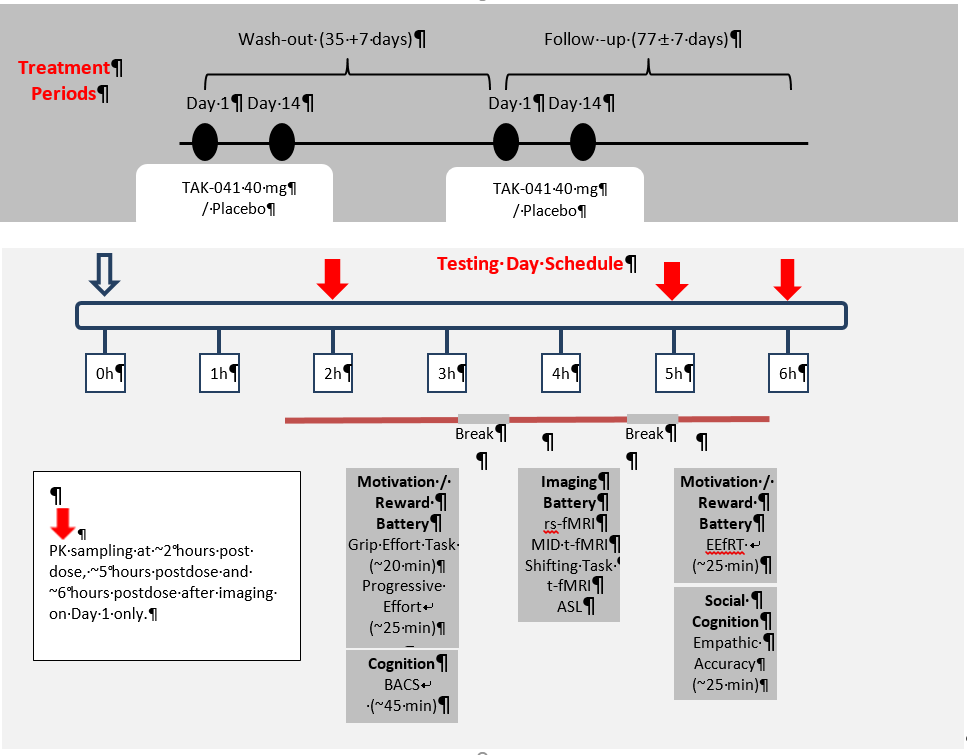
Figure 2.a TAK-041-2001 Study Schematic

ASL=arterial spin labeling, BACS=Brief Assessment of Cognition in Schizophrenia, CGI=Clinical Global Impression, EEfRT=Effort Expenditure for Rewards Task, MID=Monetary Incentive Delay, PK=pharmacokinetic, rs-fMRI=resting state functional magnetic resonance imaging, t-fMRI=2-task functional magnetic resonance imaging.

## 3.0 SCHEDULE OF STUDY PROCEDURES Table 3.a Schedule of Study Procedures

|  | | | **Schedule for Period 1(a)** | | | | | | | | | **End of Period 1 Washout Visit (c)** | **ET** |
| --- | --- | --- | --- | --- | --- | --- | --- | --- | --- | --- | --- | --- | --- |
|  |  |  | **Day -1 Baseline assessments (b)** | **Treatment Assessments** | | | | | | | **Follow-**  **up Visit** |  |  |
| **Study Days** | **Days**  **-35 to -2** | |  | **Dosing and PD Testing Day 1** | | | | | | | **Day 14** | **35 +7 days**  **postdose** |  |
|  |  | |  | **Hours** | | | | | | |  |  |  |
|  |  | |  | **Predose** | **1** | | **2** | **3.5** | **5** | **6** |  |  |  |
| **Administrative procedures** | | | | | | | | | | | | | |
| Informed consent (retained  samples, PGx) | X | |  |  |  |  | |  |  |  |  |  |  |
| Inclusion/exclusion criteria | X | | X | X |  |  | |  |  |  |  |  |  |
| Medical history/demographics | X | |  |  |  |  | |  |  |  |  |  |  |
| Prior and concomitant  medication review | Continuous Review | | | | | | | | | | | | |
| **Clinical Procedures/Assessments** | | | | | | | | | | | | | |
| Full physical examination | | X |  | X (d) |  |  | |  |  |  |  |  |  |
| Psychiatric examination | | X |  |  |  |  | |  |  |  |  |  |  |
| Height | | X |  |  |  |  | |  |  |  |  |  |  |
| Weight | | X |  |  |  |  | |  |  |  |  |  |  |
| BMI | | X |  |  |  |  | |  |  |  |  |  |  |
| Semirecumbent vital signs (HR,  SBP, and DBP) | | X |  | X (e) |  |  | |  |  |  | X | X | X |

Footnotes are on last table page.

|  | | **Schedule for Period 1 (a)** | | | | | | | | | **End of Period 1 Washout Visit (c)** | **ET** |
| --- | --- | --- | --- | --- | --- | --- | --- | --- | --- | --- | --- | --- |
|  |  | **Day -1 Baseline assessments (b)** | **Treatment Assessments** | | | | | | | **Follow- up Visit** |  |  |
| **Study Days** | **Days**  **-35 to -2** |  | **Dosing and PD Testing Day 1** | | | | | | | **Day 14** | **35 +7 days postdose** |  |
|  |  |  | **Hours** | | | | | | |  |  |  |
|  |  |  | **Predose** | **1** | **2** | **3.5** | **5** | **6** | |  |  |  |
| Vital signs (respiratory rate, oral [floor of the mouth]/ tympanic  temperature) | X |  | X (e) |  |  |  |  |  | | X | X | X |
| Standard 12-lead ECG | X |  | X (e) |  |  |  |  |  | | X | X | X |
| TAK-041/placebo  administration (f) |  |  | X  (0 hour) |  |  |  |  |  | |  |  |  |
| MINI | X |  |  |  |  |  |  |  | |  |  |  |
| PANSS | X | X |  |  |  |  |  |  | | X |  | X |
| BNSS | X | X |  |  |  |  |  |  | | X | X | X |
| CGI-S | X | X |  |  |  |  |  |  | | X | X | X |
| CGI-I |  |  |  |  |  |  |  |  | | X | X | X |
| C-SSRS (g) | X | X |  |  |  |  |  |  | | X | X | X |
| Structural MRI (h) | X |  |  |  |  |  |  |  | |  |  |  |
| AE monitoring | Continuous Monitoring | | | | | | | | | | | X |
| **Laboratory Procedures/Assessments** | | | | | | | | | | | | |
| Hematology | X (i) | X (i) |  |  |  |  |  |  | X | |  | X |
| Urinalysis | X (i) | X (i) |  |  |  |  |  |  | X | |  | X |
| Serum chemistry | X (i) | X (i) |  |  |  |  |  |  | X | |  | X |
| Serum FSH | X |  |  |  |  |  |  |  |  | |  |  |

Footnotes are on last table page.

|  | | **Schedule for Period 1 (a)** | | | | | | | | **End of Period 1 Washout Visit (c)** | **ET** |
| --- | --- | --- | --- | --- | --- | --- | --- | --- | --- | --- | --- |
|  |  | **Day -1 Baseline assessments (b)** | **Treatment Assessments** | | | | | | **Follow- up Visits** |  |  |
| **Study Days** | **Days**  **-35 to**  **-2** |  | **Dosing and PD Testing Day 1** | | | | | | **Day 14** | **35 +7 days postdose** |  |
|  |  |  | **Hours** | | | | | |  |  |  |
|  |  |  | **Predose** | **1** | **2** | **3.5** | **5** | **6** |  |  |  |
| Urine drug screen (j) | X (i) | X (i) | X |  |  |  |  |  | X |  |  |
| Alcohol breath test (j) | X |  | X |  |  |  |  |  | X |  |  |
| Serum pregnancy test (c) | X | X |  |  |  |  |  |  | X | X | X |
| Hepatitis panel (including HBsAg and anti-HCV), HIV, TSH | X |  |  |  |  |  |  |  |  |  |  |
| 5'-nucleotidase | X | X |  |  |  |  |  |  | X |  | X (k) |
| **PK Evaluations** | | | | | | | | | | | |
| Plasma sample for PK (k) |  |  | X | X | X |  | X | X | X | X | X (k) |
| **PD Evaluations** | | | | | | | | | | | |
| BACS |  | X(l) |  |  | X |  |  |  | X |  |  |
| Social Cognition |  | X (l) |  |  |  |  | X |  | X |  |  |
| Motivation/Reward Battery |  | X (l) |  |  | X |  | X |  | X |  |  |
| Imaging Battery |  | X (m) |  |  |  | X |  |  | X |  |  |

Footnotes are on last table page.

|  | | **Schedule for Period 1 (a)** | | | | | | | | **End of Period 1 Washout Visit (c)** | **ET** |
| --- | --- | --- | --- | --- | --- | --- | --- | --- | --- | --- | --- |
|  |  | **Day -1 Baseline assessments (b)** | **Treatment Assessments** | | | | | | **Follow- up Visits** |  |  |
| **Study Days** | **Days**  **-35 to -2** |  | **Dosing and PD Testing Day 1** | | | | | | **Day 14** | **35 +7 days postdose** |  |
|  |  |  | **Hours** | | | | | |  |  |  |
|  |  |  | **Predose** | **1** | **2** | **3.5** | **5** | **6** |  |  |  |
| **PGx Evaluations** | | | | | | | | | | | |
| Blood sample for DNA (n) |  |  | X |  |  |  |  |  |  |  |  |
| Blood sample for RNA |  |  | X |  |  |  |  |  | X |  |  |
| **Other** | | | | | | | | | | | |
| Tobacco and caffeine consumption (o) |  | X | X |  |  |  |  |  | X | X |  |
| Wearable devices (p) |  |  |  |  |  |  |  | X X | |  |  |

Footnotes are on last table page.

| **Study Days** | | **Schedule for Period 2 (a)** | | | | | | | | | **End of Period 2 Study Exit** | **ET** |
| --- | --- | --- | --- | --- | --- | --- | --- | --- | --- | --- | --- | --- |
|  |  | **Day -1**  **Period 2 Baseline**  **assessments (b)** | **Treatment Assessments** | | | | | | **Period 2 Follow-up Visits** | |  |  |
|  |  |  | **Dosing and PD Testing Day 1** | | | | | | **Day 14** | **Day 49±4**  **days post dose** | **77 ±7 days post dose** |  |
|  | |  | **Hours** | | | | | |  |  |  |  |
|  | |  | **Predose** | **1** | **2** | **3.5** | **5** | **6** |  |  |  |  |
|  | **Administrative procedures** | | | | | | | | | | | |
| Informed consent (retained samples,  PGx) | |  |  |  |  |  |  |  |  |  |  |  |
| Inclusion/exclusion criteria | | X |  |  |  |  |  |  |  |  |  |  |
| Medical history/demographics | |  |  |  |  |  |  |  |  |  |  |  |
| Prior and concomitant medication  review | |  | --------------------------------------------------Continuous  Review----------------------------------------------------------------- | | | | | | | | | |
|  | **Clinical Procedures/Assessments** | | | | | | | | | | | |
| Full physical examination | |  | X (d) |  |  |  |  |  |  |  |  |  |
| Psychiatric examination | |  |  |  |  |  |  |  |  |  |  |  |
| Height | |  |  |  |  |  |  |  |  |  |  |  |
| Weight | |  |  |  |  |  |  |  |  |  |  |  |
| BMI | |  |  |  |  |  |  |  |  |  |  |  |
| Semirecumbent vital signs (HR, SBP,  and DBP) | |  | X (e) |  |  |  |  |  | X | X | X | X |

Footnotes are on last table page.

| **Study Days** | | **Schedule for Period 2 (a)** | | | | | | | | | | **End of Period 2 Study Exit**  **(c)** | **ET** |
| --- | --- | --- | --- | --- | --- | --- | --- | --- | --- | --- | --- | --- | --- |
|  |  | **Period 2 Day-1 Baseline assessmen**  **ts (b)** | **Treatment Assessments** | | | | | | | **Follow-up Visits** | |  |  |
|  |  |  | **Dosing and PD Testing Day 1** | | | | | | | **Day 14** | **Day 49±4**  **days postdose** | **77 ± 7 days postdose** |  |
|  | |  | **Hours** | | | | | | |  |  |  |  |
|  |  |  | **Predose** | **1** | **2** | **3.5** | **5** | **6** | |  |  |  |  |
| Vital signs (respiratory rate, oral [floor  of the mouth]/ tympanic temperature) | |  | X (e) |  |  |  |  |  | | X | X | X | X |
| Standard 12-lead ECG | |  | X (e) |  |  |  |  |  | | X | X | X | X |
| TAK-041/placebo administration (f) | |  | X  (0 hour) |  |  |  |  |  | |  |  |  |  |
| MINI | |  |  |  |  |  |  |  | |  |  |  |  |
| PANSS | | X |  |  |  |  |  |  | | X | X |  | X |
| BNSS | | X |  |  |  |  |  |  | | X | X | X | X |
| CGI-S | | X |  |  |  |  |  |  | | X | X | X | X |
| CGI-I | |  |  |  |  |  |  |  | | X | X | X | X |
| C-SSRS (g) | | X |  |  |  |  |  |  | | X |  | X | X |
| Structural MRI (h) | |  |  |  |  |  |  |  | |  |  |  |  |
| AE monitoring | |  | --------------------------------------------------Continuous Monitoring-------------------------------- | | | | | | | | | | X |
|  | **Laboratory Procedures/Assessments** | | | | | | | | | | | | |
| Hematology | | X (i) |  |  |  |  |  |  | X | | X |  | X |
| Urinalysis | | X (i) |  |  |  |  |  |  | X | | X |  | X |
| Serum chemistry | | X (i) |  |  |  |  |  |  | X | | X |  | X |

Footnotes are on last table page.

| **Study Days** | | **Schedule for Period 2 (a)** | | | | | | | | | **End of Period 2 Study Exit (c)** | **ET** |
| --- | --- | --- | --- | --- | --- | --- | --- | --- | --- | --- | --- | --- |
|  |  | **Day -1 Period**  **2 Baseline assessments (b)** | **Treatment Assessments** | | | | | | **Follow-up Visits** | |  |  |
|  |  |  | **Dosing and PD Testing Day 1** | | | | | | **Day 14** | **Day 49±4**  **days postdose** | **77 ±7 days postdose** |  |
|  | |  | **Hours** | | | | | |  |  |  |  |
|  | |  | **Predose** | **1** | **2** | **3.5** | **5** | **6** |  |  |  |  |
| Urine drug screen (j) | | X (i) | X |  |  |  |  |  | X |  |  |  |
| Alcohol breath test (j) | |  | X |  |  |  |  |  | X |  |  |  |
| Serum pregnancy test (c) | | X |  |  |  |  |  |  | X | X | X | X |
| Hepatitis panel (including HBsAg and anti-HCV), HIV, TSH | |  |  |  |  |  |  |  |  |  |  |  |
| 5'-nucleotidase | | X |  |  |  |  |  |  | X | X |  | X (k) |
|  | **PK Evaluations** | | | | | | | | | | | |
| Plasma sample for PK (k) | |  | X | X | X |  | X | X | X | X | X | X (k) |
|  | **PD Evaluations** | | | | | | | | | | | |
| BACS | | X(l) |  |  | X |  |  |  | X |  |  |  |
| Social Cognition | | X (l) |  |  |  |  | X |  | X |  |  |  |
| Motivation/Reward Battery | | X (l) |  |  | X |  | X |  | X |  |  |  |
| Imaging Battery | | X (m) |  |  |  | X |  |  | X |  |  |  |

Footnotes are on last table page.

| **Study Days** | | **Schedule for Period 2 (a)** | | | | | | | | | **End of Period 2 Study Exit (c)** | **ET** |
| --- | --- | --- | --- | --- | --- | --- | --- | --- | --- | --- | --- | --- |
|  |  | **Day -1**  **Period 2 Baseline assessments (b)** | **Treatment Assessments** | | | | | | **Follow-up Visits** | |  |  |
|  |  |  | **Dosing and PD Testing Day 1** | | | | | | **Day 14**  **(Period 2)** | **Day 49±4**  **days postdose** | **77 ± 7**  **days postdose** |  |
|  | |  | **Hours** | | | | | |  |  |  |  |
|  | |  | **Predose** | **1** | **2** | **3.5** | **5** | **6** |  |  |  |  |
|  | **PGx Evaluations** | | | | | | | | | | | |
| Blood sample for DNA (n) | |  |  |  |  |  |  |  |  |  |  |  |
| Blood sample for RNA | |  | X |  |  |  |  |  | X |  |  |  |
|  | Other | | | | | | | | | | | |
| Tobacco and caffeine consumption (o) | | X | X |  |  |  |  |  | X |  | X |  |
| Wearable devices (p) | |  |  |  |  |  |  | X X | |  |  |  |

AE=adverse event, BACS=Brief Assessment of Cognition in Schizophrenia, BMI=body mass index, BNSS=Brief Negative Symptom Scale, CGI-I=Clinical Global Impression - Improvement, CGI-S=Clinical Global Impression - Severity, C-SSRS=Columbia-Suicide Severity Rating Scale, DBP=diastolic blood pressure, ECG=electrocardiogram, ET=Early Termination, FSH=follicle-stimulating hormone, HBsAg=hepatitis B surface antigen, HCV=hepatitis C virus, HIV=human immunodeficiency virus, HR=heart rate, MINI=Mini International Neuropsychiatric Interview, MRI=magnetic resonance imaging, PANSS=Positive and Negative Syndrome Scale, PD=pharmacodynamic, PGx=pharmacogenomics, PK=pharmacokinetics, SBP=systolic blood pressure, TSH=thyrotropin.

1. The schedule for Periods 1 and 2 is identical from Day -1 to Day 14.
2. Baseline PD assessments must be completed within 24 hours before Day 1 dosing and PD testing in both Periods. PD assessments and testing take priority over all other procedures. If P1 Day 35 and P2 Day-1 are the same day, assessments required for both visits only need to be completed once. PD testing will be performed in 3 blocks, separated by two breaks, [Figure 2.a.](#_bookmark14)
3. The sites will schedule a visit for subjects at the end of the Period 1 Washout. Subjects may return to the sites for this visit any day between Day 35 and 42. This Visit will serve to conduct end of Period 1 procedures *and* may also be the same calendar day as Day -1 Baseline assessments for Period 2. If P1 Day 35 and P2 Day 1 are the same day, assessments required for both visits only need to be completed once. Subjects will return to the clinic the next day for Period 2 dosing and PD testing.
4. Predose physical may be done within approximately 24 hours predose.
5. Vital signs and a 12-lead ECG will be performed within approximately 1 hour predose on Day 1 in both Periods.
6. Subjects will be administered study drug at the clinic on Day 1 of Periods 1 and 2.
7. Two versions of the C-SSRS will be used in this trial: the Screening/Baseline C-SSRS Lifetime Version 14Jan2009 and the Since-Last-Visit C-SSRS Version 14Jan2009. C-SSRS only needs to be captured once if P1 Day 35 and P2 D-1 are the same day.
8. If not conducted within the preceding year, a structural MRI may be performed during screening to ensure the absence of structural brain abnormalities that might interfere with interpretation of functional brain imaging results.
9. Must be reviewed by the investigator before dosing on Day 1 in both Periods.
10. May be repeated within 28 days of screening per investigator’s discretion.
11. Collect a blood sample if subject early terminates before Day 49.
12. Subjects will complete practice sessions **as well as** baseline measurements in Period 1 only.
13. Familiarization with tasks ONLY in Period 1. No imaging data collected here.
14. DNA is collected in Period 1 ONLY.
15. Subjects will be questioned if they have used tobacco- or caffeine-containing products on the day prior to baseline assessments and testing.
16. For subjects who consent to participate in the wearable device optional component, the devices will be deployed when PK assessments are over on Day 1 (both Treatment Periods) and worn continuously until the subjects return to the sites for Day 14 testing. The details of data collection by wearable devices will be provided in the trial manual.

## INTRODUCTION

## Background

G protein-coupled receptor 139 (GPR139) is an orphan G protein-coupled receptor identified as a target mainly expressed in the dorsal medial habenula. The habenula is part of the diencephalon, which along with the pineal gland makes up the “epithalamus.” The pineal gland is attached to the diencephalon by a stalk, with 2 (bilateral) small nuclei at the base that are the habenulae. This is a highly conserved and crucial brain region that facilitates functional interactions among the limbic system, basal ganglia, ventral tegmental area, and midbrain raphe nuclei. The habenula has been shown to be involved in appropriate social interactions, learning behavior, and cognition [[1,](#_bookmark143)[2](#_bookmark144)]. In monozygotic twins discordant for schizophrenia, copy number variants of GPR139 have been reported in the affected twin [[3](#_bookmark145)]. In GPR139 -/- knockout mice, impairments in tasks associated with motivation and social behavior are observed. Taken together, these results suggest that GPR139 plays an integral part in regulating medial habenular function and thereby may be important in the regulation of social and other behaviors that are impaired in people with schizophrenia.

TAK-041 is a highly selective and potent agonist of GPR139. In numerous preclinical studies, TAK-041 has demonstrated potential efficacy in animal models of amotivation, anhedonia, and asociality, which are behaviors classified as “negative symptoms of schizophrenia.” Negative symptoms include difficulties with expressive behaviors (blunted affect, poverty of speech) as well as impairments in emotional behaviors (amotivation, asociality, anhedonia). Impairments in social behavior are common, with shared characteristics across a host of other psychiatric conditions, including autism spectrum disorders, attention-deficit/hyperactivity disorder, and major depressive disorder.

## Rationale for the Proposed Study

To date there are no approved drugs addressing negative symptoms or cognitive impairment associated with schizophrenia. Since motivational deficits and anhedonia are core components of negative symptoms, recent research has focused on developing translational tasks in these domains in order to support development of new pharmacological interventions for negative symptoms.

Based on the unique target expression profile of GPR139 in the habenula, data obtained from GPR139 -/- knockout mice, as well as efficacy of TAK-041 in multiple preclinical models of anhedonia, the current trial is designed to evaluate the effects of TAK-041 on motivational anhedonia and cognitive function using motivation/reward battery tests, as well as effects on brain imaging using functional magnetic resonance imaging (fMRI) Blood-Oxygen-Level-Dependent (BOLD) and arterial spin labeling (ASL), in subjects with stable schizophrenia, as an add-on to stable antipsychotic treatment.

## Benefit/Risk Profile

TAK-041 has the potential to be a first-in-class drug; therefore, there are no known class effects. Potential risks are based on clinical findings, the mechanism of action, nonclinical findings, and the known risks of other GPR139 receptor agonists.

Preliminary blinded results from the TAK-041-1001 study indicate that single doses of 5, 10, 20, 40, 80, 120 and 160 mg and multiple dose cohorts 1 (40mg/20mg) and 2 (80 mg/40 mg) consisting of 4 weekly doses, 1 loading dose followed by weekly maintenance doses for 3 weeks are well tolerated. In the TAK-041-1001 study, 31 AEs were observed that were all mild and resolved without medication treatment. Thirty of the AEs were deemed not related to study drug, and resolved without treatment. For the 66 subjects in the above cohorts there were no serious AEs, no clinically meaningful abnormalities in safety laboratory results (including ALP, AST, ALT, GGT, and TBILI) and no clinically significant abnormalities in physical examination, vital signs, or ECG results reported.

The potential risks can be monitored clinically and/or with laboratory tests and were considered in setting up the stopping rules for this clinical trial. Appropriate eligibility criteria that exclude individuals with past history or concurrent conditions that increase the risk have been applied.

## STUDY OBJECTIVES AND ENDPOINTS

## Objectives

## Primary Objective

- To determine whether motivation/reward deficits observed in schizophrenia are attenuated by add-on TAK-041 administration to antipsychotics in subjects with stable schizophrenia.
- To determine whether cognitive impairment associated with schizophrenia is improved by add-on TAK-041 administration to antipsychotics in subjects with stable schizophrenia.

## Primary Hypotheses:

- TAK-041 demonstrates greater increase compared with placebo in BOLD signal in the average of left and right ventral striatum (VS) activation in the Monetary Incentive Delay (MID) Reward Task (high posterior probability of [a difference between TAK-041 and placebo

>0.09] is expected).

- TAK-041 demonstrates greater increase than placebo in the BACS composite score (high posterior probability of [a difference between TAK-041 and placebo >2.0] is expected).

## Secondary Objectives

- To determine safety and tolerability of TAK-041 as add-on therapy to antipsychotics in subjects with stable schizophrenia.

## Exploratory Objectives

- To explore the relationship between exposure and pharmacodynamic (PD) effects (ie, performance tests) of TAK-041.
- To determine whether social cognition impairment observed in schizophrenia is improved by add-on TAK-041 administration to antipsychotics in subjects with stable schizophrenia.
- To explore potential genetic and transcriptome contributions to possible TAK-041 outcome in performance tests and to contribute to disease target discovery efforts.
- Optional: To assess the effect of TAK-041 administration on device-derived biometric parameters such as mobility and sleep.

## Endpoints

## Primary Endpoints

- Change from placebo in BOLD signal in the average of left and right VS activation in the MID Reward Task at first testing after TAK-041 administration (Day 1).
- Change from placebo in the BACS composite score at second testing after TAK-041 administration (Day 14).

## Secondary Endpoints

- Percentage of subjects who experience at least 1 treatment-emergent adverse event (TEAE).
- Percentage of subjects who meet the markedly abnormal criteria for safety laboratory tests at least once postdose.
- Percentage of subjects who meet the markedly abnormal criteria for vital sign measurements at least once postdose.
- Percentage of subjects who meet the markedly abnormal criteria for safety ECG parameters at least once postdose.
- Columbia-Suicide Severity Rating Scale (C-SSRS) at all time points assessed.

## Exploratory Endpoints

1. Area under the plasma concentration-time curve (AUC) and maximum observed plasma concentration (C_max_) of TAK-041.
2. Change from placebo in BOLD signal in ventro-lateral prefrontal cortex activation in Set-Shifting Task at first and second testing after TAK-041 administration.
3. Change from placebo in functional connectivity using rs-fMRI at first and second testing after TAK-041 administration.
4. Change from placebo in whole brain regional cerebral blood flow measured using ASL.
5. Change from placebo in the maximum level of physical effort a subject is willing to exert to obtain rewards at different reward levels in the Progressive Ratio Task at first and second testing after TAK-041 administration.
6. Change from placebo in the natural log-transformed % of hard choices at the highest reward level from the Grip Effort Task at second testing after TAK-041 administration.
7. Change in BOLD signal compared to placebo in the average of left and right VS activation in the MID Reward Task at second testing after TAK-041 administration.
8. Change from placebo in the natural log transformed % of hard choices at the highest reward level from the Effort Expenditure for Rewards Task (EEfRT) at second testing after TAK-041 administration.
9. Change from placebo in the BACS composite score at second testing after TAK-041 administration.
10. Change from placebo in Positive and Negative Syndrome Scale (PANSS) total score, subscales, and factors at all time points tested after TAK-041 administration.
11. Change from placebo in Brief Negative Symptom Scale (BNSS) total score at all time points tested after TAK-041 administration.
12. Change from placebo in Clinical Global Impression (CGI) scores at all time points tested after TAK-041 administration.
13. Pharmacogenomics (PGx) analysis of DNA and RNA in blood samples.
14. Measurements of device-derived biometric parameters, such as subject’s activity and sleep after TAK-041 administration.
15. Optional: Change from placebo in the natural log-transformed % of hard choices at the highest reward probability level of the EEfRT at first testing after TAK-041 administration (Day 1).
16. Optional: Change from placebo in Empathic Accuracy Task at all time points tested after TAK-041 administration.

## STUDY DESIGN AND DESCRIPTION

## Study Design

This study is a randomized, double-blind, placebo-controlled, 2-period, crossover phase 2 study to evaluate the pharmacodynamic (PD) effects, safety, tolerability, and PK of single doses of oral TAK-041 in adult subjects with schizophrenia, particularly negative symptoms characterized by reduced motivation. Effects of at least 1 dose level and placebo will be assessed. The study is designed to evaluate the effect of TAK-041, a GPR139 orphan receptor agonist, as an add-on to antipsychotics in attenuating the impairment in motivational anhedonia as well as cognitive function using motivation/reward battery tests as well as brain imaging (ie, task-induced fMRI BOLD and ASL) in subjects with stable schizophrenia. Subjects should be stable on their existing medicine (ie, second-generation antipsychotics [SGAs]) for at least 2 months prior to screening. The initial dose level selected will be 40 mg but may be adjusted based on emerging safety, tolerability and PK data. A planned unblinded interim analysis (IA) for efficacy may be conducted when approximately 12 subjects have completed the Day 14 procedures in both periods. The IA objective is to determine whether to continue the current TAK-041 dose after the IA, or revise to a different dose.

The study will consist of 2 treatment periods, with a single dose of study drug administered in each period. There will be a 35 day (+ 7 days) washout interval between the 2 doses to reduce the potential for residual TAK-041 to impact the PD endpoints. Treatment Period 2 begins at the end of the Treatment Period 1 washout. Because subjects are not confined in this study, they will come to the clinic for the following visits:

- Screening Visit (between Days -35 to -2) covering full medical and psychiatric examinations.
- Baseline Assessments Visit (Day -1 in each Treatment Period), covering motivation/reward and cognitive testing baseline, except fMRI.
- Dosing and First Testing Visit (Day 1 in each Treatment Period).
- Second Testing Visit (Day 14 in each Treatment Period).
- Follow-up Visit (Day 49 [±4 days] post dose for Treatment Period 2 only).
- End of Period 1 Visit (Day 35 [+7 days] postdose in Period 1). This Visit will serve to conduct end of Period 1 procedures and the Day -1 Baseline assessments for Period 2.
- Study Exit occurs at the Final Visit 77 ±7 days post–Period 2 dose.

The trial population will include subjects with stable schizophrenia aged 18-60 years, inclusive, considered eligible on the basis of the trial inclusion and exclusion criteria. The trial will randomize up to 32 subjects to ensure 24 subjects complete. On Day 1 of Period 1, eligible subjects will be randomized in a ratio of 1:1 to 1 of the 2 treatment sequences ([Table 6.a](#_bookmark32)) and will receive each study drug according to the randomized sequence group. The randomization will be stratified by the sites. The initial dose of TAK-041 will be 40 mg and may be adjusted prior to the IA depending on available PK and safety data from emerging first-in-human cohorts. The dose for

TAK-041 may also be changed for subjects enrolled after the IA. The decision criteria will be predefined in the SAP before unblinding.

## Table 6.a Sequence Groups

**Sequence Period 1 Period 2**

1 TAK-041 +

antipsychotic

2 Placebo +

antipsychotic

Placebo + antipsychotic

TAK-041 +

antipsychotic

During the Screening Visit (Days -35 to -2), subjects meeting Diagnostic and Statistical Manual of Mental Disorders fifth edition (DSM-5) criteria for schizophrenia [[4](#_bookmark146)] and on stable antipsychotic therapy for at least 2 months will complete medical and psychiatric examinations, electrocardiogram (ECG), and laboratory safety tests. At the Baseline Assessments Visit (Day -1) for Period 1, subjects will complete practice sessions of the motivation/reward and cognitive battery in order to minimize potential practice effects at subsequent treatment visits, and be familiarized to the magnetic resonance imaging (MRI) tasks. At both study periods, subjects will also be tested for Day -1 baseline assessments (except fMRI). Practice sessions will be carried out prior to the baseline assessments in Period 1 only. Subjects meeting all inclusion criteria and no exclusion criteria will be administered study medication or placebo in the clinic based on the randomization schedule on Day 1 of Treatment Periods 1 and 2.

On Day 1 of each Treatment Period, following study drug administration, and commencing at approximately 2 hours postdose, subjects will undergo PD tests for a total of approximately

4 hours. Blood samples will be collected at defined times after dosing to assess the PK of

TAK-041. After the last PK collection at 6 hours postdose, subjects will leave the clinic. On Day 14 of each Treatment Period, subjects will take the second set of PD tests along with a single time point PK collection. Samples for PK will also be collected on Day 49 and Final Visit of second Treatment Period. There will be a washout period of 35 days (+ 7 days) between doses in each Treatment Period to reduce the potential for residual TAK-041 to impact the PD endpoints.

PD testing will be performed in 3 blocks, separated by two breaks. The test schedule is detailed in the Schedule of Study Procedures, [Table 3.a](#_bookmark16). The first block will comprise the Grip Effort Task, Progressive Ratio Test, and BACS. After a break, subjects will take the second block that comprises a battery of imaging tests; namely, brain perfusion using non-contrast ASL MRI and fMRI scans which monitor changes in BOLD signal. The fMRI will be run in resting state without a task (rs-fMRI) and with 2-task functional magnetic resonance imaging (t-fMRI) using the MID Reward Task and a Set-Shifting Task. After a break, subjects will take the third block which comprises the EEfRT and the Empathic Accuracy Task. Subjects will also receive the PANSS, the BNSS, and the CGI Scales (CGI-Severity [CGI-S] and CGI-Improvement [CGI-I]) as a part of the PD assessment. Subject’s activity and sleep rhythms will also be captured with wearable digital devices after TAK-041 or placebo administration. Note that the EEfRT and Empathic Accuracy tasks, as well the wearable devices, are designated as optional assessments due to the need to demonstrate the operational feasibility of conducting these tasks and devices at the research sites.

These tasks will only be included if the feasibility and validity of data generated by the tasks are confirmed. If included in the study procedures, these tasks will be conducted only in participants randomized to study treatment after the feasibility of the task (or tasks) and devices have been demonstrated.

Blood will be drawn for PK analysis at defined days and times during the treatment period, follow-up and final visit or at early termination if it occurs, as detailed in the Schedule of Study Procedures, [Table 3.a.](#_bookmark16)

PGx assessments will be performed from the blood samples collected on Day 1 (prior to TAK-041 or placebo administration) for DNA in Period 1 only, and in both Treatment Periods for RNA on Day 1 (prior to TAK-041 or placebo administration) and on Day 14.

Typical treatment, testing, and washout periods are illustrated in [Figure 2.a](#_bookmark14).

## Rationale for Study Design, Dose, and Endpoints

## Rationale of Study Design and Regimen

Based on the target expression profile and preclinical data obtained from GPR139 -/- knockout mice as well as from preclinical data for TAK-041, the current trial is designed to evaluate the effect of TAK-041 in attenuating impairment in motivational anhedonia, cognitive function using motivation/reward battery tests, as well as task-based fMRI brain imaging using BOLD in subjects with stable schizophrenia.

To date there are no approved compounds addressing negative symptoms as well as cognitive impairment associated with schizophrenia. Since motivational deficits and anhedonia are core components of negative symptoms, recent research has focused on developing translational tasks in these domains in order to support development of new pharmacological interventions for negative symptoms in subjects with schizophrenia.

The crossover design has been selected in order to reduce sample size (N = 24), as well as minimize variability in the endpoints. TAK-041 has been evaluated in a single-rising dose (SRD) and multiple rising dose (MRD) trial in healthy volunteers with single doses up to 160 mg and multiple doses regimen 40/20 mg (loading dose on week 1/followed by 3 weekly maintenances doses) of a crystalline suspension. During the SRD trial, PK blood samples were collected for

43 days following a single TAK-041 dose. Preliminary PK data indicated that TAK-041 has a mean terminal elimination half-life (t_1/2_) of approximately 11 days. Therefore, 35 + 7 days between the 2 treatment periods is sufficient to ensure that there is no drug carryover effect. At the end of 35 +7 days, the probability of exceeding the “effect” cut-off is less than 2%, with

17 subjects out of 1000 expected to have plasma concentrations of TAK-041 above 132.5 ng/mL.

## Rationale for Dose

The initial dose examined in this study will be a single 40 mg dose of TAK-041 crystalline suspension (or matching placebo) administered on Day 1 of each treatment period according to the sequence group assigned. A 40 mg dose of TAK-041 has been administered to healthy volunteers

in the first in human study, TAK-041-1001, and was found to be safe and well-tolerated. During the study, 6 AEs were observed that were mild in nature, were deemed not related to study drug, and resolved without treatment. No SAEs and no clinically meaningful changes in safety laboratory results, physical examinations, vital signs, or ECGs were reported for the 16 subjects who received a single dose of TAK-041 crystalline suspension up to 40 mg or matching placebo.

Dose adjustment will be allowed based on emerging safety, tolerability, and PK data from this study and study TAK-041-1001. Overall, the proposed dose range of TAK-041 is anticipated to achieve sufficient range of plasma exposures with a margin above and beyond the predicted pharmacologically active exposures/concentrations established in preclinical models. Based on the IA results, adjustment to higher doses may be performed to allow for the exploration of the

dose-response.

The clinical exposure achieved at the 40 mg dose is aligned with predicted human exposure of the highest efficacious dose (1 mg/kg) tested in nonclinical in vivo models of anhedonia and asociality. The maximum efficacious dose preclinically observed was 3 mg/kg in the ASST [(attention set shifting task) which measures executive function, applicable to cognitive impairment associated with schizophrenia (CIAS) and negative symptoms of schizophrenia], and 5 mg/kg on the Nicotine and Amphetamine dopamine release on the nucleus accumbens.

TAK-041 continues to be evaluated in the ongoing SRD/MRD study (Study TAK-041-1001), and the safety and exposure data for additional single and multiple doses will be evaluated.

Preliminary blinded results from the TAK-041-1001 study indicate that single doses of 5, 10, 20, 40, 80,120, and 160 mg and multiple dose cohorts 1 (40 mg/20 mg) and 2 (80 mg/40 mg) consisting of 4 weekly doses, 1 loading dose followed by weekly maintenance doses for 3 weeks are well tolerated. In the TAK-041-1001 study, 31 AEs were observed that were all mild and resolved without treatment. For the 66 subjects in the above cohorts, there were no serious AEs, no clinically meaningful abnormalities in safety laboratory results (including ALP, AST, ALT, GGT, and TBILI) and no clinically significant abnormalities in physical examination, vital signs, or ECG results reported.

The highest planned dose will not exceed the highest dose (target range 40-160 mg) deemed safe, and well tolerated in healthy subjects. Dose escalation documents may be provided separately to the principal investigator and ethics as appropriate to support any dose change and will not exceed 160 mg.

## Rationale for Endpoints

- - - 1. *PK Endpoints*

The PK endpoints selected for this study, AUCs and C_max_ of TAK-041, are commonly used PK parameters to measure the exposure to the study drug following a single-dose administration in clinical studies.

- - - 1. *PD Endpoints*

Based on the fact that patients with schizophrenia often have difficulty with anticipatory pleasure and recruiting efforts to pursue rewards, a number of functional tasks have been developed recently to study avolition and anhedonia as PD endpoints in this patient population [[5-9](#_bookmark147)]. Using these tasks, it has been reported that patients with schizophrenia demonstrate reduced effort allocation or impaired effort-cost computations compared to healthy controls [[5-9](#_bookmark147)]. In order to assess motivation/reward in patients with schizophrenia, a specific test battery containing both performance tests and task-induced fMRI BOLD assessments has been chosen [[5-9](#_bookmark147)]. Patients with schizophrenia also show impairments in executive functions including cognitive flexibility [[10](#_bookmark150)] and this will be assessed using the set-shifting task [[11](#_bookmark151)].

*Primary Endpoints*

MID Task-induced fMRI BOLD signal: the MID task is a well-established reward anticipation paradigm that is known for robustly engaging the VS, a key area associated with coding incentive reward. Dysfunctional processing of reward information has been associated with motivational impairments in schizophrenia. Motivational impairment is a key aspect of negative symptoms, and has been associated with reduced activity in the VS.

The MID Reward Task-induced fMRI BOLD signal also has fair to good test-retest reliability (ie, ICC value 0.62) as well as moderate effect size (ie, *d* value 0.56) [[9](#_bookmark149)].

BACS: This is a reliable and sensitive measure of cognitive function in schizophrenia [[12](#_bookmark152)]. The BACS version in this study will assesses 4 domains of cognitive function found to be consistently impaired in schizophrenia: verbal and visual memory, verbal fluency, and processing speed. The set of tests is of approximately 25 minutes duration and may be conducted using either paper and pencil or a comparable version delivered electronically [[13](#_bookmark153)].

*Secondary Endpoints*

During the SRD trial, in order to fully assess the safety and PK profile of TAK-041 in subjects receiving up to 40 mg TAK-041 doses, follow-up PK blood draws were performed until TAK-041 plasma concentrations were below the limit of quantitation (<1 ng/mL). Six AEs were observed that were mild in nature, were deemed not related to study drug, and resolved without treatment. No SAEs and no clinically meaningful changes in safety laboratory results, physical examinations, vital signs, or ECGs were reported for the 16 subjects who received a single dose of TAK-041 crystalline suspension up to 40 mg or matching placebo. Therefore, standard safety endpoints and monitoring have been included in the current trial.

*Exploratory Endpoints*

Grip Effort Task: This task has been implemented in fMRI and behavioral studies, consistently showing it is a sensitive measure of behavioral effort-expenditure decision-making [[6](#_bookmark148)]. The task employs a commercially available grip force fiber optic response device capturing grip force in real time. Based on the difficulty of the task, subjects grip the handgrip with different strength levels, which correlate with different amounts of monetary reward. Grip Effort Task demonstrates

fair to good test-retest reliability (ie, intra-class correlation [ICC] value 0.63 [[6](#_bookmark148)]) as well as moderate effect size (ie, *d* value 0.34 [[6](#_bookmark148)]).

Progressive Ratio Task: In this task subjects are presented with 4 red squares on the screen and are instructed to select the square that differs in size to the other 3. Subjects are paid progressively less per trial as they continue with the task [[14](#_bookmark154)]. They are also told that they can stop their participation in the task at any point, but that they still have to sit facing the screen for the remaining time (20 minutes minus the time they performed the task). The principle outcome measure is the number of trials until the subject stops the task (the break point). Two additional measures are calculated: running rate is calculated as the time taken to complete the block of trials, and the post reinforcement pause is calculated as the average time taken to initiate the next trial following a reward.

PANSS: The PANSS was developed and standardized for typological and dimensional assessment of schizophrenic phenomena [[15](#_bookmark155)[,16](#_bookmark156)]. The scale measures the following empirically derived factors: positive symptom factors (delusions, unusual thoughts, somatic concern, grandiosity, suspiciousness, and hallucinations), negative symptom factors (emotional withdrawal, blunted affect, poor rapport, disturbance of volition, preoccupation, and motor retardation), cognitive factors (difficulty in abstract thinking, stereotyped thinking, cognitive disorganization, lack of judgment and insight, poor attention, tension, mannerisms, and posturing), hostility (excitement, hostility, impulse control, and uncooperativeness), and emotional discomfort (depression, anxiety, and guilt). The total score of the PANSS is derived from the summation of each item. It takes

45 minutes to administer the scale.

BNSS: The BNSS is a13-item instrument designed for use in clinical trials and other studies that measures 5 domains of negative symptoms: blunted affect, alogia, asociality, anhedonia, and avolition [[17](#_bookmark157)]. Examination of the correlations between other instruments supports the discriminant validity (ie, low correlations with other instruments measuring positive symptoms) and convergent validity (ie, high correlations with other instruments measuring negative symptoms). It takes approximately 15 minutes to administer the BNSS.

CGI Scales: The CGI scales [[18](#_bookmark158)] consist of 2 subscales: the CGI-S and the CGI-I. It takes approximately 5 minutes to administer the CGI scales. The CGI-S assesses the clinician’s impression of the subject’s current mental illness state. The clinician should use his/her total clinical experience with this patient population and rate the current severity of the subject’s mental illness on a 7-point scale. The CGI-I assesses the subject’s improvement (or worsening). The clinician is required to assess the subject’s condition relative to Baseline (Day -1) on a 7-point scale. In all cases, the assessment should be made independent of whether the rater believes the improvement/worsening is drug-related or not.

Optional:

EEfRT: Based on animal paradigms, EEfRT is a computerized button-pressing task in which the subject chooses easy or hard tasks for variable amounts of reward. The hard task requires an individually calibrated number of button presses to be made within 30 seconds, with the nondominant pinkie finger. The easy task requires one-third the amount of the individually

calibrated hard number of presses to be made within 7 seconds with the dominant index finger. The rewards are grouped into low, medium, and high bins for analyses and the primary dependent variable is the percentage of hard choices within the high probability trials and percentage of hard choices at each reward level. EEfRT demonstrates fair to good test-retest reliability (ie, ICC value

0.79 [[5](#_bookmark147)]) and small effect size (ie, d value 0.17 [[5](#_bookmark147)]). Optional:

Empathic Accuracy task: This task involves subjects watching 9 video clips, each lasting ~2.0 to

2.5 minutes. Each clip shows the head and shoulders of 1 of 6 individuals (targets) while he/she discusses a positive or negative autobiographical event. For each clip, subjects are instructed to press 1 of 2 response keys on a computer keyboard to adjust how positive or negative they believe the individual is feeling throughout the duration of the clip based on a 9-point scale (ranging from 1=extremely negative to 9=extremely positive). Subjects can adjust their ratings as frequently as they felt necessary during the clip to adjust for changes in emotion. The primary dependent measure is the mean correlation across clips between the subjects’ ratings of the targets’ emotions and the targets’ ratings of their own emotions calculated in 2-s time epochs throughout the clip. Positive and negative valence clips are included. The empathic accuracy task demonstrates adequate test-retest reliability (ie, Pearson *r* was 0.72) and a medium to large effect size (ie, *d* value

.79 [[19](#_bookmark159)]).

- - - 1. *Exploratory Biomarker Research*

This trial employs a number of functional tests to assess their utility as potential PD markers for the effects of TAK-041 on motivation/reward and cognition, as well as social deficits in order to develop a tool box to be used in future proof of activity studies as well as next step proof of concept studies as exploratory PD markers.

- - - 1. *Optional Wearable Devices*

As an exploratory objective of this trial, the sponsor wishes to explore the utility of a number of endpoints derived from wearable digital devices for monitoring physical activities. Currently, the impact of a drug on activities of daily living (including physical activity level and sleep) is typically captured by questionnaires that rely on the subject’s recall. Wearable digital devices provide an opportunity to collect dense data with continuous monitoring, to obtain objective data on physical activity, and for these parameters to be monitored when the subject is not confined to the clinic. This component of the trial will evaluate these technologies and allow the sponsor to gain experience with the logistics of deploying them during a clinical trial. The data obtained will be exploratory in nature and will help in the design of future trials.

## Critical Procedures Based on Study Objectives: Timing of Procedures

For this trial, the MID Task-induced fMRI BOLD, BACS, and Grip Effort Task are the critical procedures.

- At any postdose time point, these procedures should occur as close as possible to the prescribed/scheduled time.
- All other procedures should be completed as close as possible, either before or after the prescribed/scheduled time.
- The order of priority can be changed during the study with joint agreement of the investigator and the sponsor.
- Any nonscheduled procedures required for urgent evaluation of safety concerns take precedence over all routine scheduled procedures.

## Study Design/Dosing/Procedures Modifications Permitted Within Protocol Parameters

This trial is a proof-of-activity assessment of TAK-041.

- The washout period between the doses in the 2 Treatment Periods may be lengthened (not to exceed 42 days total).
- The PK/PD sampling scheme currently outlined in the protocol may be modified during the trial based on newly available PK or PD data (eg, to obtain data closer to the time of peak plasma concentrations). If indicated, these collected samples may also be assayed in an exploratory manner for metabolites and/or additional PD markers. PK timepoint sampling windows are provided in a separate Laboratory Manual.
- Up to an additional 50 mL of blood may be drawn for PK and/or PD analyses. This may include repeat samples or modified PK/PD time points based on emerging data. The total blood volume withdrawn from any single subject will not exceed the maximum allowable volume during his/her participation in the entire trial.
- The timing of planned procedures for assessment of safety procedures (eg, vital signs, ECG, safety laboratory tests) currently outlined in the protocol may be modified during the study based on newly available safety, tolerability, PK, or PD data (eg, to obtain data closer to the time of peak plasma concentrations). These changes will not increase the number of trial procedures for a given subject during his/her participation in the entire trial.
- Additional laboratory safety tests may be added to blood samples previously drawn to obtain additional safety information (eg, adding creatinine kinase to serum chemistry panel that was already drawn).
- Exploratory PD assessments labeled in the protocol as ‘Optional’ may or may not be included during the conduct of the study based on whether the specific tasks are available and successfully operationalized at the research sites (see Section [6.1](#_bookmark31)). If included in the study procedures, these tasks will be conducted only in participants randomized to study treatment after the feasibility of the task (or tasks) have been demonstrated.

It is understood that the current trial may employ some or none of the alterations described above. Any alteration made to this protocol to meet the trial objectives must be detailed by the Sponsor in a letter to the Master File and forwarded to the investigator for retention. The letter may be

forwarded to the Institutional Review Board (IRB)/Independent Ethics Committee (IEC) at the discretion of the investigator.

## Study Beginning and End/Completion

## Definition of Beginning of the Study

The trial begins when the first subject signs the trial informed consent form.

## Definition of End of the Study

The overall trial ends when the last subject completes the last planned or follow-up visit/interaction associated with a planned visit, discontinues from the trial or is lost to follow-up (ie, the investigator is unable to contact the subject).

## Definition of Study Completion

The primary objective of this trial is to estimate the likelihood of attenuating motivation/reward deficits observed in schizophrenia by add-on TAK-041 administration to antipsychotics in subjects with stable schizophrenia. It is possible that study subjects may not receive all dose levels specified in the protocol, or that the study may be stopped before all planned subjects are enrolled if this objective is achieved early in this study. This is not considered an early termination of the trial, but rather an earlier than anticipated achievement of the trial objective or trial completion.

## Definition of Study Discontinuation

Study discontinuation due to non-safety reasons, such as:

- A finding (eg, PK, PD, biologic targets) from another preclinical or clinical trial using the same treatment results in the trial being stopped for a non-safety-related reason.
- Data from comparator, drug of the same class, or methodology used in this trial become available and result in the trial being stopped for a non-safety-related reason.
- The trial is stopped due to non-scientific and non-safety reasons, such as slow enrollment. Study discontinuation due to safety reasons:

Early trial termination due to unanticipated concerns of safety to the trial subjects arising from clinical or preclinical studies with the same treatment, drug of the same class, or methodology used in this trial.

## Criteria for Premature Termination or Suspension of the Study

The trial will be completed as planned unless one or more of the following criteria are satisfied that require temporary suspension or early termination of the trial.

- New information or other evaluation regarding the safety or efficacy of the trial medication that indicates a change in the known risk/benefit profile for the compound, such that the risk/benefit is no longer acceptable for subjects participating in the trial.
- Significant violation of Good Clinical Practice (GCP) that compromises the ability to achieve the primary trial objectives or compromises subject safety.
  - - 1. *Criteria for Premature Termination or Suspension of Study Sites*

A trial site may be terminated prematurely or suspended if the site (including the investigator) is found in significant violation of GCP, protocol, or contractual agreement, is unable to ensure adequate performance of the trial, or as otherwise permitted by the contractual agreement.

- - - 1. *Procedures for Premature Termination or Suspension of the Study or the Participation of Study Site*

In the event that the sponsor, an IRB/IEC, or regulatory authority elects to terminate or suspend the trial or the participation of an investigational site, a trial-specific procedure for early termination or suspension will be provided by the sponsor; the procedure will be followed by the investigational site during the course of termination or trial suspension.

## SELECTION AND DISCONTINUATION/WITHDRAWAL OF SUBJECTS

All entry criteria, including test results, need to be confirmed prior to randomization.

## Inclusion Criteria

Subject eligibility is determined according to the following criteria prior to entry into the trial:

1. The subject understands the trial procedures and agrees to participate by providing written informed consent.
2. The subject is willing and able to comply with all trial procedures and restrictions.
3. The subject is judged to be in good health by the investigator, based on clinical evaluations including laboratory safety tests, medical history, physical examination, 12-lead ECG, and vital sign measurements performed at the Screening Visit and prior to administration of the initial dose of study drug/invasive procedure.
4. The subject is male or female and 18 to 60 years of age, inclusive, at the Screening Visit.
5. A female subject of childbearing potential who is sexually active with a nonsterilized male partner must agree to use 2 highly effective methods of contraception from signing of informed consent throughout the duration of the trial and for 85 days after the last dose or she is postmenopausal or of non-childbearing potential.
6. A male subject who is nonsterilized and sexually active with a female partner of childbearing potential must agree to use adequate contraception from signing of informed consent throughout the duration of the trial and for 145 days after last dose. The female partner of a male subject should also be advised to use a highly effective method of contraception.
7. The subject is on a stable dose of an antipsychotic for at least 2 months as documented by medical history and assessed by site staff (other than those excluded in Table 7.a).
8. The subject meets schizophrenia criteria as defined by the DSM-5 by the Mini International Neuropsychiatric Interview (MINI).
9. The subject has PANSS total score ≤90 and PANSS negative symptom factor score (NSFS; Sum of PANSS N1, N2, N3, N4, N6, G7, and G16) ≥15 at screening and baseline (Day -1).
10. The subject has stable screening and baseline (Day -1) PANSS and NSFS total scores (<20% change).
11. The subject has had a structural brain MRI within the preceding year or during screening indicating no concerning structural brain abnormalities or other abnormalities that would interfere with interpretation of functional brain imaging results.

## Exclusion Criteria

The subject must be excluded from participating in the trial if the subject:

1. Has participated in another investigational trial within 4 weeks prior to the pretrial/screening visit. The 4-week window will be derived from the date of the last trial procedure and/or AE

related to the trial procedure in the previous trial to the pretrial/Screening Visit of the current trial.

1. Is an employee or immediate family member (eg, spouse, parent, child, sibling) of the Clinical Site or the Sponsor.
2. Has a history of cancer (malignancy).
3. Has a history of significant multiple and/or severe allergies (eg, food, drug, latex) or has had an anaphylactic reaction or significant intolerability to prescription or nonprescription drugs or food.
4. Has a positive alcohol and/or positive drug screen at Screening or Day -1.
5. Has a positive pregnancy test. (Female only).
6. Is a lactating/nursing female.
7. Is positive for hepatitis B surface antigen (HBsAg), hepatitis C virus (HCV) antibody, or human immunodeficiency virus (HIV) antibody/antigen (confirmatory testing is allowed; most sensitive test should take precedence).
8. The subject has abnormal Screening or baseline laboratory values (>ULN for the respective serum chemistries) of ALT, AST, TBILI, ALP, GGT, confirmed upon repeat testing, 5’nucleotidase (Screening only) and/or abnormal urine osmolality, confirmed upon repeat testing.
9. Had major surgery, or donated or lost 1 unit of blood (approximately 500 mL) within 4 weeks prior to the pretrial/Screening Visit.
10. Is unable to refrain from or anticipates the use of any medication (except those prescribed) as described in Section [7.3.](#_bookmark48)
11. Meets DSM-5 criteria for substance use disorder or history of alcohol abuse within 1 month prior to Screening Visit.
12. Has a history of claustrophobia or inability to tolerate mock scanner environment during habituation/screening session.
13. Fulfills any of the MRI contraindications on the site standard radiography screening document.
14. Has a history in the last year from the randomization visit or is currently receiving treatment with clozapine.
15. Has a current diagnosis of a significant psychiatric illness other than schizophrenia, per DSM-5 and is in an acute phase/episode.
16. Has a risk of suicide according to the investigator’s clinical judgment (eg, per C-SSRS positive answers on questions 4 or 5 for the last 6 months or has made a suicide attempt within

6 months prior to screening visit).

1. Is unsuitable for inclusion in the trial in the opinion of the investigator or sponsor.

## Excluded Medications, Supplements, and Dietary Products

Use of the agents in [Table 7.a](#_bookmark49) the (prescription or nonprescription) is prohibited at the time points specified.

## Table 7.a Excluded Medications, Supplements, and Dietary Products

**Disallowed (X) During the Study**

**(sections without [X] indicate no restrictions) (a)**

| **Drug Class** | **Chronic Use** | **Episodic use** | **Comments or Exceptions** |
| --- | --- | --- | --- |
| Any investigational drug | X | X | <60 days before Screening or |
|  |  |  | 5 half-lives – whichever is longer |
| Narcotic analgesics | X | X |  |
| Alcohol |  |  | Not restricted, except for 72 hours prior |
|  |  |  | to and during baseline assessments and |
|  |  |  | during PD testing on Day -1, Day 1, |
|  |  |  | and Day 14 in Periods 1 and 2. |
| Anorexiants (eg, phentermine, | X | X | Must be discontinued for ≥30 days |
| benzphetamine, phendimetrazine, |  |  | prior to Screening |
| methamphetamine, amphetamine, |  |  |  |
| stimulants, sibutramine, Belviq (lorcaserin), |  |  |  |
| Qsymia (phentermine/topiramate) |  |  |  |
| Antiarrhythmics of 1C class, quinidine | X | X |  |
| Antibiotics | X |  |  |
| Anticholinergics | X | X | Maximum dose of chronic |
|  |  |  | anticholinergic treatment is 2 mg/day of benztropine or equivalent. |
| Antithrombic agents and anticoagulants |  | X |  |
| (excluding warfarin, which is excluded) |  |  |  |
| Anticonvulsants | X | X | Exception: gabapentin and pregabalin |
|  |  |  | are permitted if they are prescribed at a |
|  |  |  | stable dose for ≥2 months prior to |
|  |  |  | Screening and throughout study |
|  |  |  | treatment. Subjects taking other |
|  |  |  | anticonvulsants should not be |
|  |  |  | considered for participation in the |
|  |  |  | study, as their discontinuation could |
|  |  |  | lead to symptom instability. |
| Antidepressants (excluding tricyclic |  | X | Tricyclic antidepressants, MAOIs, and |
| antidepressants, MAOIs, and RIMAs) |  |  | RIMAs are excluded, and subjects |
|  |  |  | taking them should not be considered |
|  |  |  | for participation in the study, as their |
|  |  |  | discontinuation could lead to symptom |
|  |  |  | instability. |
| Footnotes are on last table page. |  |  |  |

**Disallowed (X) During the Study**

**(sections without [X] indicate no restrictions) (a)**

| **Drug Class** | **Chronic Use** | **Episodic use** | **Comments or Exceptions** |
| --- | --- | --- | --- |
| Antihistamines | X | X | Except loratadine, desloratadine, |
|  |  |  | cetirizine, levocetirizine, mizolastine, |
|  |  |  | and fexofenadine. |
| Antihypertensives |  |  | Clonidine NOT allowed. |
| Antipsoriatic agents | X | X | Topical agents are allowed. |
| Antipsychotics |  | X | Phenothiazines (chlorpromazine, |
|  |  |  | perphenazine, prochlorperazine, |
|  |  |  | thioridazine), haloperidol, and |
|  |  |  | clozapine are excluded and must be |
|  |  |  | discontinued 60 days prior to |
|  |  |  | screening; all other treatments must |
|  |  |  | adhere to requirements outlined in the |
|  |  |  | study entry criteria. |
|  |  |  | As an exception, occasional use of an |
|  |  |  | additional dose of the background |
|  |  |  | antipsychotic may be permitted with |
|  |  |  | sponsor or designee approval. |
| Fruit juice |  |  | Not restricted, except that dosing will |
|  |  |  | occur without consumption of fruit |
|  |  |  | juice. Fruit juice is restricted until 4 |
|  |  |  | hours after dosing. |
| Herbal remedies, which are psychoactive | X | X | Must be discontinued ≥7 days prior to |
| (eg, St John’s Wort, kava, valerian, ginkgo biloba,) |  |  | Screening. |
| Melatonin | X | X | Must be discontinued ≥7 days prior to |
|  |  |  | Screening. |
| Sedative hypnotics |  |  | Barbiturates are excluded. Chronic |
|  |  |  | treatment with BZs is allowed up to 3 |
|  |  |  | mg/day lorazepam or equivalent (BZ |
|  |  |  | equivalence standards will be provided |
|  |  |  | in a site reference document). |
| Insulin | X | X |  |
| Mood stabilizers | X | X | Lithium, valproic acid, and lamotrigine |
| Footnotes are on last table page. |  |  |  |

**Disallowed (X) During the Study**

**(sections without [X] indicate no restrictions) (a)**

| **Drug Class** | **Chronic Use** | **Episodic use** | **Comments or Exceptions** |
| --- | --- | --- | --- |
| Medications that may interfere with |  |  | Such as cold medicines containing |
| cognitive function |  |  | antihistamine or dextromethorphan as a |
|  |  |  | cough suppressant are not restricted |
|  |  |  | except for 7 days prior to and during |
|  |  |  | baseline assessments and during PD testing on Day -1 and Day 1; and 7 days |
|  |  |  | prior to and during the Day 14 |
|  |  |  | assessments in Periods 1 and 2. |
| Medicines in the statin class | X | X | Atorvastatin, fluvastatin, lovastatin, |
|  |  |  | pitavastatin, pravastatin, rosuvastatin, |
|  |  |  | and simvastatin. |
| Psychotropic agents known to affect | X | X | Such as long-acting sleep aids, |
| cognition |  |  | amphetamines, barbiturates, lithium, |
|  |  |  | methylphenidate, anticholinergics, |
|  |  |  | antidepressants (eg, tricyclic |
|  |  |  | antidepressants and MAOIs). Note: the |
|  |  |  | definitive guidelines for each |
|  |  |  | medication/class entry in this row is the |
|  |  |  | primary table row for each |
|  |  |  | medication/class. |
| Steroids Systemic oral or | X | X | As an exception, treatment with local |
| injectable |  |  | steroid injections for orthopedic |
|  |  |  | conditions may be permitted with |
|  |  |  | sponsor or designee approval. |
| Topical |  |  |  |
| Inhalant |  |  |  |
| Stimulants | X | X | Must be discontinued for ≥30 days |
|  |  |  | prior to Screening. This category does |
|  |  |  | not include substances containing |
|  |  |  | caffeine or nicotine. |
| Tobacco- and nicotine-containing products |  |  | Not restricted, except for 2 hours prior |
|  |  |  | to and during baseline assessments and |
|  |  |  | during PD testing on Day -1, Day 1, and Day 14 in Periods 1 and 2 (unless |
|  |  |  | the subject exhibits nicotine |
|  |  |  | withdrawal symptoms that pose a risk |
|  |  |  | to the study PD assessments in the |
|  |  |  | judgment of the investigator). |
| UGT enzyme inhibitors (probenecid and |  |  | Not within 14 days of dosing. |
| valproic acid) |  |  |  |
| Footnotes are on last table page. |  |  |  |

## Table 7.a Excluded Medications, Supplements, and Dietary Products (continued)

**Disallowed (X) During the Study**

**(sections without [X] indicate no restrictions) (a)**

**Drug Class Chronic Use Episodic use Comments or Exceptions**

Xanthine and/or caffeine Not restricted, except for 4 hours prior to and during baseline assessments and during PD testing on Day -1, Day 1, and Day 14 in Periods 1 and 2.

BZ=benzodiazepine, MAOI=monoamine oxidase inhibitor, RIMA=reversible inhibitor of monoamine oxidase type A, UGT=uridine 5'-diphosphate-glucuronosyltransferase.

(a) If medications are required to treat an AE, this will be done based on medical need. However, continuation in the trial may be allowed for certain medications after discussion and agreement between the sponsor and principal investigator.

Occasional use of acetaminophen/paracetamol (≤1 g/day) or other medication as approved by Takeda on a case-by-case basis is allowed.

Concomitant medications to treat concurrent diseases or co-morbidities should be maintained at a stable dose and regimen from the Screening Visit throughout the trial; however, medication adjustments may be made by the investigator based on the subject’s condition and standard of care.

Subjects must be instructed not to take any medications, including OTC products without first consulting with the investigator. However, if a medication is taken, it is the responsibility of the investigator to ensure that the details regarding the medication are recorded in full in the electronic case report form (eCRF).

## Diet, Fluid, Activity

## Diet and Fluid

There are no restrictions on diet or intake of fluids, except as specifically noted.

## Activity

There are no restrictions on activity, except to avoid strenuous activity 24 hours before a clinic visit.

## Criteria for Discontinuation or Withdrawal of a Subject

The primary reason for discontinuation or withdrawal of the subject from the trial should be recorded in the eCRF using the following categories.

1. Pretreatment event or AE. The subject has experienced a pretreatment event or AE that requires early termination because continued participation imposes an unacceptable risk to the subject’s health or the subject is unwilling to continue because of the pretreatment event or AE.
   - Liver Function Test Abnormalities

Based on local country criteria, subjects in a clinical trial who experience ALT >3× ULN and TBILI >2×ULN and satisfy the following 2 criteria: (1) the liver injury is hepatocellular in nature and there is not a prominent cholestatic component; (2) there is no more likely alternative cause than drug induced liver injury, such as acute viral hepatitis A or B, or other acute liver disease.

If ALT or AST is >3×ULN, laboratory tests should be repeated within a maximum of 7 days, and preferably within 48 to 72 hours. If the ALT or AST remains elevated >3×ULN on these 2 consecutive occasions, and this observation cannot be explained by concomitant disease or another alternative etiology, the abnormality should be recorded as an AE. The investigator must contact the Medical Monitor for consideration of immediate discontinuation of trial medication, discussion of the relevant subject details and possible alternative etiologies.

In addition, trial medication should be discontinued immediately with appropriate clinical follow-up, including repeat laboratory tests, until a subject’s laboratory profile has returned to normal, if the following circumstances occur at any time during trial medication treatment:

- ALT or AST >8×ULN, or
- ALT or AST >5×ULN and persists for more than 2 weeks, or
- ALT or AST >3×ULN in conjunction with elevated total bilirubin >2 × ULN or international normalized ratio >1.5.
- ALT or AST >3×ULN with appearance of fatigue, nausea, vomiting, right upper quadrant pain or tenderness, fever, rash and/or eosinophilia (>5%).
- ALP elevations >1.5×ULN in conjunction with elevated total bilirubin >1.5×ULN or elevated 5'-nucleotidase >2×ULN or elevated GGT >5×ULN or elevated AST/ALT

>3×ULN or elevated ALP >2×ULN, persisting for longer than 3 days.

- ALP >3×ULN.
  - Psychiatric Emergencies

Subjects with the following conditions will be discontinued from the trial with appropriate follow-up (which may include immediate contact with the subject’s mental health practitioner; and/or possible referral to the emergency room; and/or admission to an

in-patient unit):

- - - Subjects who experience acute psychotic episode during the study, requiring hospitalization or a change in the current treatment regimen.
    - Subjects who experience suicidal ideation with intent to act or with a specific plan (C-SSRS level 4 and 5) or any suicidal behavior.

1. Protocol deviation. The discovery post-randomization that the subject failed to meet protocol entry criteria or did not adhere to protocol requirements, and continued participation poses an unacceptable risk to the subject’s health.
2. Lost to follow-up. The subject did not return to the clinic and attempts to contact the subject were unsuccessful. Attempts to contact the subject must be documented.
3. Withdrawal by subject. The subject wishes to withdraw from the trial.
4. Withdrawal by parent/guardian. The subject’s legally acceptable representative wishes to withdraw the subject from the trial.

Note: All attempts should be made to determine the underlying reason for the withdrawal and, where possible, the primary underlying reason should be recorded (ie, withdrawal due to an AE should not be recorded in the “voluntary withdrawal” category).

1. Study terminated by sponsor. The sponsor, IRB, IEC, or regulatory agency terminates the trial.
2. Pregnancy. The subject is found to be pregnant.

Note: If the subject is found to be pregnant, the subject must be withdrawn immediately. The procedure is described in Section [7.6](#_bookmark54).

1. Other.

Note: The specific reasons should be recorded in the “specify” field of the eCRF.

Note: Skin reactions (cutaneous hypersensitivity) to a wearable digital device will lead to removal of that device for the remainder of the trial. The subject would not be required to discontinue the trial as a whole.

## Procedures for Discontinuation or Withdrawal of a Subject

The investigator may terminate a subject’s trial participation at any time during the study when the subject meets the trial termination criteria described in Section [7.5.](#_bookmark53) In addition, a subject may discontinue his or her participation without giving a reason at any time during the study. Should a subject’s participation be discontinued, the primary criterion for termination must be recorded. In addition, efforts should be made to perform all procedures scheduled for the Early Termination Visit.

## Subject Replacement

Discontinued or withdrawn subjects may be replaced at the discretion of the study team. Up to 32 subjects will be randomized to ensure 24 subjects complete postdose PD assessments on Day 1 of both Treatment Periods.

## CLINICAL STUDY MATERIAL MANAGEMENT

## Clinical Study Drug

The TAK-041 oral suspension will be used in this study. The TAK-041 crystalline drug substance (milled) will be labeled in an open-label fashion and compounded into oral suspensions that will be labeled in a blinded fashion for third party dispensing. An unblinded pharmacist will manage and prepare doses as needed throughout the study. The oral suspensions will contain 5 to 160 mg of TAK-041 per dose.

## Investigational Drug

The oral suspension containing crystalline TAK-041 (milled) will be prepared at a pharmacy laboratory by weighing an appropriate amount of crystalline TAK-041 (milled) into a dosing bottle and mixing with 70 mL of 0.5% Tween 80 in 0.5% methylcellulose vehicle (Tween/MC vehicle). The bottle is wrapped with aluminum foil to cover the appearance of the suspension for blinding purposes. The composition of the Tween/MC vehicle is in [Table 8.a](#_bookmark59). The composition of crystalline TAK-041 oral suspension is listed in [Table 8.b.](#_bookmark60)

## Table 8.a Composition of Tween/MC Vehicle

**Component Composition per 100 mL of Water**

Methylcellulose, USP 500 mg

Tween 80, NF 500 mg

Sterile water for irrigation, USP 100 mL

NF=National Formulary, USP=United States Pharmacopeia.

## Table 8.b Composition of Crystalline TAK-041 Oral Suspension

**Component Quantity per Dose**

Crystalline TAK-041 (milled) (a) mg

Tween/MC vehicle 70 mL

(a) Amount shall be in the range of 5 mg to 160 mg, which corresponds to a dose of 5 to 160 mg TAK-041, and a concentration range of 0.07 to 2.29 mg/mL TAK-041.

A placebo will be prepared by adding 70 mL of 0.5% Tween/MC vehicle into a unit dosing bottle and wrapped with aluminum foil to cover the appearance of the suspension for blinding purposes.

## Storage

Investigational drug must be kept in an appropriate, limited-access, secure place until it is used or returned to the sponsor or designee for destruction. Investigational drug must be stored under the conditions specified on the label, and remain in the original container until dispensed.

TAK-041 crystalline drug substance (milled) is stored at controlled room temperature (20°C-25°C with excursions allowed from 15°C-30°C).

## Dose and Regimen

The planned initial dose of TAK-041 is 40 mg or matching placebo. The dose level is subject to change and will be determined based on the available safety, tolerability, and PK data from an equivalent dose cohort in healthy volunteers in Study TAK-041-1001. The doses will be administered to the subjects by the investigator or the investigator’s designee. Subjects will receive the doses by drinking the entire suspension from the dosing bottle. The dosing bottle will then be rinsed with 35 mL of water and the rinse will be administered in the same manner as the suspension. The rinse and administration will be repeated one more time.

## Overdose

An overdose is defined as a known deliberate or accidental administration of investigational drug, to or by a study subject, at a dose above that which is assigned to that individual subject according to the study protocol.

All cases of overdose (with or without associated AEs) will be documented on an Overdose page of the eCRF, in order to capture this important safety information consistently in the database.

Cases of overdose without manifested signs or symptoms are not considered AEs. AEs associated with an overdose will be documented on AE CRF(s) according to Section [10.0](#_bookmark95), Pretreatment Events and Adverse Events.

SAEs associated with overdose should be reported according to the procedure outlined in Section [10.2.8](#_bookmark107), Collection and Reporting of SAEs.

In the event of drug overdose, the subject should be treated symptomatically.

## Clinical Study Drug Labeling

Clinical study drug packaging will be affixed with a clinical label in accordance with regulatory requirements.

## Clinical Study Drug Inventory

Inventory (receipt and dispensing) of study drug must be recorded by an authorized unblinded person at the trial site.

## Clinical Study Drug Blinding

This is a double-blind trial. The study drug blind is maintained through a randomization schedule held by the randomization personnel. Investigational drug doses will be manufactured in a manner so as to minimize the possibility of identification of different doses through appearance. Further details will be provided in the Pharmacy Manual.

## Randomization Code Creation and Storage

Takeda Biostatistics Department or designee will generate the randomization schedule and a copy will be provided to the randomization personnel prior to the start of this study. The randomization

will be stratified by site. All randomization information will be stored in a secured area, accessible only by authorized personnel.

## Clinical Study Blind Maintenance/Unblinding Procedure

The investigational drug blind is maintained through a randomization schedule held by the randomization personnel.

The investigational drug blind shall not be broken by the investigator unless information concerning the investigational drug is necessary for the medical treatment of the subject. All trial assessments and causality should be performed, if possible, prior to unblinding. In the event of a medical emergency, if possible, the medical monitor or designee should be contacted before the investigational drug blind is broken to discuss the need for unblinding. The sponsor must be notified as soon as possible if the investigational drug blind is broken.

## Accountability and Destruction of Sponsor-Supplied Drugs

The investigator is responsible for keeping accurate records of the clinical study drug received from the sponsor or designee, the amount dispensed to and returned by the subjects, and the amount remaining at the conclusion of the study. For all study sites, the local country sponsor personnel or designee will provide appropriate documentation that must be completed for clinical study drug accountability, return, and destruction.

## STUDY PROCEDURES

The following sections describe the trial procedures and data to be collected as indicated in the Schedule of Study Procedures (Section [3.0](#_bookmark15)). For each procedure, subjects are to be assessed by the same investigator or site personnel whenever possible. Please note that it may become necessary to perform the following procedures at unscheduled time periods, per the discretion of the investigator.

## Administrative Procedures

## Informed Consent Procedure

The requirements of informed consent are described in Section [13.2](#_bookmark126). Informed consent must be obtained prior to the subject entering into the trial and before any protocol-directed procedures are performed, including requesting that a subject fast for laboratory evaluations. Pharmacogenomics informed consent is a component of the overall trial informed consent. A separate optional informed consent is required for the digital device component of the trial. Subject participation in the wearable device component of the trial is optional.

When subjects have performed screening assessments prior to trial, the data from screening using a generic screening consent form can be used in this trial for those who were subsequently enrolled, as long as the procedure was performed within the protocol screening to enrollment window.

- - - 1. *Assignment of Screening and Randomization Numbers*

All consented subjects will be given a unique screening number that will be used to identify the subject for all procedures that occur prior to randomization or allocation. Each subject will be assigned only 1 screening number. Screening numbers must not be re-used for different subjects. Any subject who is screened multiple times will be assigned a new screening number for each screening event.

All eligible subjects will be randomized and will receive a randomization number. The randomization number identifies the subject for all procedures occurring after randomization. A single subject cannot be assigned more than 1 randomization number.

- - - 1. *Clinical Study Drug Assignment*

On Day 1 of Period 1, eligible subjects will be randomized 1:1 to 1 of 2 sequence groups. See [Table 6.a](#_bookmark32) for a description of the drug assignments in Periods 1 and 2 for each sequence group. Each sequence group will consist of 12-16 subjects. Each participant will be dispensed blinded trial treatment, labeled with his/her unique randomization number, throughout the trial.

## Inclusion and Exclusion Criteria

Each subject is assessed through randomization, according to the eligibility criteria provided in Section [7.0](#_bookmark45).

## Medical History/Demographics

Qualified site personnel are to collect subject significant medical history (past and ongoing) per the site’s standard of care and appropriate clinical judgment as well as subject demographics.

## Prior and Concomitant Medication Review

Medications are defined as prescription and over-the-counter drugs, vitamin supplements, nutraceuticals, and oral herbal preparations. Qualified site personnel are to review subject medication use.

## Clinical Procedures and Assessments

## Full Physical Exam

Qualified site personnel will conduct full physical examinations.

## Height and Weight

Body weight and height will be obtained with the subject’s shoes off, and jacket or coat removed.

## Body Mass Index

BMI equals a person’s weight in kilograms divided by height in meters squared (BMI=kg/m^2^). BMI will be rounded to the nearest whole number according to the standard convention of

0.1 to 0.4 rounds down and 0.5 to 0.9 rounds up.

## Vitals (Body Temperature, Heart Rate, Blood Pressure)

Body temperature will be measured with an oral (temperature taken at floor of the mouth) or tympanic thermometer. The same method (eg, oral or tympanic) must be used for all subsequent measurements for each individual subject and should be the same for all subjects.

Subjects should rest in a semirecumbent position for at least 5 minutes prior to having vital sign measurements obtained. Vital signs will include heart rate, systolic blood pressure, and diastolic blood pressure. The same method (eg, same size cuff, manual or automated) must be used for all measurements for each individual subject and should be the same for all subjects.

## 12-Lead ECG

Special care must be taken for proper lead placement by qualified personnel. Skin should be clean and dry prior to lead placement. Subjects may need to be shaved to ensure proper lead placement. Female subjects may need to remove their bra.

Subjects should be resting in a semirecumbent position for at least 5 minutes prior to each ECG measurement.

QT intervals with Fridericia correction method (QTcF) will be used to calculate QT intervals in this trial.

Prior to each Treatment Period, a predose ECG will be obtained within approximately 1 hour prior to dosing TAK-041. This measurement will be used as the Baseline. The principal investigator should arrange to have a trial cardiologist available as needed to review ECG tracings with abnormalities.

During each Treatment Period, if a subject demonstrates an increase in QTcF interval ≥40 msec compared with a predose baseline measurement, the ECG will be repeated within 5 minutes. The average value of the QTcF interval from the 2 ECGs will represent the value at that time point. If the average QTcF interval increase from Baseline for any postdose time point is ≥40 msec, the subject will continue to be monitored by repeat 12-lead ECGs every 60 minutes for at least 4 hours or until the QTcF is within 40 msec of the baseline value. If prolongation of the QTcF interval

≥40 msec persists, a consultation with a trial cardiologist may be appropriate and the Sponsor should be notified.

If the QTcF interval is ≥500 msec, the Sponsor should be notified and the ECGs should be reviewed by a cardiologist. The subject should be telemetry-monitored (until the QTcF is

<500 msec) or should be considered for transfer to a location where closer monitoring is available.

If the subject has unstable hemodynamics, or has any clinically significant dysrhythmias noted on telemetry, the subject should be immediately transferred to an acute care setting for definitive therapy.

If repeat ECGs are required, the clinical site will decide whether to leave the electrodes in place or mark the position of the electrodes for subsequent ECGs. To mark the position of the electrodes, 12-lead electrode sites will be marked on the skin of each subject with an ECG skin marker pen to ensure reproducible electrode placement.

The following ECG parameters will be recorded: heart rate, PR-interval, QRS-duration, QT interval, QTcF interval, and the interpretation of the ECG profile by the principal investigator.

## Study Drug Administration

The dose of study drug (TAK-041 or placebo) will be administered to the subjects by the investigator or the investigator’s designee. Subjects will receive the doses by drinking the entire suspension from the dosing bottle. The dosing bottle will then be rinsed with 35 mL of water and the rinse will be administered in the same manner as the suspension. The rinse and administration will be repeated one more time.

## Cognitive Assessments

The first block of PD tests will include the Grip Effort Task, Perceptual Effort Test, Progressive Ratio Test, and BACS. After a break, the imaging tests will begin, including ASL MRI, rs-fMRI BOLD, and t-fMRI BOLD with MID Reward Task and a Shifting Task. The third block of tests will include the EEfRT, the Empathic Accuracy Task and begin after a further break.

Subjects will also receive the PANSS, BNSS, and CGI assessments.

A trial manual describing the procedures to be used with these assessments will be supplied to the sites. The assessments will be performed at the times and flexibility stipulated in the Schedule of Study Procedures, [Table 3.a.](#_bookmark16) PD assessments and testing take priority over all other procedures.

## AE Monitoring

AE monitoring begins following signing of informed consent. Changes in subject health status from baseline assessment to study drug administration should be captured. A complete description of AE collections and procedures is provided in Section [10.0.](#_bookmark95)

## Actigraphy Monitoring

An actigraphy device will capture data on the subject’s activity level, including intensity of movement, rest, and sleep for exploratory purposes. The actigraphy device will be deployed on Day 1 after all PD assessments have been completed and the Hour 6 PK sample collection and will be worn continuously for approximately 14 days until subjects return to the site for Day 14 testing. The data derived from the wearable digital device will not be analyzed until the end of the trial and will not be recorded in the eCRF. The data are not intended to be analyzed for real-time monitoring but may be used for future retrospective analyses.

## Laboratory Procedures and Assessments

Laboratory samples will be collected in accordance with acceptable laboratory procedures. Samples will be taken on the days stipulated in the Schedule of Study Procedures (Section [3.0](#_bookmark15)).

- - - 1. *Clinical Laboratory Tests Hematology*

Hematology will consist of the following tests:

Erythrocytes (red blood cells [RBCs]) Hemoglobin

Hematocrit Platelets

Leukocytes (white blood cells [WBCs]) with absolute differential

*Urinalysis*

Urinalysis will consist of the following tests:

Protein Glucose

Blood Nitrite

Urine microscopy will be performed if urinalysis is abnormal. Microscopy consists of RBC/high-power field, WBC/high-power field, and casts.

*Chemistry*

Chemistry evaluations will consist of the following standard chemistry panel:

Albumin Alkaline phosphatase

ALT AST

Blood urea nitrogen Calcium

Carbon dioxide Chloride

Creatinine Glucose

Gamma-glutamyl transferase Sodium

Potassium Bilirubin (total), if above ULN, will be fractionated

Protein (total) 5'-nucleotidase

- - - 1. *DILI follow-up testing:*

5’nucleotidase, GGT, ALT, AST, INR, and any other laboratory tests deemed necessary after discussion with the medical monitor

- - - 1. *Diagnostic Screening Chemistry (Serum)*

Serum diagnostic evaluations will include the following tests:

β–human chorionic gonadotropin (females only) Follicle-stimulating hormone (FSH) (females only)

Hepatitis panel (including HBsAg and anti-HCV), HIV, and TSH

*Alcohol Screen*

Subjects will undergo an alcohol breath test. A urine alcohol test may be performed at the discretion of the investigator.

*Drug Screen (Urine)*

A urine drug screen will include the following tests:

Amphetamines 3,4-methylenedioxy-methamphetamine (MDMA)

Barbiturates Methadone/metabolite

Benzodiazepines Opiates

Buprenorphine/metabolite Oxycodone/oxymorphone

Cannabinoids Phencyclidine

Cocaine/metabolites

## C-SSRS

Suicidal ideation will be assessed using the C-SSRS at the times stipulated in the Schedule of Study Procedures (Section [3.0](#_bookmark15)). Two versions of the C-SSRS will be used in this trial: the Screening C-SSRS Lifetime Version 14Jan2009 and the Since-Last-Visit C-SSRS Version 14Jan2009.

## Biomarker, PK, PD, and PGx Samples

Samples for PK analysis will be collected as specified in the Schedule of Study Procedures (Section [3.0](#_bookmark15)). If a PK collection time point overlaps with PD assessment or test, the PD assessment should be prioritized. Please refer to the Laboratory Manual for information on the collection including sample windows, processing, and shipment of samples to the Central Laboratory. Some analyses will be exploratory in nature and may be used to identify or validate biomarkers.

The decision as to which plasma collected will be assayed for evaluation of PK will be determined by the Sponsor (eg, samples at lower doses may not be assayed if samples at higher doses reveal undetectable drug concentrations). If indicated, these samples may also be assayed and/or pooled for assay in an exploratory manner for metabolites and/or additional biomarkers.

Primary specimen collection parameters are provided in [Table 9.a.](#_bookmark90)

## Table 9.a Primary Specimen Collections

| **Specimen Name** | **Primary Specimen** | **Primary Specimen Derivative** | **Description of Intended Use** | **Sample Collection** |
| --- | --- | --- | --- | --- |
| Plasma sample for PK | Plasma | N/A | Plasma sample for PK analysis | Mandatory |
| Blood samples for DNA | Blood | DNA | PGx measurements | Optional |
| Blood samples for RNA | Blood | RNA | PGx measurements | Optional |

## PK Measurements

Samples for PK analysis will be collected as specified in the Schedule of Study Procedures (Section [3.0](#_bookmark15)). Please refer to the Laboratory Manual for information on the collection, including sample windows, processing, and shipment of samples to the Central Laboratory.

It is anticipated that the total blood volume drawn for an individual subject during this trial will be approximately 130.5 mL.

The PK parameters of TAK-041 will be derived using noncompartmental analysis methods and will be determined from the concentration-time data for all evaluable subjects. Actual sampling times, rather than scheduled sampling times, will be used in all PK computations involving sampling times. A more detailed description will be given in the clinical pharmacology analysis plan (CPAP).

The following plasma PK parameters for TAK-041 will be calculated:

- Area under the plasma concentration-time curve from time 0 to time t (AUC_t_)_._
- Area under the plasma concentration-time curve from time 0 to infinity (AUC_∞_)_._
- Cmax.
- Time of first occurrence of C_max_ (t_max_)_._
- t1/2z.

Additional PK parameters may be calculated if deemed necessary for the interpretation of the data. All efforts will be made to obtain the PK samples at the exact nominal time relative to dosing.

However, samples obtained within 10% of the nominal time from dosing will not be captured as a protocol deviation, as long as the exact time of the sample collection is noted on the eCRF.

## Biomarker Measurements

This trial employs number of functional tests to assess their utility as potential PD markers for the effects of TAK-041 on motivation/reward, cognition, as well as social deficits in order to develop a tool box to be used in future proof of activity studies as well as next step proof of concepts studies as exploratory PD markers. No plasma biomarkers will be tested.

## PGx Measurements

- - - 1. *Blood Samples for DNA and RNA PGx Measurements*

While sampling of whole blood for PGx analysis requires informed consent, participation in this assessment is optional.

PGx is the study of variations of DNA and RNA characteristics as related to drug response. There is increasing evidence that an individual’s genetic background may impact the PK (absorption, distribution, metabolism, and excretion), pharmacodynamics (pharmacologic effects), and/or the clinical outcome (efficacy and/or safety).

PGx research in this study may be conducted to understand how individual genetic variation in subjects impacts their study drug treatment response. This information may be also be used, for example, to develop a better understanding of the safety and efficacy of TAK-041 and other study drugs, to increase understanding of the disease/condition being studied and other related conditions, gain a better understanding of the drug pharmacology and for generating information needed for research, development, and regulatory approval of tests to predict response to

TAK-041.

Whole blood samples for DNA and RNA isolation will be collected from each consented subject in the trial. If necessary and feasible, a second aliquot of blood may be taken at a later time point if isolation of DNA from the first sample was not successful or possible.

Since PGx is an evolving science, many genes and their functions are not yet fully understood. Future data may suggest a role of these genes in drug response, which may lead to additional hypothesis-generating exploratory research and diagnostic development on stored samples.

Detailed instructions for the handling and shipping of samples are provided in the Laboratory Manual.

- - - 1. *Biological Sample Retention and Destruction*

In this trial, specimens for genome/gene analysis will be collected as described in the Laboratory Manual. The genetic material will be initially stored at the vendor or a comparable laboratory under contract to Takeda, with validated procedures in place, and then preserved and retained at the vendor or a comparable laboratory with validated procedures in place, for up to but not longer than 15 years from the end of the trial when the trial report is signed, or if less, the maximum period permitted under applicable law or until consent is withdrawn.

The sponsor and vendors working with the sponsor will have access to the samples collected and any test results. All samples collected during the study will be stored securely with limited access and the sponsor will require anyone who works with the samples to agree to hold the research information and any results in confidence.

The sample will be labeled with a unique sample identifier as in the main trial but using a code that is different from the code attached to the health information and other clinical test results collected in the trial. The sample and data are linked to personal health information with code numbers; the samples are stripped of all personal identifying information but a key linking the samples to clinical analysis data exists. This link means that the subject may be identified but only indirectly. The sample identifier will be kept secure by or on behalf of the sponsor.

Subjects who consent and provide a sample for DNA and RNA analysis can withdraw their consent at any time and request disposal of a stored sample. Any remaining sample that can be identified as coming from the subject will be destroyed. The trial doctor and sponsor may continue to use and distribute any information and test results gathered before the request to withdraw.

## Confinement

There is no confinement in this trial.

## ADVERSE EVENTS

## Definitions and Elements of AEs

An AE is defined as any untoward medical occurrence in a clinical investigation subject who has signed informed consent to participate in a trial; it does not necessarily have to have a causal relationship with the treatment.

An AE can therefore be any unfavorable and unintended sign (eg, a clinically significant abnormal laboratory finding), symptom, or disease temporally associated with the use of a drug, whether or not it is considered related to the drug.

An untoward finding generally may:

- Indicate a new diagnosis or unexpected worsening of a preexisting condition. (Intermittent events for pre-existing conditions underlying disease should not be considered AEs.)
- Necessitate therapeutic intervention.
- Require an invasive diagnostic procedure.
- Require discontinuation or a change in dose of trial medication or a concomitant medication.
- Be considered unfavorable by the investigator for any reason. Diagnoses versus signs and symptoms:
- Each event should be recorded to represent a single diagnosis. Accompanying signs (including abnormal laboratory values or ECG findings) or symptoms should NOT be recorded as additional AEs. If a diagnosis is unknown, sign(s) or symptom(s) should be recorded appropriately as an AE(s).

Laboratory values and ECG findings:

- Changes in laboratory values or ECG parameters may be considered to be AEs if they are judged to be clinically significant (ie, if some action or intervention is required or if the investigator judges the change to be beyond the range of normal physiologic fluctuation). A laboratory re-test and/or continued monitoring of an abnormal value are not considered an intervention. In addition, repeated or additional noninvasive testing for verification, evaluation or monitoring of an abnormality is not considered an intervention.
- If abnormal laboratory values or ECG findings are the result of pathology for which there is an overall diagnosis (eg, increased creatinine in renal failure), the diagnosis only should be reported appropriately as an AE.

Pre-existing conditions:

- A pre-existing condition (present before signing of the informed consent) is considered a concurrent medical history condition and should NOT be recorded as an AE. A baseline evaluation (laboratory test, ECG, X-ray, etc) present before signing of the informed consent should NOT be recorded as an AE. However, if the subject experiences a worsening or

complication of such a concurrent medical history condition, the worsening or complication should be recorded appropriately as an AE (worsening or complication occurs after informed consent is signed). Investigators should ensure that the event term recorded captures the change in the condition (eg, “worsening of…”).

- If a subject has a pre-existing episodic condition (eg, asthma, epilepsy), any occurrence of an episode should only be captured as an AE if the episodes become more frequent, serious, or severe in nature, that is, investigators should ensure that the AE term recorded captures the change from Baseline in the condition (eg “worsening of…”).
- If a subject has a degenerative concurrent condition (eg, cataracts, rheumatoid arthritis), worsening of the condition should only be captured as an AE if occurring to a greater extent to that which would be expected. Again, investigators should ensure that the AE term recorded captures the change in the condition (eg, “worsening of…”).

Worsening of AEs:

- If the subject experiences a worsening or complication of an AE after the first administration of trial medication or after any change in trial medication, the worsening or complication should be recorded as a new AE. Investigators should ensure that the AE term recorded captures the change in the condition (eg, “worsening of…”).

Changes in severity of AEs:

- If the subject experiences a change in the severity of an AE that is not associated with a change in trial medication, the event should be captured once with the maximum severity recorded.

Preplanned surgeries or procedures:

- Preplanned procedures (surgeries or therapies) that were scheduled prior to signing of informed consent are not considered AEs. However, if a preplanned procedure is performed early (eg, as an emergency) due to a worsening of the pre-existing condition, the worsening of the condition should be captured appropriately as an AE. Complications resulting from any planned surgery should be reported as AEs.

Elective surgeries or procedures:

- Elective procedures performed where there is no change in the subject’s medical condition should not be recorded as AEs but should be documented in the subject’s source documents. Complications resulting from an elective surgery should be reported as AEs.

Overdose:

- An overdose is defined as a known deliberate or accidental administration of investigational drug, to or by a trial subject, at a dose above that which is assigned to that individual subject according to the trial protocol. It is up to the investigator or the reporting physician to decide whether a dose is to be considered an overdose, in consultation with the Sponsor.
- All cases of overdose (with or without associated AEs) will be documented on an Overdose page of the (e)CRF, in order to capture this important safety information consistently in the

database. AEs associated with an overdose will be documented on AE CRF(s) according to Section [10.0](#_bookmark95).

- SAEs of overdose should be reported according to the procedure outlined in Section [10.2.8](#_bookmark107).
- In the event of drug overdose, the subject should be treated symptomatically.

## SAEs

An SAE is defined as any untoward medical occurrence that at any dose:

1. Results in DEATH.
2. Is LIFE THREATENING.
   - The term “life threatening” refers to an event in which the subject was at risk of death at the time of the event; it does not refer to an event that hypothetically might have caused death if it were more severe.
3. Requires inpatient HOSPITALIZATION or prolongation of existing hospitalization.
4. Results in persistent or significant DISABILITY/INCAPACITY.
5. Is a CONGENITAL ANOMALY/BIRTH DEFECT.
6. Is an IMPORTANT MEDICAL EVENT that satisfies any of the following:
   - May require intervention to prevent items 1 through 5 above.
   - May expose the subject to danger, even though the event is not immediately life threatening or fatal or does not result in hospitalization.
   - Includes any event or synonym described in the Takeda Medically Significant AE List ([Table 10.a](#_bookmark98)).

## Table 10.a Takeda Medically Significant AE List

**Term**

Acute respiratory failure/acute respiratory distress syndrome

Torsade de pointes/ventricular fibrillation/ ventricular tachycardia

Hepatic necrosis Acute liver failure Anaphylactic shock Acute renal failure

Malignant hypertension Pulmonary hypertension

Convulsive seizures Pulmonary fibrosis

Agranulocytosis Confirmed or suspected endotoxin shock

Aplastic anemia Confirmed or suspected transmission of infectious agent by a

Toxic epidermal necrolysis/

medicinal product

Stevens-Johnson syndrome Neuroleptic malignant syndrome/malignant hyperthermia Spontaneous abortion/stillbirth and fetal death

AEs that fulfill 1 or more of the serious criteria above are to be considered SAEs and should be reported and followed up in the same manner (see Section [10.2.8.3](#_bookmark108)).

## AE Procedures

## Assigning Severity/Intensity of AEs

The different categories of severity/intensity are:

Mild: The event is transient and easily tolerated by the subject.

Moderate: The event causes the subject discomfort and interrupts the subject’s usual activities.

Severe: The event causes considerable interference with the subject’s usual activities.

## Assigning Causality of AEs

The relationship of each AE to trial medication(s) will be assessed using the following categories:

Related: An AE that follows a reasonable temporal sequence from administration of a drug (including the course after withdrawal of the drug), or for which possible involvement of the drug can be argued, although factors other than the drug, such as underlying diseases, complications, concomitant drugs and concurrent treatments, may also be responsible.

Not Related: An AE that does not follow a reasonable temporal sequence from administration of a drug and/or that can reasonably be explained by other factors, such as underlying diseases, complications, concomitant drugs, and concurrent treatments.

## Start Date

The start date of the AE is the date that the first signs/symptoms were noted by the subject and/or investigator.

## Stop Date

The stop date of the AE is the date at which the subject recovered, the event resolved but with sequelae or the subject died.

## Frequency

Episodic AEs (eg, headache) or those which occur repeatedly over a period of consecutive days are intermittent. All other events are continuous.

## Action Concerning Study Drug

- Drug withdrawn – a trial medication is stopped due to the particular AE.
- Dose not changed – the particular AE did not require stopping a trial medication.
- Unknown – only to be used if it has not been possible to determine what action has been taken.
- Not applicable – a trial medication was stopped for a reason other than the particular AE eg, the trial has been terminated, the subject died, dosing with trial medication was already stopped before the onset of the AE.

## Outcome

- Recovered/resolved – subject returned to first assessment status with respect to the AE.
- Recovering/resolving – the intensity is lowered by one or more stages: the diagnosis has or signs/symptoms have almost disappeared; the abnormal laboratory value improved, but has not returned to the normal range or to the baseline value; the subject died from a cause other than the particular AE with the condition remaining “recovering/resolving.”
- Not recovered/not resolved – there is no change in the diagnosis, signs or symptoms; the intensity of the diagnosis, signs/symptoms or laboratory value on the last day of the observed trial period has become worse than when it started; is an irreversible congenital anomaly; the subject died from another cause with the particular AE state remaining “Not recovered/not resolved.”
- Resolved/resolved with sequelae – the subject recovered from an acute AE but was left with permanent/significant impairment (eg, recovered from a cardiovascular accident but with some persisting paresis).
- Fatal – an AE that is considered as the cause of death.
- Unknown – the course of the AE cannot be followed up due to hospital change or residence change at the end of the subject’s participation in the trial.

## Collection and Reporting of AEs, SAEs, Special Interest AEs, and Abnormal LFTs

- - - 1. *Collection Period*

Collection of AEs (ie, AEs, SAEs, Special Interest AEs, and Abnormal LFTs) will commence at the time the subject signs the informed consent. Routine collection of AEs will continue throughout the duration of the trial. For subjects who discontinue prior to the administration of trial medication, AEs will be followed until the subject discontinues trial participation.

- - - 1. *Reporting AEs*

At each trial visit, the investigator will assess whether any subjective AEs have occurred. A neutral question, such as “How have you been feeling since your last visit?” may be asked. Subjects may report AEs occurring at any other time during the trial. Subjects experiencing an SAE prior to the first exposure to investigational product must be monitored until the symptoms subside and any clinically relevant changes in laboratory values have returned to Baseline or there is a satisfactory explanation for the change. Nonserious AEs that begin prior to the first exposure to investigational

product, related or unrelated to the trial procedure, need not be followed-up for the purposes of the protocol.

All subjects experiencing AEs, whether considered associated with the use of the trial medication or not, must be monitored until the symptoms subside and any clinically relevant changes in laboratory values have returned to Baseline or until there is a satisfactory explanation for the changes observed. All AEs will be documented in the AE page of the eCRF, whether or not the investigator concludes that the event is related to the drug treatment. The following information will be documented for each event:

- Event term.
- Start and stop date and time.
- Severity.
- Investigator’s opinion of the causal relationship between the event and administration of trial medication(s) (related or not related).
- Action concerning trial medication.
- Outcome of event.
- Seriousness.
  - - 1. *Reporting SAEs*

When an SAE occurs through the AE collection period it should be reported according to the procedure outlined below:

A Takeda SAE form must be completed in English and signed by the investigator immediately or within 24 hours of first onset or notification of the event. The information should be completed as fully as possible but contain, at a minimum:

- A short description of the event and the reason why the event is categorized as serious.
- Subject identification number.
- Investigator’s name.
- Name of the trial medication(s).
- Causality assessment.

The SAE form should be transmitted within 24 hours to the attention of the contact listed in Appendix [14.1.1](#_bookmark135).

Any SAE spontaneously reported to the investigator following the AE collection period should be reported to the Sponsor if considered related to trial participation.

Reporting of SAEs that begin before first administration of investigational product will follow the same procedure for SAEs occurring on treatment.

*SAE Follow-Up*

If information is not available at the time of the first report becomes available at a later date, the investigator should complete a follow-up SAE form or provide other written documentation and fax it immediately within 24 hours of receipt. Copies of any relevant data from the hospital notes (eg, ECGs, laboratory tests, discharge summary, postmortem results) should be sent to the addressee, if requested.

All SAEs should be followed up until resolution or permanent outcome of the event. The timelines and procedure for follow-up reports are the same as those for the initial report.

- - - 1. *Reporting of Abnormal LFTs*

If a subject is noted to have ALT or AST elevated >3 ×ULN on 2 consecutive occasions, the abnormality should be recorded as an AE. In addition, a drug-induced liver injury (DILI) eCRF must be completed providing additional information on relevant recent history, risk factors, clinical signs and symptoms and results of any additional diagnostic tests performed.

If a subject is noted to have ALT or AST >3 ×ULN and total bilirubin >2 ×ULN for which an alternative etiology has not been identified, the event should be recorded as an SAE and reported as per Section [10.2.8.3](#_bookmark108). The investigator must contact the Medical Monitor for discussion of the relevant subject details and possible alternative etiologies, such as acute viral hepatitis A or B or other acute liver disease. Follow-up laboratory tests as described in Section [9.2.10](#_bookmark86) must also be performed. In addition, a DILI eCRF must be completed and transmitted with the Takeda SAE form (as per Section [9.2.10.2](#_bookmark87)).

## Safety Reporting to Investigators, IRBs or IECs, and Regulatory Authorities

The Sponsor will be responsible for reporting all suspected unexpected serious adverse reactions (SUSARs) and any other applicable SAEs to regulatory authorities, investigators and IRBs or IECs, as applicable, in accordance with national regulations in the countries where the trial is conducted. Relative to the first awareness of the event by/or further provision to the Sponsor or Sponsor’s designee, SUSARs will be submitted within 7 days for fatal and life-threatening events and 15 days for other serious events, unless otherwise required by national regulations. The Sponsor will also prepare an expedited report for other safety issues where these might materially alter the current benefit-risk assessment of an investigational medicinal product or that would be sufficient to consider changes in the investigational medicinal products administration or in the overall conduct of the trial. The investigational site also will forward a copy of all expedited reports to his or her IRB or IEC in accordance with national regulations.

## STATISTICAL METHODS

## Statistical and Analytical Plans

A statistical analysis plan will be prepared and finalized prior to unblinding of subject’s treatment sequence assignment. This document will provide further details regarding the definition of analysis variables and analysis methodology to address all study objectives.

## Analysis Sets

- - - 1. *Safety Set*

The safety analysis set will consist of all subjects who are enrolled and received 1 dose of study drug. Subjects in this analysis set will be used for demographic, baseline characteristics, and safety summaries.

- - - 1. *PK Set*

The PK set will consist of all subjects who receive at least 1 dose of study drug and have at least 1 measurable plasma concentration.

- - - 1. *PD Set*

The PD set will consist of all subjects who receive at least 1 dose of study drug and have at least 1 evaluable primary or secondary endpoint.

## Analysis of Demographics and Other Baseline Characteristics

For all randomized subjects, descriptive statistics (N, mean, SD, median, minimum, and maximum) will be generated for continuous demographic variables and baseline characteristics variables (age, height, weight, BMI, etc.) by sequence groups and overall. The number and percentage of subjects in each class of the categorical demographic variables and baseline characteristics variables (sex, ethnicity, race, etc.) will be tabulated by sequence groups and overall. If more than 1 dose is studied, the summary will be by dose, sequence groups and overall.

Demographic variables of screen failure subjects and reasons for screen failures will be summarized overall for subjects who are screened but not enrolled in the trial. Individual demographic characteristics, date of informed consent, and reason for screen failure will be listed.

## PK Analysis

- - - 1. *Concentrations in Plasma*

For each dose of TAK-041, plasma concentrations will be tabulated and descriptive statistics (mean, median, SD, percent coefficient of variation [%CV], minimum and maximum) computed. Individual subject plasma concentration data will be listed.

- - - 1. *Plasma PK Parameters*

For each dose of TAK-041, plasma PK parameter estimates will be tabulated and descriptive statistics (mean, median, SD, %CV, minimum and maximum) computed. In addition, geometric means will be calculated for C_max_ and AUCs. Individual subject plasma PK parameter data will be listed.

Additional statistical analyses of the plasma PK parameters will be performed if appropriate. A more detailed analysis will be presented in the SAP.

## PD Analysis

Observed values along with change from Baseline of PD parameters (primary, secondary and exploratory endpoints) will be summarized by TAK-041 and placebo. Difference from placebo in the observed values and baseline-adjusted values will also be summarized by TAK-041 doses and TAK-041 overall.

A Bayesian GO/NO-GO decision-making approach will be implemented for the evaluation of the effect of TAK-041 for schizophrenia at the interim analysis (after approximately 12 subjects have completed the dosing and Day 1 procedures for both periods including the primary endpoints) and after all subjects complete the study. Using the observed mean (SD) difference in the 2 primary endpoints between the 2 groups (TAK-041 and placebo), the posterior probability that TAK-041 increases the BACS composite score by 2 points over placebo, and the posterior probability that TAK-041 increases the VS activity by at least 0.09 compared to placebo, will be calculated.

Noninformative prior distribution will be used. The criteria for declaring GO and NO-GO will be defined in the SAP.

In addition to estimating the probability of TAK-041 meeting the effect thresholds, analysis of covariance models for repeated measures will be implemented on observed PD endpoints and baseline-adjusted PD endpoints. Baseline PD endpoints will be used as covariate in the model, period, treatment (TAK-041 dose[s], placebo) and sequence will be included as fixed factors in the model. Subject within sequence will be the repeated factor.

Additional analyses will be performed if deemed appropriate.

## Safety Analysis

Safety data will be presented by TAK-041 dose(s), TAK-041 overall, and placebo.

All AEs will be coded using Medical Dictionary of Regulatory Activities (MedDRA). Data will be summarized using preferred term and primary system organ class. TEAEs will be summarized by placebo, each TAK-041 dose level, and TAK-041 overall.

Clinical laboratory variables, vital signs, and ECG parameters will be summarized with descriptive statistics for baseline, postdose, and change from baseline to postdose values by TAK-041 dose(s), TAK-041 overall, and placebo.

The number and percentage of subjects with postdose values meeting Takeda’s criteria for markedly abnormal values for clinical laboratory variables, vital signs, and ECG parameters will be presented by dose.

All summaries will be performed by TAK-041 doses, TAK-041 overall, and placebo. Physical examination findings will be presented in data listings.

## Interim Analysis and Criteria for Early Termination

A small group from the sponsor will remain unblinded to treatment assignment during the study for the purpose of conducting interim analyses of PD effects. In order to minimize any potential unblinding of investigators, these staff will not regularly interact with the investigators or their staff.

A planned unblinded IA of various PD endpoints will be conducted after approximately 12 subjects have completed the Day 14 procedures for both periods including the primary

endpoints. The purpose of the interim PD analysis will be to determine whether to change the dose of TAK-041 for the rest of the subjects, or continue with their current TAK-041 dose. The Bayesian decision rule will be specified in the SAP before the data are unblinded for the IA. In addition, a decision to switch to the low dose may be made based on safety, tolerability and PK data of the high dose.

## Determination of Sample Size

Up to 32 subjects are planned to be randomized equally to 2 treatment sequences ([Table 6.a](#_bookmark32)) to ensure 24 subjects complete the trial.

Both primary endpoints will be used at the IA and the end of the study for decision making. A Bayesian method will be used to calculate the posterior probability of the endpoints meeting the predefined criteria (see the SAP) using data from TAK-041 and placebo. The probability of making a ‘GO’ decision based on the predefined ‘GO’ criteria with 24 subjects is at least 70% when the true TAK-041 effect on either endpoint is clinically meaningful (see the SAP). This sample size will also keep the type 1 error rate no more than 30% when the true drug effect is minimal or the same as placebo. The SD for the BACS composite score is assumed to be

9.30 points. The SD for the VS activation is assumed to be 0.360. Independence between these 2 endpoints was assumed in the sample size calculation.

Noninformative prior distribution was used in the above probability calculation. Allowing for

8 dropouts (due to the long washout interval), up to 32 subjects are planned to be enrolled in order to have 24 completers for this trial.

## QUALITY CONTROL AND QUALITY ASSURANCE

## Study-Site Monitoring Visits

Monitoring visits to the trial sites will be made periodically during the study to ensure that all aspects of the protocol are followed. Source documents will be reviewed for verification of data recorded on the eCRFs. Source documents are defined as original documents, data, and records. The investigator and trial sites guarantee access to source documents by the Sponsor or its designee (contract research organization [CRO]) and by the IRB or IEC.

All aspects of the trial and its documentation will be subject to review by the Sponsor or the Sponsor’s designee (as long as blinding is not jeopardized), including but not limited to the Investigator’s Binder, study drug, subject medical records, informed consent documentation, and review of eCRFs and associated source documents. It is important that the investigator and other trial personnel are available during the monitoring visits and that sufficient time is devoted to the process.

## Protocol Deviations

The investigator should not deviate from the protocol, except where necessary to eliminate an immediate hazard to trial subjects. Should other unexpected circumstances arise that will require deviation from protocol-specified procedures, the investigator should consult with the sponsor or designee (and IRB or IEC, as required) to determine the appropriate course of action. There will be no exemptions (a prospectively approved deviation) from the inclusion or exclusion criteria.

Significant deviations include, but are not limited to, those that involve fraud or misconduct, increase the health risk to the subject, or confound interpretation of primary trial assessment.

## Quality Assurance Audits and Regulatory Agency Inspections

The trial sites also may be subject to quality assurance audits by the Sponsor or designees. In this circumstance, the Sponsor-designated auditor will contact the sites in advance to arrange an auditing visit. The auditor may ask to visit the facilities where laboratory samples are collected, where the medication is stored and prepared, and any other facility used during the study. In addition, there is the possibility that this trial may be inspected by regulatory agencies, including those of foreign governments (eg, the Food and Drug Administration [FDA], the United Kingdom Medicines and Healthcare Products Regulatory Agency, the Pharmaceuticals and Medical Devices Agency of Japan). If any of the trial sites are contacted for an inspection by a regulatory body, the Sponsor should be notified immediately. The investigator guarantees access for quality assurance auditors to all trial documents as described in Section [12.1.](#_bookmark121)

## ETHICAL ASPECTS OF THE STUDY

This trial will be conducted with the highest respect for the individual participants (ie, subjects) according to the protocol, the ethical principles that have their origin in the Declaration of Helsinki, and the International Conference on Harmonization (ICH) Harmonized Tripartite Guideline for GCP. Each investigator will conduct the trial according to applicable local or regional regulatory requirements and align his or her conduct in accordance with the “Responsibilities of the Investigator” that are listed in [Appendix A.](#_bookmark161) The principles of Helsinki are addressed through the protocol and through appendices containing requirements for informed consent and investigator responsibilities.

## IRB and/or IEC Approval

IRBs and IECs must be constituted according to the applicable state and federal/local requirements of each participating region. The Sponsor or designee will require documentation noting all names and titles of members who make up the respective IRB or IEC. If any member of the IRB or IEC has direct participation in this trial, written notification regarding his or her abstinence from voting must also be obtained. Those Americas sites unwilling to provide names and titles of all members due to privacy and conflict of interest concerns should instead provide a Federal Wide Assurance Number or comparable number assigned by the Department of Health and Human Services.

The Sponsor or designee will supply relevant documents for submission to the respective IRB or IEC for the protocol’s review and approval. This protocol, the Investigator’s Brochure, a copy of the informed consent form, and, if applicable, subject recruitment materials and/or advertisements and other documents required by all applicable laws and regulations, must be submitted to a central or local IRB or IEC for approval. The IRB’s or IEC’s written approval of the protocol and subject informed consent must be obtained and submitted to the Sponsor or designee before commencement of the trial (ie, before shipment of the Sponsor-supplied drug or trial-specific screening activity). The IRB or IEC approval must refer to the trial by exact protocol title, number, and version date; identify versions of other documents (eg, informed consent form) reviewed; and state the approval date. The Sponsor will ship drug/notify sites once the Sponsor has confirmed the adequacy of site regulatory documentation and, when applicable, the Sponsor has received permission from competent authority to begin the trial.

Sites must adhere to all requirements stipulated by their respective IRB or IEC. This may include notification to the IRB or IEC regarding protocol amendments, updates to the informed consent form, recruitment materials intended for viewing by subjects, local safety reporting requirements, reports and updates regarding the ongoing review of the trial at intervals specified by the respective IRB or IEC, and submission of the investigator’s final status report to IRB or IEC. All IRB and IEC approvals and relevant documentation for these items must be provided to the Sponsor or its designee.

Subject incentives should not exert undue influence for participation. Payments to subjects must be approved by the IRB or IEC and Sponsor.

## Subject Information, Informed Consent, and Subject Authorization

Written consent documents will embody the elements of informed consent as described in the Declaration of Helsinki and the ICH Guidelines for GCP and will be in accordance with all applicable laws and regulations. The informed consent form, subject authorization form (if applicable), and subject information sheet (if applicable) describe the planned and permitted uses, transfers, and disclosures of the subject’s personal and personal health information for purposes of conducting the trial. The informed consent form and the subject information sheet (if applicable) further explain the nature of the trial, its objectives, and potential risks and benefits, as well as the date informed consent is given. The informed consent form will detail the requirements of the participant and the fact that he or she is free to withdraw at any time without giving a reason and without prejudice to his or her further medical care.

The investigator is responsible for the preparation, content, and IRB or IEC approval of the informed consent form and, if applicable, the subject authorization form. The informed consent form, subject authorization form (if applicable), and subject information sheet (if applicable) must be approved by both the IRB or IEC and the Sponsor prior to use.

The informed consent form, subject authorization form (if applicable), and subject information sheet (if applicable) must be written in a language fully comprehensible to the prospective subject. It is the responsibility of the investigator to explain the detailed elements of the informed consent form, subject authorization form (if applicable), and subject information sheet (if applicable) to the subject. Information should be given in both oral and written form whenever possible and in the manner deemed appropriate by the IRB or IEC. In the event the subject is not capable of rendering adequate written informed consent, then the subject’s legally acceptable representative may provide such consent for the subject in accordance with applicable laws and regulations.

The subject, or the subject’s legally acceptable representative, must be given ample opportunity to:

(1) inquire about details of the trial, and (2) decide whether or not to participate in the trial. If the subject, or the subject’s legally acceptable representative, determines he or she will participate in the trial, then the informed consent form and subject authorization form (if applicable) must be signed and dated by the subject, or the subject’s legally acceptable representative, at the time of consent and prior to the subject entering into the trial. The subject or the subject’s legally acceptable representative should be instructed to sign using their legal names, not nicknames, using blue or black ballpoint ink. The investigator must also sign and date the informed consent form and subject authorization (if applicable) at the time of consent and prior to subject entering into the trial; however, the Sponsor may allow a designee of the investigator to sign to the extent permitted by applicable law.

Once signed, the original informed consent form, subject authorization form (if applicable), and subject information sheet (if applicable) will be stored in the investigator’s site file. The investigator must document the date the subject signs the informed consent in the subject’s medical record. Copies of the signed informed consent form, the signed subject authorization form (if applicable), and subject information sheet (if applicable) shall be given to the subject.

All revised informed consent forms must be reviewed and signed by relevant subjects or the relevant subject’s legally acceptable representative in the same manner as the original informed consent. The date the revised consent was obtained should be recorded in the subject’s medical record, and the subject should receive a copy of the revised informed consent form.

Subjects who consented and provided a PGx sample for DNA and RNA analysis can withdraw their consent and request disposal of a stored sample at any time prior to analysis. Notify Sponsor of consent withdrawal.

## Subject Confidentiality

The Sponsor and designees affirm and uphold the principle of the subject’s right to protection against invasion of privacy. Throughout this trial, a subject’s source data will only be linked to the Sponsor’s clinical trial database or documentation via a unique identification number. As permitted by all applicable laws and regulations, limited subject attributes, such as sex, age, or date of birth, and subject initials may be used to verify the subject and accuracy of the subject’s unique identification number.

To comply with ICH Guidelines for GCP and to verify compliance with this protocol, the Sponsor requires the investigator to permit its monitor or designee’s monitor, representatives from any regulatory authority (eg, FDA, Medicines and Healthcare products Regulatory Agency, Pharmaceuticals and Medical Devices Agency), the Sponsor’s designated auditors, and the appropriate IRBs and IECs to review the subject’s original medical records (source data or documents), including, but not limited to, laboratory test result reports, ECG reports, admission and discharge summaries for hospital admissions occurring during a subject’s trial participation, and autopsy reports. Access to a subject’s original medical records requires the specific authorization of the subject as part of the informed consent process (see Section [13.2](#_bookmark126)).

Copies of any subject source documents that are provided to the Sponsor must have certain personally identifiable information removed (ie, subject name, address, and other identifier fields not collected on the subject’s eCRF).

## Publication, Disclosure, and Clinical Trial Registration Policy

## Publication and Disclosure

The investigator is obliged to provide the Sponsor with complete test results and all data derived by the investigator from the trial. During and after the trial, only the Sponsor may make trial information available to other trial investigators or to regulatory agencies, except as required by law or regulation. Except as otherwise allowable in the clinical trial site agreement, any public disclosure (including publicly accessible websites) related to the protocol or trial results, other than trial recruitment materials and/or advertisements, is the sole responsibility of the Sponsor.

The Sponsor may publish any data and information from the trial (including data and information generated by the investigator) without the consent of the investigator. Manuscript authorship for any peer-reviewed publication will appropriately reflect contributions to the production and review of the document. All publications and presentations must be prepared in accordance with

this section and the Clinical Study Site Agreement. In the event of any discrepancy between the protocol and the Clinical Study Site Agreement, the Clinical Study Site Agreement will prevail.

## Clinical Trial Registration

In order to ensure that information on clinical trials reaches the public in a timely manner and to comply with applicable laws, regulations and guidance, Takeda will, at a minimum, register all interventional clinical trials it Sponsors anywhere in the world on ClinicalTrials.gov and/or other publicly accessible websites before start of trial, as defined in Takeda Policy/Standard. Takeda contact information, along with investigator’s city, state (for Americas investigators), country, and recruiting status will be registered and available for public viewing.

For some registries, Takeda will assist callers in locating trial sites closest to their homes by providing the investigator name, address, and phone number to the callers requesting trial information. Once subjects receive investigator contact information, they may call the site requesting enrollment into the trial. The investigative sites are encouraged to handle the trial inquiries according to their established subject screening process. If the caller asks additional questions beyond the topic of trial enrollment, they should be referred to the Sponsor.

Any investigator who objects to the Sponsor providing this information to callers must provide the Sponsor with a written notice requesting that their information not be listed on the registry site.

## Clinical Trial Results Disclosure

Takeda will post the results of clinical trials on ClinicalTrials.gov or other publicly accessible websites, as required by Takeda Policy/Standard, applicable laws and/or regulations.

## Insurance and Compensation for Injury

Each subject in the trial must be insured in accordance with the regulations applicable to the site where the subject is participating. If a local underwriter is required, then the Sponsor or Sponsor’s designee will obtain clinical trial insurance against the risk of injury to trial subjects. Refer to the trial site agreement regarding the Sponsor’s policy on subject compensation and treatment for injury. If the investigator has questions regarding this policy, he or she should contact the Sponsor or Sponsor’s designee.

## ADMINISTRATIVE AND REFERENCE INFORMATION

## Administrative Information

## Study Contact Information

**Contact Type / Role Contact**

Serious adverse event and pregnancy reporting Pharmacovigilance

Takeda Development Centre Europe Ltd. Email: [eupv@tgrd.com](mailto:eupv@tgrd.com)

## Investigator Agreement

I confirm that I have read and that I understand this protocol, the Investigator’s Brochure, package insert and any other product information provided by the Sponsor. I agree to conduct this trial in accordance with the requirements of this protocol and also to protect the rights, safety, privacy, and well-being of trial subjects in accordance with the following:

- The ethical principles that have their origin in the Declaration of Helsinki.
- International Conference on Harmonization, E6 Good Clinical Practice: Consolidated Guideline.
- All applicable laws and regulations, including, without limitation, data privacy laws and regulations.
- Regulatory requirements for reporting serious adverse events defined in Section [10.2.9](#_bookmark110) of this protocol.
- Terms outlined in the trial site agreement.
- Responsibilities of the Investigator ([Appendix A](#_bookmark161)).

I further authorize that my personal information may be processed and transferred in accordance with the uses contemplated in [Appendix C](#_bookmark163) of this protocol.

Signature of Investigator Date

Investigator Name (print or type)

Investigator’s Title

Location of Facility (City, State/Province)

Location of Facility (Country)

## Study-Related Responsibilities

The Sponsor will perform all trial-related activities with the exception of those identified in the Study-Related Responsibilities template. The vendors identified for specific trial-related activities will perform these activities in full or in partnership with the Sponsor.

## List of Abbreviations

**Term Definition**

%CV percent coefficient of variation

AE adverse event

ALP alkaline phosphatase

ALT alanine aminotransferase

ASL arterial spin labeling

AST aspartate aminotransferase

AUC area under the plasma concentration-time curve

BACS Brief Assessment of Cognition in Schizophrenia

BMI body mass index

BNSS Brief Negative Symptom Scale

BOLD blood-oxygen-level-dependent

CGI Clinical Global Impression

CGI-I Clinical Global Impression - Improvement

CGI-S Clinical Global Impression - Severity

C_max_ maximum observed plasma concentration

CPAP clinical pharmacology analysis plan

CRO contract research organization

C-SSRS Columbia-Suicide Severity Rating Scale

DILI drug-induced liver injury

DSM-5 Diagnostic and Statistical Manual of Mental Disorders-fifth edition

ECG electrocardiogram

eCRF electronic case report form

EEfRT Effort Expenditure For Rewards Task

FDA Food and Drug Administration

fMRI functional magnetic resonance imaging

FSH follicle-stimulating hormone

GCP Good Clinical Practice

GGT γ-glutamyl transferase

GPR139 G protein-coupled receptor 139

HBsAg hepatitis B surface antigen

HCV hepatitis C virus

HIV human immunodeficiency virus

IA interim analysis

ICC intra-class correlation

ICH International Conference of Harmonisation

IEC independent ethics committee

IRB institutional review board

IRT interactive response technology

**Term Definition**

LFT liver function tests

MedDRA Medical Dictionary of Regulatory Activities

MID Monetary Incentive Delay

MINI Mini International Neuropsychiatric Interview

MRD multiple-rising dose

MRI magnetic resonance imaging

NSFS negative symptom factor score

OTC over-the-counter

PANSS Positive and Negative Syndrome Scale

PD pharmacodynamic(s)

PGx pharmacogenomic(s)

PK pharmacokinetic(s)

QTcF QT interval with Fridericia correction method

rs-fMRI resting state functional magnetic resonance imaging

SAE serious adverse event

SAP statistical analysis plan

SRD single-rising dose

SUSAR suspected unexpected serious adverse reaction

t-fMRI 2-task functional magnetic resonance imaging

t_1/2z_ terminal disposition phase half-life

TBILI total bilirubin

TEAE treatment-emergent adverse event

Tween/MC vehicle 0.5% Tween 80 in 0.5% methylcellulose vehicle ULN upper limit of normal

VS ventral striatum

## DATA HANDLING AND RECORDKEEPING

The full details of procedures for data handling will be documented in the Data Management Plan. AEs, medical history, and concurrent conditions will be coded using the MedDRA. Drugs will be coded using the World Health Organization Drug Dictionary.

## CRFs (Electronic and Paper)

Completed eCRFs are required for each subject who signs an informed consent.

The Sponsor or its designee will supply investigative sites with access to eCRFs. The Sponsor will make arrangements to train appropriate site staff in the use of the eCRF. These forms are used to transmit the information collected in the performance of this trial to the Sponsor and regulatory authorities. eCRFs must be completed in English. Data are transcribed directly onto eCRFs.

After completion of the entry process, computer logic checks will be run to identify items, such as inconsistent dates, missing data, and questionable values. Queries may be issued by Takeda personnel (or designees) and will be answered by the site.

Corrections are recorded in an audit trail that captures the old information, the new information, identification of the person making the correction, the date the correction was made, and the reason for change. Reasons for significant corrections should additionally be included.

The principal investigator must review the eCRFs for completeness and accuracy and must sign and date the appropriate eCRFs as indicated. Furthermore, the investigator must retain full responsibility for the accuracy and authenticity of all data entered on the eCRFs.

After the lock of the clinical trial database, any change of, modification of, or addition to the data on the eCRFs should be made by the investigator with use of change and modification records of the eCRFs. The principal investigator must review the data change for completeness and accuracy, and must sign and date.

eCRFs will be reviewed for completeness and acceptability at the trial site during periodic visits by trial monitors. The Sponsor or its designee will be permitted to review the subject’s medical and hospital records pertinent to the trial to ensure accuracy of the eCRFs. The completed eCRFs are the sole property of the Sponsor and should not be made available in any form to third parties, except for authorized representatives of appropriate governmental health or regulatory authorities, without written permission of the Sponsor.

## Record Retention

The investigator agrees to keep the records stipulated in Section [15.1](#_bookmark140) and those documents that include (but are not limited to) the trial-specific documents, the identification log of all participating subjects, medical records, temporary media such as thermal sensitive paper, source worksheets, all original signed and dated informed consent forms, subject authorization forms regarding the use of personal health information (if separate from the informed consent forms), electronic copy of eCRFs, including the audit trail, and detailed records of drug disposition to enable evaluations or audits from regulatory authorities, the Sponsor or its designees. Any source

documentation printed on degradable thermal sensitive paper should be photocopied by the site and filed with the original in the subject’s chart to ensure long-term legibility. Furthermore, ICH E6 Section 4.9.5 requires the investigator to retain essential documents specified in ICH E6 (Section 8) until at least 2 years after the last approval of a marketing application for a specified drug indication being investigated or, if an application is not approved, until at least 2 years after the investigation is discontinued and regulatory authorities are notified. In addition, ICH E6 Section 4.9.5 states that the trial records should be retained until an amount of time specified by applicable regulatory requirements or for a time specified in the Clinical Study Site Agreement between the investigator and Sponsor.

Refer to the Clinical Study Site Agreement for the Sponsor’s requirements on record retention. The investigator and the head of the institution should contact and receive written approval from the Sponsor before disposing of any such documents.

## 16.0 REFERENCES

1. van Kerkhof LW, Damsteegt R, Trezza V, Voorn P, Vanderschuren LJ. Functional integrity of the habenula is necessary for social play behaviour in rats. Eur J Neurosci

2013;38(10):3465-75.

1. Lecourtier L, Neijt HC, Kelly PH. Habenula lesions cause impaired cognitive performance in rats: implications for schizophrenia. Eur J Neurosci 2004;19(9):2551-60.
2. Castellani CA, Awamleh Z, Melka MG, O'Reilly RL, Singh SM. Copy number variation distribution in six monozygotic twin pairs discordant for schizophrenia. Twin Res Hum Genet 2014;17(2):108-20.
3. American Psychiatric Association. Diagnostic and Statistical Manual of Mental Disorders (DSM-5). 5th ed. Arlington, VA: American Psychiatric Association; 2013.
4. Reddy LF, Horan WP, Barch DM, Buchanan RW, Dunayevich E, Gold JM, et al. Effort-Based Decision-Making Paradigms for Clinical Trials in Schizophrenia: Part 1-Psychometric Characteristics of 5 Paradigms. Schizophr Bull 2015;41(5):1045-54.
5. Horan WP, Reddy LF, Barch DM, Buchanan RW, Dunayevich E, Gold JM, et al. Effort-Based Decision-Making Paradigms for Clinical Trials in Schizophrenia: Part 2-External Validity and Correlates. Schizophr Bull 2015;41(5):1055-65.
6. Strauss GP, Whearty KM, Morra LF, Sullivan SK, Ossenfort KL, Frost KH. Avolition in schizophrenia is associated with reduced willingness to expend effort for reward on a Progressive Ratio task. Schizophr Res 2016;170(1):198-204.
7. Barch DM, Treadway MT, Schoen N. Effort, anhedonia, and function in schizophrenia: reduced effort allocation predicts amotivation and functional impairment. J Abnorm Psychol 2014;123(2):387-97.
8. Plichta MM, Schwarz AJ, Grimm O, Morgen K, Mier D, Haddad L, et al. Test-retest reliability of evoked BOLD signals from a cognitive-emotive fMRI test battery. Neuroimage 2012;60(3):1746-58.
9. Ceaser AE, Goldberg TE, Egan MF, McMahon RP, Weinberger DR, Gold JM. Set-shifting ability and schizophrenia: a marker of clinical illness or an intermediate phenotype? Biol Psychiatry 2008;64(9):782-8.
10. Hampshire A, Owen AM. Fractionating attentional control using event-related fMRI. Cereb Cortex 2006;16(12):1679-89.
11. Keefe RS, Goldberg TE, Harvey PD, Gold JM, Poe MP, Coughenour L. The Brief Assessment of Cognition in Schizophrenia: reliability, sensitivity, and comparison with a standard neurocognitive battery. Schizophr Res 2004;68(2-3):283-97.
12. Atkins AS, Tseng T, Vaughan A, Twamley EW, Harvey P, Patterson T, et al. Validation of the tablet-administered Brief Assessment of Cognition (BAC App). Schizophr Res

2017;181:100-6.

1. Bland AR, Roiser JP, Mehta MA, Schei T, Boland H, Campbell-Meiklejohn DK, et al. EMOTICOM: A neuropsychological test battery to evaluate emotion, motivation, impulsivity, and social cognition. Front Behav Neurosci 2016;10:25.
2. Kay SR, Fiszbein A, Opler LA. The positive and negative syndrome scale (PANSS) for schizophrenia. Schizophr Bull 1987;13(2):261-76.
3. Kay SR, Opler LA, Lindenmayer JP. Reliability and validity of the positive and negative syndrome scale for schizophrenics. Psychiatry Res 1988;23(1):99-110.
4. Kirkpatrick B, Strauss GP, Nguyen L, Fischer BA, Daniel DG, Cienfuegos A, et al. The brief negative symptom scale: psychometric properties. Schizophr Bull 2011;37(2):300-5.
5. Guy W. Clinical Global Impression (CGI), ECDEU Version. In: ECDEU Assessment Manual for Psychopharmacology. Revised ed. Rockville, MD: U.S. Dept. of Health, Education, and Welfare; 1976; DHEW publication no. (ADM) 76-338, p. 218-22.
6. Kern RS, Penn DL, Lee J, Horan WP, Reise SP, Ochsner KN, et al. Adapting social neuroscience measures for schizophrenia clinical trials, Part 2: trolling the depths of psychometric properties. Schizophr Bull 2013;39(6):1201-10.

## 17.0 APPENDICES

**Appendix A Responsibilities of the Investigator**

Clinical research studies sponsored by the Sponsor are subject to ICH GCP and all the applicable local laws and regulations. The responsibilities imposed on investigators by the FDA are summarized in the “Statement of Investigator” (Form FDA 1572), which must be completed and signed before the investigator may participate in this trial.

The investigator agrees to assume the following responsibilities by signing a Form FDA 1572:

1. Conduct the trial in accordance with the protocol.
2. Personally conduct or supervise the staff that will assist in the protocol.
3. If the investigator/institution retains the services of any individual or party to perform

trial-related duties and functions, the investigator/institution should ensure that this individual or party is qualified to perform those trial-related duties and functions and should implement procedures to ensure the integrity of the trial-related duties and functions performed and any data generated.

1. Ensure that trial-related procedures, including trial-specific (nonroutine/nonstandard panel) screening assessments are NOT performed on potential subjects, prior to the receipt of written approval from relevant governing bodies/authorities.
2. Ensure that all colleagues and employees assisting in the conduct of the trial are informed of these obligations.
3. Secure prior approval of the trial and any changes by an appropriate IRB/IEC that conform to ICH and local regulatory requirements.
4. Ensure that the IRB/IEC will be responsible for initial review, continuing review, and approval of the protocol. Promptly report to the IRB/IEC all changes in research activity and all anticipated risks to subjects. Make at least yearly reports on the progress of the trial to the IRB/IEC, and issue a final report within 3 months of trial completion.
5. Ensure that requirements for informed consent as outlined in ICH and local regulations are met.
6. Obtain valid informed consent from each subject who participates in the trial, and document the date of consent in the subject’s medical chart. Valid informed consent is the most current version approved by the IRB/IEC. Each informed consent form should contain a subject authorization section that describes the uses and disclosures of a subject’s personal information (including personal health information) that will take place in connection with the trial. If an informed consent form does not include such a subject authorization, then the investigator must obtain a separate subject authorization form from each subject or the subject’s legally acceptable representative.
7. Prepare and maintain adequate case histories of all persons entered into the trial, including eCRFs, hospital records, laboratory results, etc, and maintain these data for a minimum of

2 years following notification by the Sponsor that all investigations have been discontinued or that the regulatory authority has approved the marketing application. The investigator should contact and receive written approval from the Sponsor before disposing of any such documents.

1. Allow possible inspection and copying by the regulatory authority of GCP-specified essential documents.
2. Maintain current records of the receipt, administration, and disposition of Sponsor-supplied drugs, and return all unused Sponsor-supplied drugs to the Sponsor.
3. Report adverse reactions to the Sponsor promptly. In the event of an SAE, notify the Sponsor within 24 hours.

## Appendix B Elements of the Subject Informed Consent

In seeking informed consent, the following information shall be provided to each subject:

1. A statement that the trial involves research.
2. An explanation of the purposes of the research.
3. The expected duration of the subject’s participation.
4. A description of the procedures to be followed, including invasive procedures.
5. The identification of any procedure that is experimental.
6. The estimated number of subjects involved in the trial.
7. A description of the subject’s responsibilities.
8. A description of the conduct of the trial.
9. A statement describing the treatment(s) and the probability for random assignment to each treatment.
10. A description of the possible side effects of the treatment that the subject may receive.
11. A description of any reasonably foreseeable risks or discomforts to the subject and, when applicable, to an embryo, fetus, or nursing infant.
12. A description of any benefits to the subject or to others that reasonably may be expected from the research. When there is no intended clinical benefit to the subject, the subject should be made aware of this.
13. Disclosures of appropriate alternative procedures or courses of treatment, if any, that might be advantageous to the subject and their important potential risks and benefits.
14. A statement describing the extent to which confidentiality of records identifying the subject will be maintained, and a note of the possibility that regulatory agencies, auditor(s), IRB/IEC, and the monitor may inspect the records. By signing a written informed consent form, the subject or the subject’s legally acceptable representative is authorizing such access.
15. For research involving more than minimal risk, an explanation as to whether any compensation and an explanation as to whether any medical treatments are available if injury occurs and, if so, what they consist of or where further information may be obtained.
16. The anticipated prorated payment(s), if any, to the subject for participating in the trial.
17. The anticipated expenses, if any, to the subject for participating in the trial.
18. An explanation of whom to contact for answers to pertinent questions about the research (investigator), subject’s rights, and IRB/IEC and whom to contact in the event of a research-related injury to the subject.
19. A statement that participation is voluntary, that refusal to participate will involve no penalty or loss of benefits to which the subject otherwise is entitled, and that the subject may discontinue

participation at any time without penalty or loss of benefits to which the subject is otherwise entitled.

1. The consequences of a subject’s decision to withdraw from the research and procedures for orderly termination of participation by the subject.
2. A statement that the subject or the subject’s legally acceptable representative will be informed in a timely manner if information becomes available that may be relevant to the subject’s willingness to continue participation in the trial.
3. A statement that results of PGx analysis will not be disclosed to an individual, unless prevailing laws require the Sponsor to do so.
4. The foreseeable circumstances or reasons under which the subject’s participation in the trial may be terminated.
5. A written subject authorization (either contained within the informed consent form or provided as a separate document) describing to the subject the contemplated and permissible uses and disclosures of the subject’s personal information (including personal health information) for purposes of conducting the trial. The subject authorization must contain the following statements regarding the uses and disclosures of the subject’s personal information:
   - that personal information (including personal health information) may be processed by or transferred to other parties in other countries for clinical research and safety reporting purposes, including, without limitation, to the following: (1) Takeda, its affiliates, and licensing partners; (2) business partners assisting Takeda, its affiliates, and licensing partners; (3) regulatory agencies and other health authorities; and (4) IRBs/IECs.
   - that it is possible that personal information (including personal health information) may be processed and transferred to countries that do not have data protection laws that offer subjects the same level of protection as the data protection laws within this country; however, Takeda will make every effort to keep your personal information confidential, and your name will not be disclosed outside the clinic unless required by law.
   - that personal information (including personal health information) may be added to Takeda’s research databases for purposes of developing a better understanding of the safety and effectiveness of the trial medication(s), studying other therapies for patients, developing a better understanding of disease, and improving the efficiency of future clinical studies.
   - that subjects agree not to restrict the use and disclosure of their personal information (including personal health information) upon withdrawal from the trial to the extent that the restricted use or disclosure of such information may impact the scientific integrity of the research.
   - that the subject’s identity will remain confidential in the event that trial results are published.
6. Male subjects must use adequate contraception and female subjects must use 2 highly effective methods of contraception (as defined in the informed consent) from signing of the informed consent form, throughout the duration of the study, and for either 145 days (males) or 85 days (females) after last dose of trial medication. If the partner or wife of the subject is found to be pregnant during the trial, the investigator will offer the subject the choice to receive unblinded treatment information.
7. A statement that clinical trial information from this trial will be publicly disclosed in a publicly accessible website, such as ClinicalTrials.gov.

## Appendix C Investigator Consent to Use of Personal Information

Takeda will collect and retain personal information of investigator, including his or her name, address, and other personally identifiable information. In addition, investigator’s personal information may be transferred to other parties located in countries throughout the world (eg, the United Kingdom, United States, and Japan), including the following:

- Takeda, its affiliates, and licensing partners.
- Business partners assisting Takeda, its affiliates, and licensing partners.
- Regulatory agencies and other health authorities.
- IRBs and IECs.

Investigator’s personal information may be retained, processed, and transferred by Takeda and these other parties for research purposes including the following:

- Assessment of the suitability of investigator for the trial and/or other clinical studies.
- Management, monitoring, inspection, and audit of the trial.
- Analysis, review, and verification of the trial results.
- Safety reporting and pharmacovigilance relating to the trial.
- Preparation and submission of regulatory filings, correspondence, and communications to regulatory agencies relating to the trial.
- Preparation and submission of regulatory filings, correspondence, and communications to regulatory agencies relating to other medications used in other clinical studies that may contain the same chemical compound present in the trial medication.
- Inspections and investigations by regulatory authorities relating to the trial.
- Self-inspection and internal audit within Takeda, its affiliates, and licensing partners.
- Archiving and audit of trial records.
- Posting investigator site contact information, trial details and results on publicly accessible clinical trial registries, databases, and websites.

Investigator’s personal information may be transferred to other countries that do not have data protection laws that offer the same level of protection as data protection laws in investigator’s own country.

Investigator acknowledges and consents to the use of his or her personal information by Takeda and other parties for the purposes described above.

**Appendix D Pregnancy and Contraception Contraception and Pregnancy Avoidance Procedure** *Female Subjects and their Male Partners*

From signing of informed consent, throughout the duration of the trial, and for 85 days after last dose of study drug, female subjects of childbearing potential who are sexually active with a nonsterilized male partner must use 2 highly effective methods of contraception (from the list below).

In addition they must be advised not to donate ova during this period.

A woman is considered a woman of childbearing potential, ie, fertile, following menarche and until becoming postmenopausal unless permanently sterile. Permanent sterilization methods include hysterectomy and bilateral oophorectomy. A postmenopausal state is defined as no menses for 12 months without an alternative medical cause. A high FSH level in the postmenopausal range (FSH >40 IU/L) may be used to confirm a postmenopausal state in younger women (eg, those <45 years old) or women who are not using hormonal contraception or hormonal replacement therapy. However, in the absence of 12 months of amenorrhea, a single FSH measurement is insufficient.

*Male Subjects and Their Female Partners*

From signing of informed consent, throughout the duration of the study, and for 145 days after the last dose of study drug, nonsterilized** male subjects who are sexually active with a female partner of childbearing potential must use barrier contraception (eg, condom with or without spermicidal cream or jelly). In addition, they must be advised not to donate sperm during this period. Females of childbearing potential who are partners of male subjects are also advised to use additional contraception as shown in the list containing highly effective contraception below.

Sterilized males should be at least 1 year post–bilateral vasectomy and have confirmed that they have obtained documentation of the absence of sperm in the ejaculate or have had bilateral orchidectomy.

The following procedures apply for contraception and pregnancy avoidance:

1. Highly effective methods of contraception are defined as “those, alone or in combination, that result in a low failure rate (ie, less than 1% failure rate per year when used consistently and correctly)”:

- Nonhormonal methods:
  - Intrauterine device (IUD).
  - Bilateral tubal occlusion.
  - Vasectomized partner (provided that partner is the sole sexual partner of the trial participant and that the vasectomized partner has received medical assessment of the surgical success).
  - True sexual abstinence, only if this is in line with the preferred and usual lifestyle of the subject. True abstinence is defined as refraining from heterosexual intercourse during the entire period of the trial, from 1 month prior to the first dose until 167 days after last dose.

1. Unacceptable methods of contraception are:

- Hormonal methods.
- Periodic abstinence (eg, calendar, ovulation, symptothermal, postovulation methods).
- Spermicides only.
- Withdrawal.
- No method at all.
- Use of female and male condoms together.
- Cap/diaphragm/sponge without spermicide and without condom.

1. Subjects will be provided with information on highly effective methods of contraception as part of the subject informed consent process and will be asked to sign a consent form stating that they understand the requirements for avoidance of pregnancy, and sperm donation during the course of the trial.
2. Subjects will receive continued guidance with respect to the avoidance of pregnancy and sperm donation as part of the trial procedures. Such guidance should include a reminder of the following:

- Contraceptive requirements of the trial.
- Reasons for use of barrier methods (ie, condom) in males with pregnant partners.
- Assessment of subject compliance through questions such as:
  - Have you used the contraception consistently and correctly since the last visit?
  - Have you forgotten to use contraception since the last visit?
  - Are your menses late (even in women with irregular or infrequent menstrual cycles a pregnancy test must be performed if the answer is “yes”)?
  - Is there a chance you could be pregnant?

1. In addition to a negative serum pregnancy test at Screening, female subjects of childbearing potential must also have confirmed menses in the month before first dosing (no delayed menses), a negative urine pregnancy test at time points specified in the Schedule of Procedures, [Table 3.a.](#_bookmark16)

## Appendix E Detailed Description of Amendments to Text

The primary section(s) of the protocol affected by the changes in Amendment No. 04 are indicated. The corresponding text has been revised throughout the protocol.

**Change** [**1**](#_bookmark2)**:** [Modified rationale and potential range for study drug dose level..](#_bookmark2)

The primary change occurs in Section [6.2 Rationale for Study Design, Dose, and Endpoints.](#_bookmark33)

Initial wording:

The crossover design has been selected in order to reduce sample size (N = 24), as well as minimize variability in the endpoints. TAK-041 has been evaluated in a single-rising dose (SRD) trial in healthy volunteers at doses up to 40 mg crystalline suspension. During the SRD trial, PK blood samples were collected for 146 days following the 40 mg TAK-041 dose. Preliminary PK data indicated that TAK-041 has a mean terminal elimination half-life (t_1/2_) of approximately 11 days. Therefore, 35 + 7 days between the 2 treatment periods is sufficient to ensure that there is no drug carryover effect. At the end of 35 +7 days, the probability of exceeding the “effect” cut-off is less than 2%, with 17 subjects out of 1000 expected to have plasma concentrations of TAK-041 above 132.5 ng/mL.

## Section 6.2 Rationale for Dose

Up to 2 doses of TAK-041 will be used in this study. The first dose examined in this study will be a single 40 mg dose of TAK-041 crystalline suspension (or matching placebo) administered on Day 1 of each treatment period according to the sequence group assigned through the IA. This dose is the highest dose that has been studied thus far in the ongoing TAK-041-1001 first-in-human Phase 1 study. The clinical exposure achieved at this dose is aligned with predicted human exposure of the highest efficacious dose (1 mg/kg) tested in nonclinical in vivo models of anhedonia and asociality. As discussed in Section [4.3,](#_bookmark20) this dose was found to be well tolerated and safe in the ongoing TAK-041-1001 study. TAK-041 continues to be evaluated in the ongoing SRD/multiple-rising dose study (Study TAK-041-1001), and the safety and exposure data for additional single and multiple doses will be evaluated before the IA for this study is conducted. Pending the results of the IA, which will be conducted after an appropriate number of subjects have completed the study at the 40 mg dose level (as outlined in the SAP), a second dose level of 20 mg (or an alternative higher dose such as 80 mg if supported by emerging compound clinical safety data) will be examined in subjects randomized after the IA to explore dose/exposure relationships on the PD endpoints.

Amended or The crossover design has been selected in order to reduce sample size (N = 24), as

new wording:

well as minimize variability in the endpoints. TAK-041 has been evaluated in a single-rising dose (SRD) **and multiple rising dose (MRD)** trial in healthy volunteers ~~at~~ **with single** doses up to **120 and multiple doses regimen** 40/**20** mg **(loading dose on week 1 followed by 3 weekly maintenances doses) of a** crystalline suspension. During the SRD trial, PK blood samples were collected for

~~146~~**43** days following ~~the 40 mg~~**a single** TAK-041 dose. Preliminary PK data indicated that TAK-041 has a mean terminal elimination half-life (t_1/2_) of approximately 11 days. Therefore, 35 + 7 days between the 2 treatment periods is sufficient to ensure that there is no drug carryover effect. At the end of 35 +7 days, the probability of exceeding the “effect” cut-off is less than 2%, with 17 subjects out of 1000 expected to have plasma concentrations of TAK-041 above 132.5 ng/mL.

## 6.2.2 Rationale for Dose

~~Up to 2 doses of TAK-041 will be used in this study. The first~~**The initial** dose examined in this study will be a single 40 mg dose of TAK-041 crystalline suspension (or matching placebo) administered on Day 1 of each treatment period according to the sequence group assigned~~through the IA. This dose is the highest~~ ~~dose that has been studied thus far in the ongoing TAK-041-1001 first-in-human~~ ~~Phase 1 study. The clinical exposure achieved at this~~. **A 40 mg dose of TAK-041 has been administered to healthy volunteers in the first in human study,**

## TAK-041-1001, and was found to be safe and well-tolerated. During the trial, 6 AEs were observed that were mild in nature, were deemed not related to trial drug, and resolved without treatment. No SAEs and no clinically meaningful changes in safety laboratory results, physical examinations, vital signs, or ECGs were reported for the 16 subjects who received a single dose of TAK-041 crystalline suspension up to 40 mg or matching placebo Dose adjustment will be allowed based on emerging safety, tolerability, and PK data from this study and study TAK-041-1001. Overall, the proposed dose range of TAK-041 is anticipated to achieve sufficient range of plasma exposures with a margin above and beyond the predicted pharmacologically active exposures/concentrations established in preclinical models. Based on the IA results, adjustment to higher doses may be performed to allow for the exploration of the dose-response.

**The clinical exposure achieved at the 40 mg** dose is aligned with predicted human exposure of the highest efficacious dose (1 mg/kg) tested in nonclinical in vivo models of anhedonia and asociality. ~~As discussed in Section~~ [~~4.3~~](#_bookmark20)~~, this dose was found~~ ~~to be well tolerated~~**cognitive impairment associated with schizophrenia (CIAS) and negative symptoms of schizophrenia], and 5 mg/kg on the Nicotine and Amphetamine dopamine release on the nucleus accumbens.** TAK-041 continues to be evaluated in the ongoing SRD/~~multiple-rising dose~~**MRD** study (Study

TAK-041-1001), and the safety and exposure data for additional single and multiple doses will be evaluated~~before the IA for this study is conducted. Pending the results~~ ~~of the IA, which will be conducted after an appropriate number of subjects have~~ ~~completed the study at the 40 mg dose level (as outlined in the SAP), a second dose~~ ~~level of 20 mg (or an alternative higher dose such as 80 mg if supported by emerging~~ ~~compound clinical safety data) will be examined in subjects randomized after the IA~~ ~~to explore dose/exposure relationships on the PD endpoints~~**.**

Preliminary **blinded** results from the ~~first-in-human tria~~l TAK-041-1001 **study**

indicate that single doses of ~~TAK-041 at~~ 5, 10, 20, **40, 80,** ~~and~~ ~~40~~**120 and 160** mg **and multiple dose cohorts 1 (40mg/20mg) and 2 (80 mg/40 mg) consisting of 4 weekly doses, 1 loading dose followed by weekly maintenance doses for 3 weeks** are well tolerated. ~~To fully assess the safety and pharmacokinetic (PK) profile of~~ ~~TAK-041 in subjects receiving 20 and 40 mg TAK-041 doses, follow-up PK blood~~ ~~draws were performed until TAK-041 plasma concentrations were below~~**In** the ~~lower~~ ~~level of quantitation of the assay (<1 ng/ mL). Six adverse events (~~**TAK-041-1001 study, 31** ~~29~~AEs~~)~~ were observed that were **all** mild **and resolved without medication treatment. ~~Twenty seven~~ Thirty of the AEs were** deemed not related to ~~trial~~**study** drug, and resolved without treatment. **For the 66 subjects in the above cohorts there were no** serious ~~adverse events (SAEs),~~**AEs**, no clinically meaningful **abnormalities in** safety laboratory results (including ~~5'-nucleotidase, sorbitol~~ ~~dehydrogenase, and urine osmolality),~~**ALP, AST, ALT, GGT, and TBILI)** and no clinically significant **abnormalities in** physical examination, vital signs, or ~~electrocardiogram (~~ECG results **were** reported**.**~~for the 16 subjects who received 5 to~~ ~~40 mg doses of TAK-041 crystalline suspension or matching placebo in~~

~~TAK-041-1001 single-rising-dose Cohorts 1 and 2.~~ The potential risks can be monitored clinically and/or with laboratory tests and ~~are~~**were** considered in setting up the stopping rules for this clinical trial. Appropriate eligibility criteria that exclude individuals with past history or concurrent conditions that increase the risk ~~will~~ ~~be~~**have been** applied

**Rationale for Change:** To provide rationale for the potential change in dose level. The following sections also contain this change:

- Section [1.0 STUDY SUMMARY](#_bookmark0)
- Section [4.3 Benefit/Risk Profile](#_bookmark20)
- Section [8.1.3 Dose and Regimen](#_bookmark62)

**Change** [**2**](#_bookmark3)**:** [Detailed potential study drug dose level change following the interim analysis.](#_bookmark3)

Initial wording:

## Section 6.1 Study Design

**…**

Effects of up to 2 dose levels and placebo will be assessed. The study is designed to evaluate the effect of TAK-041, a GPR139 orphan receptor agonist, as an add-on to antipsychotics in attenuating the impairment in motivational anhedonia as well as cognitive function using motivation/reward battery tests as well as brain imaging (ie, task-induced fMRI BOLD and ASL) in subjects with stable schizophrenia. Subjects should be stable on their existing medicine (ie, second-generation antipsychotics [SGAs]) for at least 2 months prior to screening. A planned unblinded interim analysis (IA) for efficacy will be conducted when 12 subjects at the 40 mg dose level have completed the Day 14 procedures in both periods. The IA objective is to determine

whether to continue using 40 mg as the dose for TAK-041 after the IA, or revise to a different dose; based on the results of the IA, a second dose level of 20 mg (or an alternative higher dose if supported by emerging compound clinical safety data) will be examined to explore dose/exposure relationships on the PD endpoints.

The study will consist of 2 treatment periods, with a single dose of study drug administered in each period. There will be a35 day (+ 7 days) washout interval between the 2 doses to reduce the potential for residual TAK-041 to impact the PD endpoints. Treatment Period 2 begins at the end of the Treatment Period 1 washout. Because subjects are not confined in this study, they will come to the clinic for the following visits:

Screening Visit (between Days -28 to -2) covering full medical and psychiatric examinations. **…**

The initial dose of TAK-041 will be 40 mg. After the IA, the dose for TAK-041 might be changed for subjects enrolled after the IA, or the initial dose will be continued based on the IA results. The decision criteria will be predefined in the SAP before unblinding.

## …

During the Screening Visit (Days -28 to -2), subjects meeting Diagnostic and Statistical Manual of Mental Disorders fifth edition (DSM-5) criteria for schizophrenia [[4](#_bookmark146)] and on stable antipsychotic therapy for at least 2 months will complete medical and psychiatric examinations, electrocardiogram (ECG), and laboratory safety tests. At the Baseline Assessments Visit (Day -1), subjects will complete practice sessions of the motivation/reward and cognitive battery in order to minimize potential practice effects at subsequent treatment visits, be familiarized to the magnetic resonance imaging (MRI) environment, and further will be tested for baseline assessments (except fMRI). Practice sessions will only be conducted in Period 1. Subjects meeting all inclusion criteria and no exclusion criteria will be administered study medication or placebo in the clinic based on the randomization schedule on Day 1 of Treatment Periods 1 and 2.

On Day 1 of each Treatment Period, following study drug administration, subjects will undergo commencing at 2 hours postdose PD tests for a total of 3 hours 45 minutes and blood samples will be collected at various times after dosing to assess the PK of TAK-041. After the last PK collection at 6 hours postdose, subjects will leave the clinic. On Day 14 of each Treatment Period, subjects will take the second set of PD tests along with a single time point PK collection. Samples for PK will also be collected on Day 49 and Final Visit of second Treatment Period. There will be a washout period of 35 days (+ 7 days) between doses in each Treatment Period to reduce the potential for residual TAK-041 to impact the PD endpoints.

PD testing will be performed in 3 blocks, separated by two 30-minute breaks. The first

block will comprise the Grip Effort Task (15 minutes), Progressive Ratio Test (20

Amended or new wording:

minutes), and BACS (25 minutes). After a 30-minute break, subjects will take the second block that comprises a battery of imaging tests; namely, brain perfusion using noncontrast ASL MRI and fMRI scans which monitor changes in BOLD signal. The fMRI will be run in resting state without a task (rs-fMRI) and with 2-task functional magnetic resonance imaging (t-fMRI) using the MID Reward Task and a Set-Shifting Task. The imaging block will last for approximately 60 minutes. After a 30-minute break, subjects will take the third block which comprises the EEfRT (25 minutes) and the Empathic Accuracy Task (20 minutes).

## …

Blood will be drawn for exploratory PK analysis on Day 1 of each Treatment Period at the following times relative to the TAK-041 or placebo dose: 0 (within 15 min prior to TAK-041/placebo dosing), 1, 3, 4.5, and 6 hours postdose. In addition, single time point PK will be collected in both Periods at the Second Testing Visit (Day 14), Follow-up Visit (Day 49 for Period 2), Final Visit (Day 77 ±7 days), and at early termination if it occurs before Day 49 (for Period 2).

## Section 6.1 Study Design

**…**

Effects of ~~up to 2~~**at least 1** dose ~~levels~~**level** and placebo will be assessed. The study is designed to evaluate the effect of TAK-041, a GPR139 orphan receptor agonist, as an add-on to antipsychotics in attenuating the impairment in motivational anhedonia as well as cognitive function using motivation/reward battery tests as well as brain imaging (ie, task-induced fMRI BOLD and ASL) in subjects with stable schizophrenia. Subjects should be stable on their existing medicine (ie,

second-generation antipsychotics [SGAs]) for at least 2 months prior to screening. **The initial dose level selected will be 40 mg but may be adjusted based on emerging safety, tolerability and PK data.** A planned unblinded interim analysis (IA) for efficacy ~~will~~**may** be conducted when ~~12~~**approximately12** subjects~~at the 40~~ ~~mg dose level~~ have completed the Day 14 procedures in both periods. The IA objective is to determine whether to continue ~~using 40 mg as~~ the ~~dosefor~~**current** TAK-041 dose after the IA, or revise to a different dose~~; based on the results of the IA,~~ ~~a second dose level of 20 mg (either lower or an alternative~~ higher~~dose if supported by~~ ~~emerging compound clinical safety data) will be examined to explore dose/exposure~~ ~~relationships on the PD endpoints.~~

On Day 1 of Period 1, eligible subjects will be randomized in a ratio of 1:1 to 1 of the 2 treatment sequences ([Table 6.a](#_bookmark32)) and will receive each trial drug according to the randomized sequence group. **The randomization will be stratified by the sites.** The initial dose of TAK-041 will be 40 mg~~. After~~ **and may be adjusted prior to** the IA, ~~the~~**depending on available PK and safety data from emerging first**-**in-human cohorts. The** dose for TAK-041 ~~might~~**may also** be changed for subjects enrolled after

the IA~~, or the initial dose will be continued based on the IA results~~. The decision

criteria will be predefined in the SAP before unblinding.

## …

During the Screening Visit (Days -~~28~~**35** to -2), subjects meeting Diagnostic and Statistical Manual of Mental Disorders fifth edition (DSM-5) criteria for schizophrenia [[4](#_bookmark146)] and on stable antipsychotic therapy for at least 2 months will complete medical and psychiatric examinations, electrocardiogram (ECG), and laboratory safety tests. At the Baseline Assessments Visit (Day -1) **for Period 1,** subjects will complete practice sessions of the motivation/reward and cognitive battery in order to minimize potential practice effects at subsequent treatment visits, **and** be familiarized to the magnetic resonance imaging (MRI) ~~environment, and~~ ~~further~~**tasks. At both study periods, subjects** will **also** be tested for **Day -1** baseline assessments (except fMRI). Practice sessions will ~~only~~ be conducted**carried out prior to the baseline assessments** in Period 1 **only**. Subjects meeting all inclusion criteria and no exclusion criteria will be administered study medication or placebo in the clinic based on the randomization schedule on Day 1 of Treatment Periods 1 and 2.

On Day 1 of each Treatment Period, following study drug administration, ~~subjects~~ ~~will undergo~~ **and** commencing at **approximately** 2 hours postdose **subjects will undergo** PD tests for a total of ~~3~~**approximately 4** hours ~~minutesand blood~~. **Blood** samples will be collected at ~~various~~**defined** times after dosing to assess the PK of TAK-041. After the last PK collection at 6 hours postdose, subjects will leave the clinic. On Day 14 of each Treatment Period, subjects will take the second set of PD tests along with a single time point PK collection. Samples for PK will also be collected on Day 49 ~~visit~~ and Final Visit of second Treatment Period. There will be a washout period of 35 days (+ 7 days) between doses in each Treatment Period to reduce the potential for residual TAK-041 to impact the PD endpoints.

PD testing will be performed in 3 blocks, separated by two ~~30-minute~~ breaks. **The test schedule is detailed in the Schedule of Study Procedures, Table 3.a.** The first block will comprise the Grip Effort Task ~~(15 minutes),~~ Progressive Ratio Test ~~(20~~ ~~minutes)~~, and BACS (25 minutes). After a ~~30-minute~~ break, subjects will take the second block that comprises a battery of imaging tests; namely, brain perfusion using non-contrast ASL MRI and fMRI scans which monitor changes in BOLD signal. The fMRI will be run in resting state without a task (rs-fMRI) and with 2-task functional magnetic resonance imaging (t-fMRI) using the MID Reward Task and a Set-Shifting Task. ~~The imaging block will last for approximately 60 minutes.~~ After a ~~30-minute~~ break, subjects will take the third block which comprises the EEfRT ~~(25 minutes)~~ and the Empathic Accuracy Task~~(20 minutes)~~.

## …

Blood will be drawn for ~~exploratory~~ PK analysis ~~on Day 1 of each Treatment Period~~at ~~the following~~ **defined days and** times ~~relative to the TAK-041 or placebo dose: 0~~

~~(within 15 min prior to TAK-041/placebo dosing), 1, 3, 4.5, and 6 hours postdose. In~~

~~addition, single time point PK will be collected in both Periods at the Second Testing~~ ~~Visit (Day 14),~~ **~~Follow~~during the treatment period, follow**-up ~~Visit (Day 49 for~~ ~~Period 2), Final Visit (Day 77 ±7 days),~~and **final visit or** at early termination if it occurs ~~before Day 49 (for Period 2).~~**, as detailed in the Schedule of Study Procedures**, **Table 3.a**.

**Rationale for Change:** To base increase in dose based on safety, tolerability and PK data from emerging TAK-041-1001 study, and interim analysis.

The following sections also contain this change:

- Section [1.0 STUDY SUMMARY](#_bookmark0)
- Section [11.2 Interim Analysis and Criteria for Early Termination](#_bookmark118)

**Change** [**3**](#_bookmark4)**:** [Clarified Period 2 baseline serum pregnancy test collection day.](#_bookmark4)

The primary change occurs in Footnote (c) of Section [3.0 SCHEDULE OF STUDY](#_bookmark15) [PROCEDURES.](#_bookmark15)

Initial wording:

Amended or new wording:

c) The sites will schedule a visit for subjects at the end of the Period 1 Washout. Subjects may return to the sites for this visit any day between Day 35 and 42. This Visit will serve to conduct end of Period 1 procedures *and* the Day -1 Baseline assessments for Period 2. Subjects will return to the clinic the next day for Period 2 dosing and PD testing.

(c) The sites will schedule a visit for subjects at the end of the Period 1 Washout. Subjects may return to the sites for this visit any day between Day 35 and 42. This Visit will serve to conduct end of Period 1 procedures *and* ~~the~~**may also be the same calendar day as** Day -1 Baseline assessments for Period 2. **If P1 Day 35 and P2 Day 1 are the same day, assessments required for both visits only need to be completed once.** Subjects will return to the clinic the next day for Period 2 dosing and PD testing.

Note: In addition, the Table has been expanded to display Period 1 and Period 2 separately.

**Rationale for Change:** The serum pregnancy test was required at the Day 35 visit and the Day-1 visit of each period. Due to the reduction of the washout period from 77 days to 35 days in Amendment 3, P2 Day-1 and P1 Day 35 are equivalent. It has been agreed that this unique sample is called P2 Day 1 and is now consistent with the eCRF.

**Change** [**4**](#_bookmark5)**:** [Revised exclusion criteria for abnormal laboratory values to clarify collection times for](#_bookmark5) [screening and baseline laboratory tests.](#_bookmark5)

The primary change occurs in Section [7.2 Exclusion Criteria.](#_bookmark47)

Initial wording:

Amended or new wording:

## Exclusion criterion 9

Has abnormal Screening or Day -1 laboratory values (>upper limit of normal [ULN] for the respective serum chemistries) of alanine aminotransferase (ALT), aspartate aminotransferase (AST), total bilirubin (TBILI), alkaline phosphatase (ALP),

γ-glutamyl transferase (GGT), 5'-nucleotidase, and/or abnormal urine osmolality, confirmed upon repeat testing.

## Exclusion criterion 9

~~Has~~**The subject has** abnormal Screening or ~~Day -1~~**baseline** laboratory values (>~~upper limit of normal [~~ULN for the respective serum chemistries) of ~~alanine~~ ~~aminotransferase (~~ALT~~), aspartate aminotransferase~~, AST~~), total bilirubin~~ ~~(~~TBILI~~alkaline phosphatase (~~ALP~~), γ-glutamyl transferase (~~GGT~~), 5'-nucleotidase,~~ **confirmed upon repeat testing, 5’nucleotidase (Screening only)** and/or abnormal urine osmolality, confirmed upon repeat testing.

**Rationale for Change:** To state collection days consistent with Section 3.0 Schedule of Study Procedures. In addition, the 5’ nucleotidase assay is not a standard laboratory assessment and requires several days to obtain results. Eligibility determinations based on 5’ nucleotidase results are obtained from Screening only.

**Change** [**5**](#_bookmark6)**:** [Revised exclusion criterion for magnetic resonance imaging contraindication before](#_bookmark6) [imaging assessments.](#_bookmark6)

The primary change occurs in Section [7.2 Exclusion Criteria.](#_bookmark47)

Initial wording:

Amended or new wording:

## Exclusion Criterion 14

Fulfills any of the MRI contraindications on the standard radiography screening questionnaire at the Centre for Neuroimaging Sciences, Institute of Psychiatry, Psychology & Neuroscience, King’s College London (ie, history of surgery involving metal implants, metal body piercing, dentures, dental plates or bridges, any implanted device that is electrically, magnetically, and mechanically activated).

## Exclusion Criterion 14

Fulfills any of the MRI contraindications on the **site** standard radiography screening ~~questionnaire at the Centre for Neuroimaging Sciences, Institute of Psychiatry,~~ ~~Psychology & Neuroscience, King’s College London (ie, history of surgery involving~~ ~~metal implants, metal body piercing, dentures, dental plates or bridges, any implanted~~ ~~device that is electrically, magnetically, and mechanically activated).~~**document.**

**Rationale for Change:** To allow new sites, as TAK-041 2001 has now become a multi-site study, to follow their own process and use their own standard radiography screening questionnaire.

**Change** [**6**](#_bookmark7)**:** [Detailed follow-up laboratory tests required for drug-induced liver injury (DILI).](#_bookmark7) The primary change occurs in Section [10.2.8.4](#_bookmark109).

Initial wording:

Amended or new wording:

## Section 10.2.8.4 Reporting of Abnormal LFTs

If a subject is noted to have ALT or AST elevated >3 ×ULN on 2 consecutive occasions, the abnormality should be recorded as an AE. In addition, a drug-induced liver injury (DILI) eCRF must be completed providing additional information on relevant recent history, risk factors, clinical signs and symptoms and results of any additional diagnostic tests performed.

If a subject is noted to have ALT or AST >3 ×ULN and total bilirubin >2 ×ULN for which an alternative etiology has not been identified, the event should be recorded as an SAE and reported as per Section 10.2.8.3 The investigator must contact the Medical Monitor for discussion of the relevant subject details and possible alternative etiologies, such as acute viral hepatitis A or B or other acute liver disease. Follow-up laboratory tests as described in Section 9.2.9 must also be performed. In addition, a DILI eCRF must be completed and transmitted with the Takeda SAE form (as per Section 10.2.9).

Section 10.2.8.4 Reporting of Abnormal LFTs

If a subject is noted to have ALT or AST elevated >3 ×ULN on 2 consecutive occasions, the abnormality should be recorded as an AE. In addition, a drug-induced liver injury (DILI) eCRF must be completed providing additional information on relevant recent history, risk factors, clinical signs and symptoms and results of any additional diagnostic tests performed.

If a subject is noted to have ALT or AST >3 ×ULN and total bilirubin >2 ×ULN for which an alternative etiology has not been identified, the event should be recorded as an SAE and reported as per Section 10.2.8.3. The investigator must contact the Medical Monitor for discussion of the relevant subject details and possible alternative etiologies, such as acute viral hepatitis A or B or other acute liver disease. Follow-up laboratory tests as described in Section [~~9.2.9~~](#_bookmark85) **9.2.10** must also be performed. In addition, a DILI eCRF must be completed and transmitted with the Takeda SAE form (as per Section ~~10.2.9~~ **9.2.10.2**).

**Rationale for Change:** To correct typographical errors and provide clarity on required follow-up DILI tests.

The following sections also contain this change:

- Section [9.2.10.2 DILI follow-up testing:.](#_bookmark87)

**Change** [**7**](#_bookmark8)**:** [Updated and further clarified excluded medications, supplements, and dietary products](#_bookmark8)

The primary change occurs in Section [7.3 Excluded Medications, Supplements, and Dietary](#_bookmark48) [Products](#_bookmark48).

Amended or new wording:

- Melatonin changed from herbal to its own class.
- Mood stabilizers examples given as Lithium, valproic acid and lamotrigine.
- Medications that may interfere with cognitive function: Such as cold medicines containing antihistamine or dextromethorphan as a cough suppressant are not restricted except for 7 days prior to and during baseline assessments and during PD testing on Day -1~~, Day 1,~~ and Day **1; and 7 days prior to and during the Day** 14 **assessments** in Periods 1 and 2.
- Psychotropic agents known to affect cognition: Such as long-acting sleep aids, amphetamines, barbiturates, lithium, ~~monoamine inhibitors,~~ methylphenidate, anticholinergics, antidepressants (eg, ~~fluoxetinefluvoxamine).~~**tricyclic antidepressants and MAOIs). Note: the definitive guidelines for each medication/class entry in this row is the primary table row for each medication/class.**

**Rationale for Change:** To correct, update and/or clarify entries and examples in Table 7.3 Excluded Medications, Supplements and Dietary Products.

**Change** [**8**](#_bookmark9)**:** [Modified potential number of sites to approximately 4.](#_bookmark9) The primary change occurs in Section [1.0 STUDY SUMMARY.](#_bookmark0)

Initial wording:

Amended or new wording:

## Number of Sites:

Up to 4 sites

## Number of Sites:

~~Up to~~ **~~4~~Approximately 4** sites

**Rationale for Change:** to provide the opportunity to add additional sites if needed to reach the required enrollment.

**Change** [**9**](#_bookmark10)**:** [Introduced randomization stratification by site](#_bookmark10) The primary change occurs in Section [6.1 Study Design.](#_bookmark31)

Initial wording:

**…**

The initial dose of TAK-041 will be 40 mg. After the IA, the dose for TAK-041 might be changed for subjects enrolled after the IA, or the initial dose will be continued based on the IA results. The decision criteria will be predefined in the SAP before unblinding

Amended or new wording:

The trial population will include subjects with stable schizophrenia aged 18-60 years, inclusive, considered eligible on the basis of the trial inclusion and exclusion criteria. The trial will randomize up to 32 subjects to ensure 24 subjects complete. On Day 1 of Period 1, eligible subjects will be randomized in a ratio of 1:1 to 1 of the 2 treatment sequences (**Table 6.a**) and will receive each trial drug according to the randomized sequence group. **The randomization will be stratified by the sites.** The initial dose of TAK-041 will be 40 mg~~. After~~ **and may be adjusted prior to** the IA, ~~the~~**depending on available PK and safety data from emerging first-in-human cohorts. The** dose for TAK-041 ~~might~~**may also** be changed for subjects enrolled after the IA~~, or the initial dose will be continued based on the IA results~~. The decision criteria will be predefined in the SAP before unblinding.

**Rationale for Change:** To maintain the balance between the sequence groups at each site. The following sections also contain this change:

- Section [8.1.8 Randomization Code Creation and Storage](#_bookmark67).

**Change** [**10**](#_bookmark11)**:** [Extended screening period to 35 days prior to dosing.](#_bookmark11) The primary change occurs in Section [6.1 Study Design.](#_bookmark31)

Initial wording:

**…**

- Screening Visit (between Days -28 to -2) covering full medical and psychiatric examinations.

Amended or new wording:

**…**

- Screening Visit (between Days -~~28~~**35** to -2) covering full medical and psychiatric examinations.

**Rationale for Change:** To improve the opportunity for enrollment in this population. The following sections also contain this change:

- Section [1.0 STUDY SUMMARY](#_bookmark0)
- Section [3.0 SCHEDULE OF STUDY PROCEDURES](#_bookmark15)

**Change** [**11**](#_bookmark12)**:** [Updated schedule of study procedures.](#_bookmark12)

The primary change occurs in Section [3.0 SCHEDULE OF STUDY PROCEDURES](#_bookmark15).

Amended or new wording:

Table 3.a Schedule of Assessments was reformatted for clarity:

- Table was split into 2 separate tables by period.
- PK and PD assessments were updated.

**Rationale for Change:** To improve operational use of the table and update, correct and clarify assessment information.

Amendment 04 - A Randomized, Double-Blind, Placebo Controlled, Two-Period Cross-Over, Proof of Activity Study to Evaluate the Effects of TAK-041 on Motivational Anhedonia as Add-On to Antipsychotics in Subjects With Stable Schizophrenia

ELECTRONIC SIGNATURES

| **Signed by** | **Meaning of Signature** | **Server Date**  **(dd-MMM-yyyy HH:mm ‘UTC’)** |
| --- | --- | --- |
| Wu, Jingtao  Faessel, Helene | Biostatistics Approval  Clinical Pharmacology Approval | 13-Aug-2018 17:09 UTC  13-Aug-2018 19:15 UTC |
| Rosen, Laura | Clinical VP Approval | 14-Aug-2018 00:00 UTC |

References

1. Yin, W., et al., *A phase 1 study to evaluate the safety, tolerability and pharmacokinetics of TAK-041 in healthy participants and patients with stable schizophrenia [submitted].* J Clin Pharm, 2021.

2. Ashburner, J., *A fast diffeomorphic image registration algorithm.* NeuroImage, 2007. **38**.

3. Abler, B., S. Erk, and H. Walter, *Human reward system activation is modulated by a single dose of olanzapine in healthy subjects in an event-related, double-blind, placebo-controlled fMRI study.* Psychopharmacology (Berl), 2007. **191**(3): p. 823-33.

4. Siegel, J.S., et al., *Statistical improvements in functional magnetic resonance imaging analyses produced by censoring high-motion data points.* Hum Brain Mapp, 2014. **35**(5): p. 1981-96.

5. Power, J.D., et al., *Spurious but systematic correlations in functional connectivity MRI networks arise from subject motion.* NeuroImage, 2012. **59**(3): p. 2142-2154.

6. Keefe, R.S.E., et al., *The Brief Assessment of Cognition in Schizophrenia: reliability, sensitivity, and comparison with a standard neurocognitive battery.* Schizophrenia Research, 2004. **68**(2): p. 283-297.

7. Chianetta, J.M., et al., *Comparative psychometric properties of the BACS and RBANS in patients with schizophrenia and schizoaffective disorder.* Schizophr Res, 2008. **105**(1-3): p. 86-94.
