# Supplementary material for: The GPR139 agonist TAK-041 produces time-dependent alterations to cerebral blood flow and reward system function in patients with schizophrenia: a randomised placebo-controlled trial
Source: Psychopharmacology (Berl). 2025 Aug 16;243(4):819–29. doi: 10.1007/s00213-025-06884-x (PMC13035629; doi:10.1007/s00213-025-06884-x)
Supplement: Supplementary file 2 — Supplementary Material 2 [file 213_2025_6884_MOESM2_ESM.doc]

**
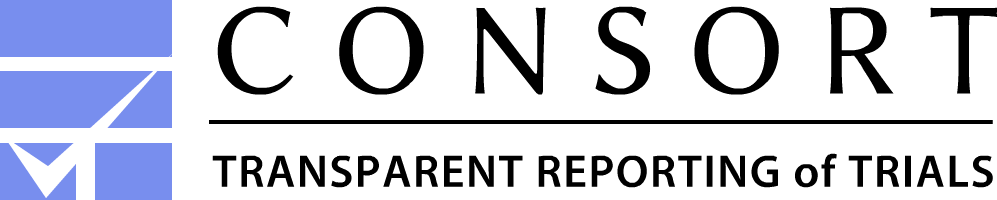
**

**CONSORT 2010 Flow Diagram**

**Allocation**

**Analysis**

**Follow-Up**

**Enrollment**

Assessed for eligibility (n=66)

Excluded (n=43)

♦  Not meeting inclusion criteria (n=43)

♦  Declined to participate (n=0)

♦  Other reasons (n=0)

Analysed Day 1 MID Bayesian (n=6)
♦ Excluded from analysis (n=0)

Lost to follow-up (n=1)

- 1 unable to attend subsequent sessions

Allocated to TAK041 40mg (n=7)

♦ Received allocated intervention (n=7)

♦ Did not receive allocated intervention (n=0)

Lost to follow-up (n=1)

- 1 inability to complete MRI scan

Allocated to TAK041 160mg (n=16)

♦ Received allocated intervention (n=15)

♦ Did not receive allocated intervention (n=1)

- 1 positive DOA screen

Analysed Day 1 MID Bayesian (n= 14)
♦ Excluded from analysis (n=0)

Randomized (n=23)
